# Characterization of three new antiproliferative shikimic acid derivatives and known pyrrolizidine alkaloids from *Senecio oleosus* guided by diagnostic ions and molecular networking

**DOI:** 10.1007/s11306-026-02492-8

**Published:** 2026-07-01

**Authors:** Nathália da Silva Malaco, Anderson Valdiney Gomes Ramos, Bruno Toschi Valeze, Bianca Del Bianco Sahm, Marta Regina Barrotto do Carmo, Letícia Veras Costa-Lotufo, Maria Helena Sarragiotto, Debora Cristina Baldoqui

**Affiliations:** 1 Departamento de Química, Universidade Estadual de Maringá, Av. Colombo 5790, Maringá, PR Brazil; 2 Departamento de Farmacologia, Instituto de Ciências Biomédicas, Universidade de São Paulo, Av. Prof. Lineu Prestes, 1524, São Paulo, SP Brazil; 3 Departamento de Biologia Geral, Universidade Estadual de Ponta Grossa, Av. Carlos Cavalcanti, 4748, Ponta Grossa, PR Brazil

**Keywords:** Metabolome, Structural elucidation, Dereplication, Asteraceae, Antiproliferative activity

## Abstract

**Introduction:**

The genus *Senecio* comprises more than 1,200 species, at least 25 of which are known to be toxic due to the presence of pyrrolizidine alkaloids. In Brazil, approximately 68 species occur, predominantly in rural and mountainous regions.

**Objective:**

This study was performed to investigate the specialized metabolites of *Senecio oleosus* using isolation and dereplication strategies and to evaluate their antiproliferative activity against tumor cell lines.

**Methods:**

A phytochemical investigation of *S. oleosus* was conducted using various chromatographic techniques. Structural elucidation was based on spectroscopic analyses and comparison with literature data. Dereplication was performed using UHPLC-HRMS/MS. Molecular networking was generated through GNPS2 and analyzed using Cytoscape. Antiproliferative activity was assessed using the MTT assay.

**Results:**

The study led to the isolation of three new esterified shikimic acid derivatives. Dereplication of pyrrolizidine alkaloids was guided by the diagnostic fragment ions at *m/z* 120 and *m/z* 138. Diagnostic ions for esterified shikimic acid derivatives were established based on the fragmentation patterns observed in the MS/MS spectra of the isolated compounds. Molecular networking analysis enabled the annotation of 36 additional putative esterified shikimic acid derivatives. One triesterified shikimic acid derivative exhibited promising antiproliferative activity against the HCT-116 cell line, with an IC_50_ value of 10.5 µg.mL^− 1^ (20.2 µM).

**Conclusions:**

In total, 82 compounds were identified in *S. oleosus*. Except for *N*-oxide retrorsine and *N*-oxide senecionine, all compounds are reported for the first time in this species. Esterified shikimic acid derivatives emerged as the major group of specialized metabolites in *S. oleosus*, and triesterified derivatives demonstrated promising antiproliferative activity.

**Supplementary Information:**

The online version contains supplementary material available at https://doi.org/10.1007/s11306-026-02492-8.

## Introduction

Continuing our studies on Asteraceae species from the Campos Gerais region of Paraná, Brazil, the present work focuses on a species belonging to the tribe Senecioneae. *Senecio* is the largest genus within this tribe, comprising approximately 1,200 to 1,500 species distributed worldwide, and it is one of the few genera found in all five regions with a Mediterranean climate (Kandziora et al., 2017; Arias Cassará et al., 2010).

Phytochemical investigations of *Senecio* species have revealed the presence of toxic pyrrolizidine alkaloids (PAs), which pose potential health risks to both humans and livestock (Mulder et al.,^ 2016^; Mulder et al., 2018; Gottschalk et al., 2020; Burgueño-Tapia et al., 2004). In addition, sesquiterpenes of the eremophilane and furanoeremophilane types are frequently reported in this genus (Arias Cassará et al., 2010; Burgueño-Tapia et al., 2004; Portero et al., 2012; Ruiz-Vásquez et al., 2015). By contrast, esterified shikimic acid derivatives are considered rare taxonomic markers, having been identified in only eight *Senecio* species to date (Bohlmann et al., 1984, 1985, 1986; Cardoso et al., 1987; Dupré et al., 1991; Ndom et al., 2006; Lu et al., 2021; Klein et al., 2022; Wang et al., 2022).

The structural complexity and co-occurrence of isomeric forms of both PAs and shikimic acid derivatives in *Senecio* extracts present a significant analytical challenge for conventional phytochemical isolation. In this context, liquid chromatography tandem mass spectrometry (LC-MS/MS), combined with modern untargeted metabolomics tools, has emerged as a powerful approach to accelerate dereplication and metabolite annotation. Based on their characteristic fragmentation patterns, PAs can be effectively characterized in complex mixtures; for instance, the product ions at *m/z* 120 and *m/z* 138 are well-established diagnostic markers of unsaturated PAs (Lu et al., 2021; Klein et al., 2022; Wang et al., 2022). However, systematizing the MS/MS behavior of these rare shikimic acid esters remains crucial for supporting their high-throughput identification in complex mixtures.

In this study, three new esterified shikimic acid derivatives and three known compounds, including the alkaloid *N*-oxide retrorsine, were isolated from *Senecio oleosus* Vell., a species native to Brazil. Using the diagnostic ions *m/z* 120 and *m/z* 138, six additional PAs were putatively identified. This analytical approach was also applied to the dereplication of esterified shikimic acid derivatives. Fragmentation patterns and diagnostic ions were first established based on the MS/MS spectra of the isolated compounds. Subsequently, molecular networking analysis using the GNPS2 platform enabled the annotation of 36 previously undescribed derivatives. Furthermore, because the search for new natural products with cytotoxic properties remains a key driver in drug discovery, as highlighted by recent investigations of plant-derived compounds with in vitro antitumor activity (Situmorang et al., 2024; Xiao et al., 2025), and given the limited pharmacological data available for *S. oleosus*, the antiproliferative activity of the extract, fractions, and isolated compounds was also evaluated.

## Materials and methods

### General experimental procedures

Nuclear magnetic resonance (NMR) spectra were recorded on a Bruker Avance III HD spectrometer operating at 300 MHz and 75.5 MHz, using CDCl_3_, CD_3_OD, and dimethyl sulfoxide (DMSO)-d_6_ as solvents. For individual compounds, ^1^H NMR spectra were acquired using the parameters set automatically by the instrument, with the number of transients fixed at 128 (nt = 128). For ^13^C NMR analyses and 2D experiments, the acquisition parameters were optimized based on sample concentration. Ultra-high-pressure liquid chromatography (UHPLC) analyses were performed using a Shimadzu Nexera X2 system equipped with a CBM-20 A system controller, two LC-30AD pumps, a CTO-30 A column oven, and a SIL-30AC autosampler. Mass spectrometric data were acquired on a Bruker IMPACT II instrument equipped with an electrospray ionization (ESI) source operating in both positive and negative ionization modes, using a quadrupole time-of-flight analyzer and a multichannel plate detector.

Column chromatography (CC) separations were carried out using silica gel 60 (70–230 mesh; Merck), silica gel flash (230–400 mesh; Merck), and Sephadex LH-20^®^ (Sigma-Aldrich). Thin-layer chromatography was performed on precoated silica gel 60G or 60GF_254_ plates (Merck). Spots were visualized under UV light at 254 and 366 nm and by spraying with a chromogenic reagent consisting of H_2_SO_4_/anisaldehyde/acetic acid (1:0.5:50 mL), followed by heating at 100 °C.

### Plant material

The aerial parts of *S. oleosus* were collected in June 2019 at *Cachoeira da Mariquinha*, Ponta Grossa, Paraná, Brazil (25°11’51.9"S, 49°57’00.7"W). The plant was identified by Dr. Marta Regina Barrotto do Carmo, and a voucher specimen was deposited in the herbarium of the Universidade Estadual de Ponta Grossa (HUPG 22432; SISGEN A326417).

### Extraction and isolation

The aerial parts of *S. oleosus* (148.0 g) were dried at 37 °C ± 2 °C for 72 h, powdered using a knife mill, and exhaustively extracted with ethanol (LabSynth, 99.5%) at room temperature through seven successive extraction cycles. The solvent was evaporated under reduced pressure to yield the crude extract (**SCE**, 17.0 g). An aliquot of the crude extract (15.0 g) was suspended in MeOH_2_O (1:1, 400 mL, v/v) and successively partitioned with *n*-hexane (4 × 100 mL; LabSynth, 100%), dichloromethane (3 × 100 mL; LabSynth, 100%), and ethyl acetate (5 × 100 mL; LabSynth, 99.5%), affording hexane (**SHE**, 5.58 g), dichloromethane (**SDC**, 3.34 g), ethyl acetate (**SEA**, 1.77 g), and hydromethanolic (**SHM**, 3.97 g) fractions.

A portion of the SHE fraction (4.00 g) was subjected to silica gel 60 CC (φ = 4.0 cm × h = 20.0 cm), eluted with a gradient of *n*-hexane, EtOAc, and MeOH of increasing polarity, yielding subfractions SHE-1 to SHE-14. Subfraction SHE-6 (543.1 mg) led to the isolation of compound **1** (Table 1). The SDC fraction (2.20 g) was subjected to silica gel 60 CC (φ = 3.0 cm × h = 25.0 cm), using a similar gradient system, resulting in subfractions SDC-1 to SDC-20. Subfraction SDC-8 (119.2 mg) yielded a mixture of compounds **2** and **3** (Table 1).

**Table 1:** ^1^H and ^13^C NMR data for compounds **1**,** 2** and **3** in CDCl_3_

position	1		2		3	
	δ_H_, (J Hz)^a^	δ_C_^b^	δ_H_, (J Hz) ^a^	δ_C_^b^	δ_H_, (J Hz) ^a^	δ_C_^b^
1	–	130.6	–	131.1	–	130.5
2	6.89–6.91 (1 H; m)	135.9	6.88–6.92 (1 H; m)	135.4	6.88–6.92 (1 H; m)	135.1
3	5.80 (1 H; t; 3,6)	65.4	5.83 (1 H; t; 4.1)	65.1	5.62–5.66 (1 H; m)	67.7
4	5.32 (1 H; d; 4,6)	66.0	5.08 (1 H; dd; 8.6, 4.1)	71.6	4.06 (1 H; dd; 7.7, 4.0)	67.9
5	5.29–5.37 (1 H; m)	67.9	4.24 (1 H; q; 7.3)	65.0	5.31 (1 H; q; 7.5)	68.3
6a 6b	2.44 (1 H; brd; 18.4) 2.89 (1 H; brd; 18.4)	28.4	2.36–2.40 (1 H; m) 2.90 (1 H; d; 17.8)	31.0	2.42–2.45 (1 H; m) 2.90 (1 H; d; 17.8)	27.8
7	–	170.6	–	169.9	–	169.9
1’	–	165.4	–	–	–	165.3
2’	5.65 (1 H; dq; 5.0, 1.3)	115.6	–	–	5.75 (1 H; t; 1.3)	115.4
3’	–	159.0	–	–	–	158.7
4’	2.16 (3 H; d; 1.3)	20.6	–	–	2.17 (3 H; s)	20.3
5’	1.89 (3 H; d; 1.2)	27.7	–	–	1.91 (3 H; t; 1.3)	27.5
1’’	–	165.4	–	165.2	–	165.9
2’’	5.65 (1 H; dq; 5.0, 1.3)	115.3	5.69 (1 H; t; 1.3)	115.0	5.66 (1 H; t; 1.3)	114.9
3’’	–	158.8	–	159.4	–	158.5
4’’	2.16 (3 H; d; 1.3)	20.6	2.20 (3 H; d; 1.3)	20.4	2.17 (3 H; s)	20.3
5’’	1.90 (3 H; d; 1.2)	27.6	1.93 (3 H; d; 1.3)	27.4	1.91 (3 H; t; 1.3)	27.5
1’’’	–	172.9	–	173.3	–	–
2’’’	2.27 (2 H; t; 7.4)	34.4	2.31 (2 H; t; 7.4)	34.2	–	–
3’’’	1.57 (2 H; t; 7.2)	25.1	1.60 (2 H; t)	24.8	–	–
4’’’	1.24 (2 H; s)	29.8	1.25 (2 H; s)	29.6	–	–
5’’’	1.24 (2 H; s)	29.5	1.25 (2 H; s)	29.3	–	–
6’’’	1.24 (2 H; s)	29.2	1.25 (2 H; s)	29.2	–	–
7’’’	1.24 (2 H; s)	29.5	1.25 (2 H; s)	29.0	–	–
8’’’	1.24 (2 H; s)	29.6	1.25 (2 H; s)	29.4	–	–
9’’’	1.24 (2 H; s)	29.8	1.25 (2 H; s)	29.6	–	–
10’’’	1.24 (2 H; s)	32.1	1.25 (2 H; s)	31.8	–	–
11’’’	1.24 (2 H; s)	22.8	1.25 (2 H; s)	22.6	–	–
12’’’	0.87 (3 H; t; 6.9)	14.3	0.88 (3 H; t; 6.8)	14.1	–	–

^a^Data (*δ*) measured at 300 MHz; s = singlet, d = doublet, brd = broad doublet, t = triplet, dd = doublet of doublets, dq = doublet of quartet, m = multiplet and q = quartet. *J* values are in Hz and are omitted if the signals overlapped as multiplets. ^b^Data (*δ*) measured at 75.5 MHz

A portion of the SEA fraction (1.40 g) was subjected to Sephadex LH-20 CC (φ = 2.5 cm × h = 33.0 cm), eluted with H_2_O and MeOH in a gradient of decreasing polarity, resulting in subfractions SEA-1 to SEA-13. Subfraction SEA-2 (197.9 mg) was washed with distilled water and MeOH, and the resulting precipitate afforded compound **4** (44.6 mg). Subfraction SEA-12 was identified as compound **5** (34.9 mg), while compound **6** (19.2 mg) was isolated from subfraction SEA-10 (379.1 mg) after CC on silica flash, using a gradient of CH_2_Cl_2_ and MeOH as eluents of increasing polarity.

### UHPLC-HRMS/MS analysis

UHPLC-HRMS/MS analysis (Figs. S27–S28) was performed for comprehensive metabolite profiling, as previously described for plant extract characterization by Shi et al. (2022). The crude extract (SCE) and the dichloromethane (SDC), ethyl acetate (SEA), and hydromethanolic (SHM) fractions were analyzed using a UHPLC system (Nexera X2; Shimadzu) coupled to a high-resolution quadrupole time-of-flight mass spectrometer (IMPACT II; Bruker Daltonics, USA) equipped with an ESI source maintained at 40 °C. Samples were prepared in MeOH (1.0 mg mL^− 1^), and chromatographic separation was carried out on a C18 column (75 × 2.0 mm i.d.; 1.6 μm Shim-pack XR-ODS III) using H_2_O (solvent A) and acetonitrile containing 0.1% formic acid (solvent B) as the mobile phase. The elution gradient program was as follows: 5% B (0–1 min), 30% B (1–3 min), 95% B (3–8 min), and held at 95% B (8–16 min). The flow rate was 0.2 mL min^− 1^, and the injection volume was 3 µL. Mass spectrometric detection was performed in both positive and negative ionization modes with a capillary voltage of 4500 V, and sodium formate (10 µM) was used for internal calibration. The dry gas was set to 8 L/min at 200 °C, with a nebulizer pressure of 4 bar. Collision-induced dissociation was performed using argon as the collision gas, with collision energies ranging from 5 to 45 eV. Spectra were acquired over a mass range of *m/z* 50–1300 at a scan rate of 5 spectra/s (Ramos et al., 2019). Target ions were automatically selected for MS/MS fragmentation using auto-fragmentation mode. Raw data were processed using DataAnalysis™ 4.3 software (Bruker). Putative identification of compounds was based on the analysis of fragmentation patterns and comparison with literature reports of previously described metabolites from the *Senecio* genus, as well as with spectral databases. Molecular formulae were assigned based on accurate mass measurements, considering a mass error of ≤ 10 ppm.

#### Molecular networking-based annotation

Molecular networking analysis combined with high-resolution mass spectrometry was performed using the GNPS2 platform, which has been widely applied for the dereplication and annotation of natural products in recent studies (Peng et al., 2024; Sheng et al., 2024). The data were submitted to the GNPS2 platform for molecular networking using the classical_networking_workflow. MS-Cluster was applied with a parent mass tolerance of 0.02 Da and a fragment ion tolerance of 0.02 Da to generate consensus spectra. A molecular network was then constructed using a cosine similarity threshold of > 0.7 and a minimum of four matched fragment peaks to define edges between nodes. Prior to library matching, MS/MS spectra were filtered by removing all fragment ions within ± 17 Da of the precursor *m/z*. The resulting network was queried against GNPS2 spectral libraries, with matches accepted only when the cosine score exceeded 0.7 and at least four fragment ions were shared. The final network was exported from GNPS2 and visualized in Cytoscape (Shannon et al., 2003; Yang et al., 2011). Additionally, the fragmentation data were compared with previously reported compounds in the literature for further annotation.

### *In vitro* antiproliferative assay

The antiproliferative assay was performed against three tumorigenic cell lines: HCT 116 (colon carcinoma), 501mel (metastatic melanoma), and MCF-7 (breast adenocarcinoma), using the MTT cell viability method (Mosmann, 1983). Stock solutions of the test samples were prepared in DMSO at concentrations of 10 and 1 mg mL^− 1^. Subsequently, 1× 10^4^ cells/mL (501mel and HCT-116) and 3 × 10^4^ cells/mL (MCF-7) were seeded in 96-well plates. After 24 h of incubation, samples were added at final concentrations of 5 and 50 µg mL^− 1^ (in duplicate), followed by incubation in a controlled CO_2_ atmosphere (5.0%) at 37 °C. Doxorubicin was used as a positive control and DMSO as the vehicle control. After 69 h of incubation, the supernatant was replaced with culture medium containing MTT (0.5 mg mL^− 1^). Three hours later, the supernatant was removed, and the precipitates containing blue formazan were dissolved in 150 µL of DMSO. Absorbance was measured at 570 nm using a BioTek Synergy HTX Multimode Reader. Data analysis was performed using GraphPad Prism v9.0 software. Samples that inhibited cell growth by more than 70% were selected for serial dose–response experiments (0.0016–50 µg mL^− 1^), which were also evaluated using the MTT assay. The half-maximal inhibitory concentration (IC_50_), along with the 95% confidence interval, was calculated by nonlinear regression in GraphPad Prism v9.0. Results are presented as the mean ± standard deviation of three independent experiments.

### SwissADME-based theoretical physicochemical analysis

Theoretical physicochemical descriptors related to membrane permeability and drug-likeness, including lipophilicity (LogP), topological polar surface area, hydrogen bond donor groups, and predicted aqueous solubility, were calculated for compounds **1**–**3** isolated from *S. oleosus* using the SwissADME online platform (http://www.swissadme.ch/), as described by Daina et al. (2017). SMILES strings were used as input.

## Results and discussion

### Isolation and structure elucidation

**Fig. 1:**
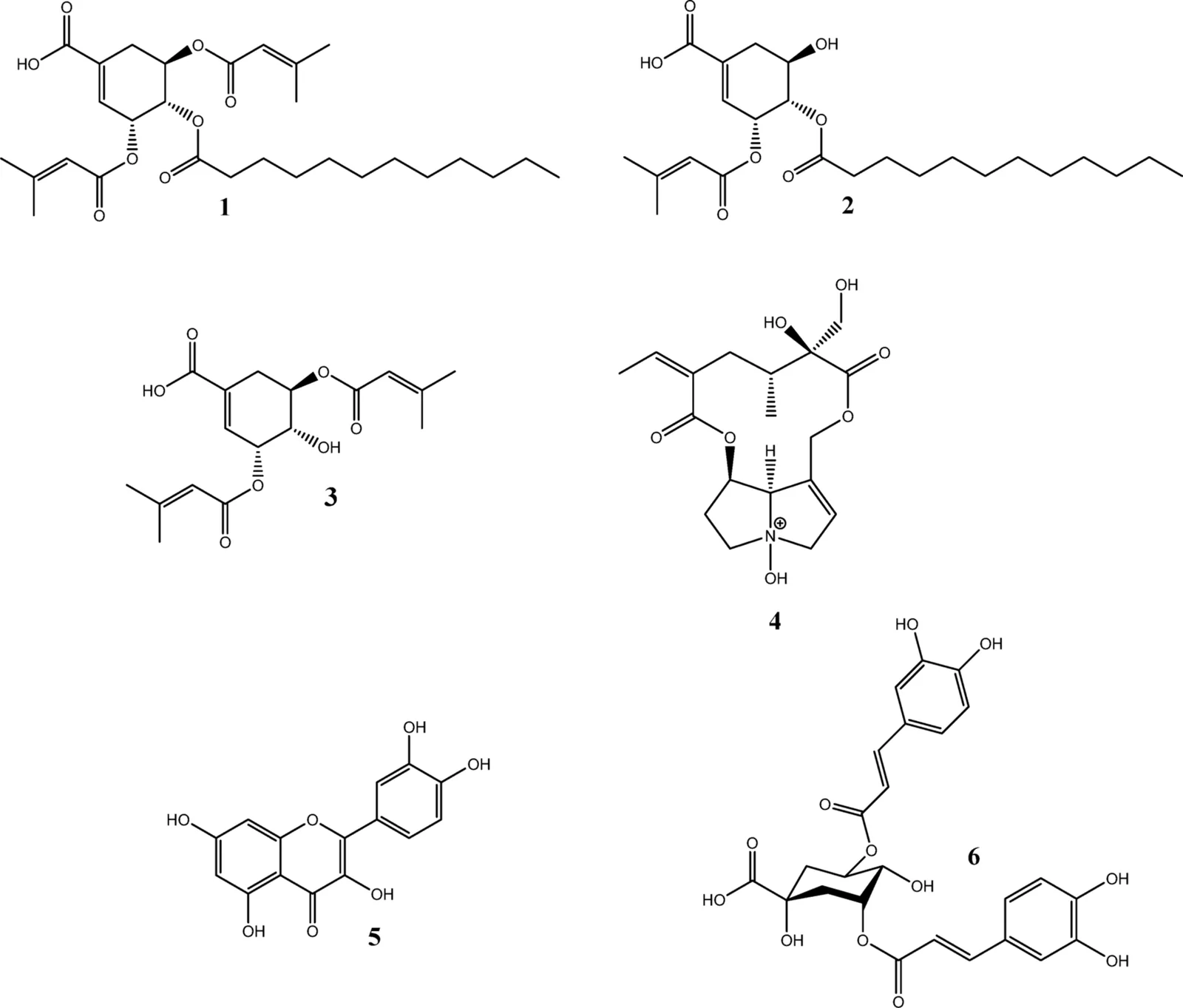
Chemical structures of the compounds isolated from *S. oleosus*

The crude extract from the aerial parts of *S. oleosus* was successively partitioned with *n*-hexane, dichloromethane, and ethyl acetate. Six compounds were isolated, including three new shikimic acid derivatives (**1**, **2**, and **3**) and three known compounds: *N*-oxide retrorsine (**4**), quercetin (**5**), and 3,5-di-*O*-caffeoylquinic acid (**6**) (Fig. 1).

#### Structure elucidation of compound 1

Compound **1** was isolated as an oil from the hexane fraction, and its molecular formula, C_29_H_44_O_8_, was determined by HR-ESIMS data at *m/z* 519.2932 [M − H]⁻ (Fig. S13). The ^1^H NMR spectrum (Table 1; Figs. S1–S4) showed diagnostic signals for a triesterified shikimic acid core. An olefinic multiplet at δ_H_ 6.89–6.91 (1H, m, H-2) confirmed the presence of the characteristic α,β-unsaturated carboxylic system. Three oxymethine proton signals were observed at δ_H_ 5.80 (1H, t, *J* = 3.6 Hz, H-3), δ_H_ 5.32 (1H, d, *J* = 4.6 Hz, H-4), and δ_H_ 5.29–5.37 (1H, m, H-5), indicating esterification at all three hydroxyl groups of the shikimate ring. Additionally, two large doublets at δ_H_ 2.44 (1H, br d, *J* = 18.4 Hz) and δ_H_ 2.89 (1H, br d, *J* = 18.4 Hz) were assigned to the methylene protons H-6a and H-6b. The presence of two senecioyl (3-methylbut-2-enoyl) moieties was evidenced by the olefinic signal at δ_H_ 5.65 (2 H, dq, *J* = 1.3 and 5.0 Hz, H-2′ and H-2″), along with the corresponding vinyl methyl groups at δ_H_ 2.16 (6 H, d, *J* = 3.0 Hz, H-4′ and H-4″) and δ_H_ 1.89 (3 H, d, *J* = 1.2) and δ_H_ 1.90 (3 H, d, *J* = 1.2 Hz, H-5″). The remaining signals at δ_H_ 2.27 (2 H, t, *J* = 7.4 Hz), δ_H_ 1.57 (2 H, t, *J* = 7.2 Hz), δ_H_ 1.24 (16 H, s), and δ_H_ 0.87 (3 H, t, *J* = 6.9 Hz) were assigned to a dodecanoic acid chain.

The ^13^C and HSQC spectra (Figs. S5, S9, and S10) confirmed the shikimic acid scaffold through signals at δ_C_ 130.6 (C-1), δ_C_ 135.9 (C-2), δ_C_ 65.4 (C-3), δ_C_ 66.0 (C-4), δ_C_ 67.9 (C-5), δ_C_ 28.4 (C-6), and δ_C_ 170.6 (C-7). The two senecioyl groups were assigned to signals at δ_C_ 165.4 (C-1′ and C-1″), δ_C_ 115.6 (C-2′), δ_C_ 115.3 (C-2″), δ_C_ 159.0 (C-3′), δ_C_ 158.8 (C-3″), δ_C_ 20.6 (C-4′ and C-4″), and δ_C_ 27.7 (C-5′ and C-5″). Additionally, the dodecanoyl carbonyl group was observed at δ_C_ 172.9 (C-1‴).

**Fig. 2:**
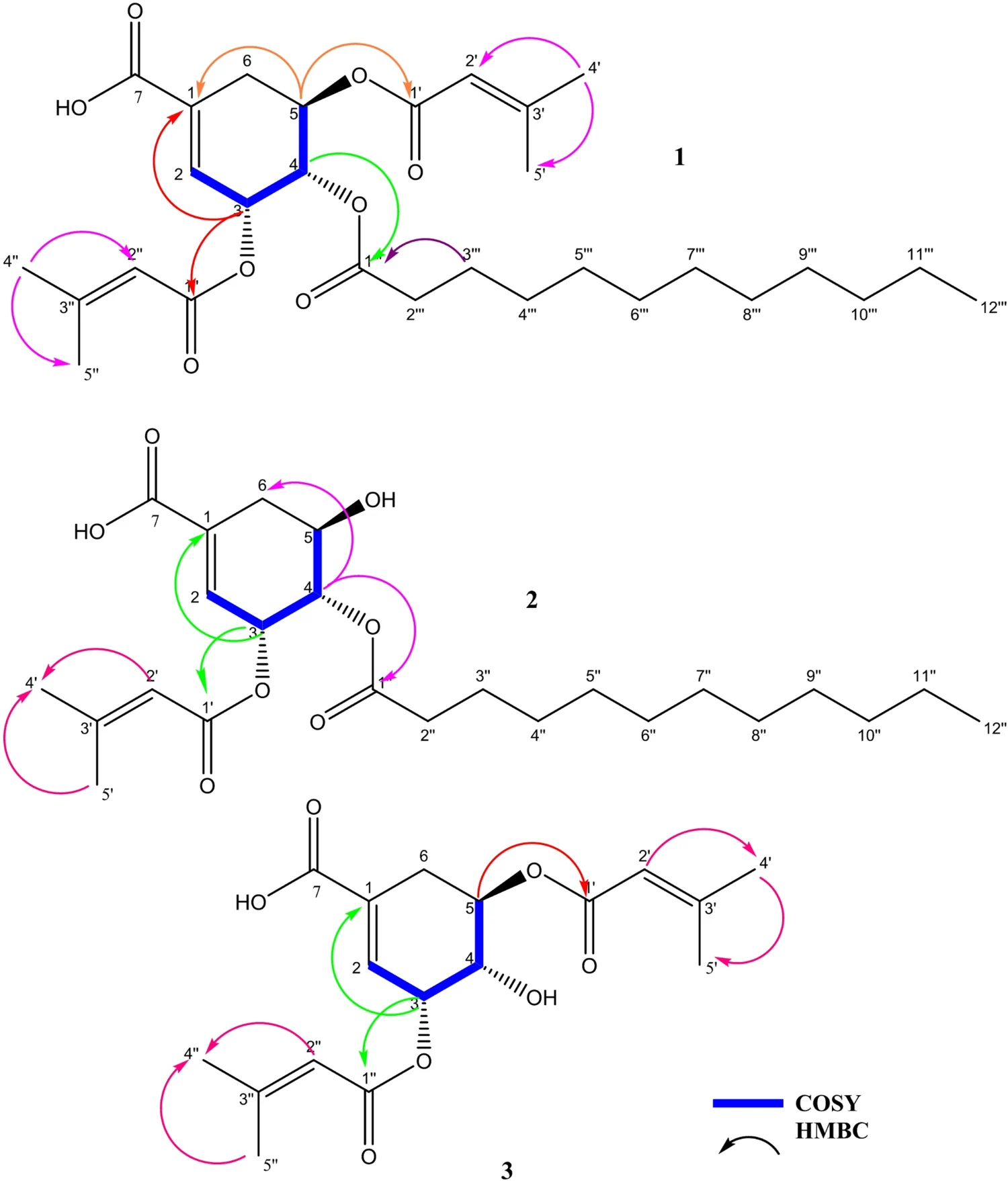
Key correlations observed in the gHMBC and COSY spectra for compounds **1**, **2**, and **3**

The structural connectivity of the ester substituents was unambiguously established through COSY (Figs. S6–S8) and HMBC (Figs. S11 and S12) correlations. In the COSY spectrum, key homonuclear correlations were observed between the olefinic proton H-2 (δ_H_ 6.89–6.91) and the oxymethine H-3 (δ_H_ 5.80). In turn, H-3 coupled with both H-2 and the adjacent oxymethine H-4 (δ_H_ 5.32). The contiguous spin system of the cyclohexene ring was further resolved by the COSY correlation between the oxymethine H-5 (δ_H_ 5.29–5.37) and the geminal methylene protons H-6a and H-6b (δ_H_ 2.44 and 2.89). HMBC correlations were detected between the oxymethine proton H-4 (δ_H_ 5.32) and the lauroyl carbonyl carbon C-1‴ (δ_C_ 172.9), positioning the fatty acid chain at C-4. The positions of the two senecioyl groups were assigned to C-3 and C-5 based on the key HMBC correlations from H-3 (δ_H_ 5.80) to the first senecioyl carbonyl C-1′ (δ_C_ 165.4) and from H-5 (δ_H_ 5.29–5.37) to the second senecioyl carbonyl C-1″ (δ_C_ 165.4). Thus, the spin-coupling networks, combined with key HMBC cross-peaks, confirmed the positions of the ester substituents (Fig. 2).

The length of the carbon chain of the fatty acid moiety attached to C-4 was established by mass spectral analysis. In the MS/MS spectrum, the prominent fragment ion at *m/z* 319 corresponded to the neutral loss of a dodecanoic acid molecule (*m/z* 200, C_12_H_24_O_2_) from the precursor ion [M − H]⁻ at *m/z* 519.2932, leaving the shikimate core esterified with the two remaining senecioyl units. The complementary cleavage of this ester linkage was further supported by the presence of the dodecanoate carboxylate anion at *m/z* 199 (C_12_H_23_O_2_, [M − H]⁻), which was detected as the base peak. Additionally, characteristic product ions observed at *m/z* 137 and *m/z* 93 confirmed the presence of the unsubstituted shikimic acid scaffold, in agreement with fragmentation pathways previously described in the literature for related derivatives (Ruiz-Vásquez et al., 2015).

The relative stereochemistry of compound **1** was established through analysis of the ^1^H NMR coupling constants of the oxymethine protons H-3, H-4, and H-5, which were compared with literature data for structurally related shikimate derivatives (Ruiz-Vásquez et al., 2015; Zhang et al., 2007; Nurazah et al., 2017). Although it was not possible to determine the coupling constant of H-5, the coupling constants of H-3 at δ_H_ 5.80 (*J* = 3.6 Hz) and H-4 at δ_H_ 5.32 (*J* = 4.6 Hz) confirmed that the substituents at C-3, C-4, and C-5 retained the typical 3α,4α,5β relative configuration of the natural shikimic acid scaffold. Consequently, based on the spectroscopic evidence and the regiochemistry of the ester groups established by HMBC analysis, compound **1** was identified as a new triesterified shikimic acid derivative, chemically designated 3α,5β-disenecioyloxy-4α-dodecanoyloxy-shikimic acid (Fig. 1; Table 1).

Compounds **2** and **3** (Fig. 1) were isolated as a mixture. HR-ESIMS analysis revealed deprotonated molecular ions at *m/z* 437.2524 [M − H]⁻ (Fig. S25) and *m/z* 337.1272 [M − H]⁻ (Fig. S26), consistent with the molecular formulas C_24_H_38_O_7_ and C_17_H_22_O_7_, respectively. The ^1^H and ^13^C NMR data (Table 1) indicated that both compounds share structural similarities with compound **1**.

The ^1^H NMR spectrum of compound **2** (Figs. S14–S17) displayed two oxymethine proton signals at δ_H_ 5.83 (1H, t, *J* = 4.1 Hz, H-3) and δ_H_ 5.08 (1H, dd, *J* = 8.6 and 4.1 Hz, H-4), which were highly similar to those observed for compound 1. However, the oxymethine H-5 signal showed a significant upfield shift to δ_H_ 4.24 (1H, q, *J* = 7.3 Hz), confirming the presence of a free hydroxyl group at C-5 and demonstrating that esterification occurred only at C-3 and C-4 of the shikimate ring.

In the COSY spectrum (Figs. S19–S20), key homonuclear correlations resolved the cyclohexene ring spin system (Fig. 2). The positions of the acyl groups were confirmed through heteronuclear correlations in the HMBC spectrum (Figs. S23–S24), where a key cross-peak was detected between the oxymethine proton H-4 (δ_H_ 5.08) and the dodecanoyl carbonyl carbon C-1‴ (δ_C_ 173.3), unambiguously positioning the fatty acid chain at C-4. Additionally, the senecioyl group was positioned at C-3 based on the key HMBC correlation from the oxymethine proton H-3 (δ_H_ 5.83) to the senecioyl carbonyl carbon C-1′ (*δ*_C_ 165.2).

The main structural differences between compounds **2** and **3** lie in the characteristic chemical shifts and coupling constants of the oxymethine protons H-4 and H-5. For compound **3**, these signals appeared at δ_H_ 4.06 (1 H, dd, *J* = 7.7 and 4.0 Hz) and δ_H_ 5.31 (1 H, q, *J* = 7.5 Hz), respectively, which clearly demonstrated the presence of a free hydroxyl group at C-4 and esterification at C-5.

The positions of the acyl groups in compound **3** were confirmed through heteronuclear correlations in the HMBC spectrum (Figs. S23–S24). Key cross-peaks were detected between the oxymethine proton H-3 (δ_H_ 5.62–5.66) and the senecioyl carbonyl carbon C-1″ (δ_C_ 165.9), and between the oxymethine proton H-5 (δ_H_ 5.31) and the second senecioyl carbonyl carbon C-1‴ (δ_C_ 165.3). In addition, the senecioyl group at C-3 was supported by the HMBC correlation from H-3 (δ_H_ 5.83) to C-1′ (*δ*_C_ 165.2) (Fig. 2).

Based on these comprehensive spectroscopic analyses and in agreement with the natural relative stereochemistry of the shikimate core, compounds **2** and **3** were putatively characterized as 3α-senecioyloxy-4α-dodecanoyloxy-shikimic acid and 3α,5β-disenecioyloxy-shikimic acid, respectively.

The three new metabolites isolated from *S. oleosus* expand the small but consistent group of shikimate esters reported in the genus *Senecio*. To date, previously reported *Senecio* shikimate esters are polyacylated shikimic acid derivatives that show considerable variation in their acyl residues (Bohlmann et al., 1985; Cardoso et al., 1987; Dupré et al., 1991; Lu et al., 2021; Klein et al., 2022; Wang et al., 2022). Shikimate esters incorporating medium- or long-chain acyl groups, such as C10 or C18, have only been reported from *S. lividus* (Bohlmann et al., 1986)d *burtonii* (Ndom et al., 2006), respectively, whereas shikimate esters containing a senecioyl group have been reported in *S. hieracioides* (Bohlmann et al., 1984). In this context, the metabolites from *S. oleosus* are distinguished by combining a fatty acyl chain and a senecioyl moiety within the same molecule, thereby expanding the known acylation patterns of this class in the genus.

### Dereplication analysis of pyrrolizidine alkaloids (PAs)derivatives

A molecular network was constructed from 436 precursor ions detected in positive ionization mode and visualized as nodes. As previously described, *N*-oxide retrorsine was isolated from the ethyl acetate fraction, and its mass spectrum, together with reference data from the literature (Lu et al., 2021; Klein et al., 2022; Wang et al., 2022; Mosmann, 1983), served as a standard for the dereplication of PAs in *S. oleosus*.

**Fig. 3:**
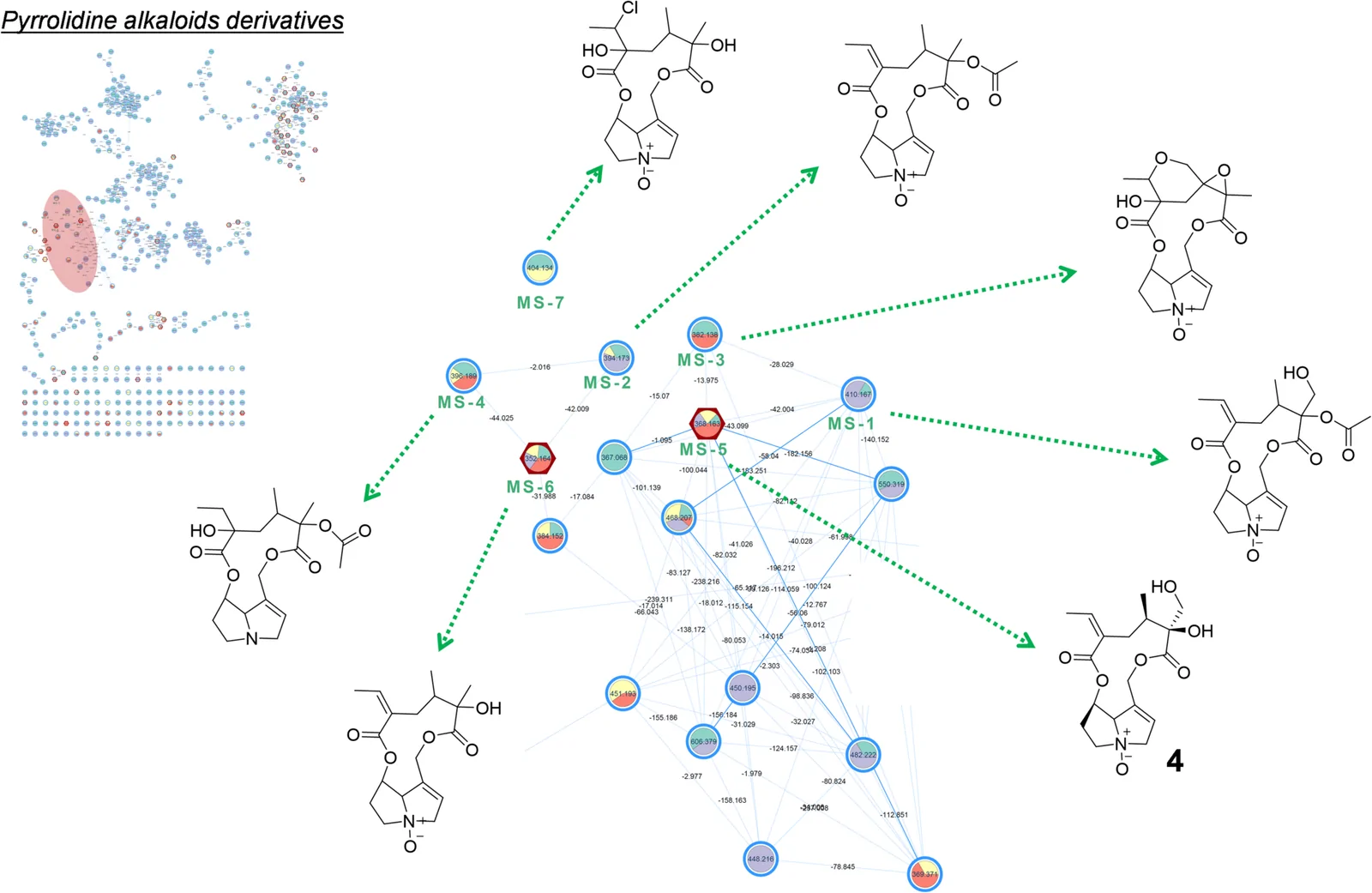
Molecular network of the *Senecio oleosus* extract generated using the GNPS2 platform in positive ionization mode. Nodes are color-coded according to the sample origin: crude extract (green), dichloromethane fraction (purple), ethyl acetate fraction (pink), and hydromethanolic fraction (orange)

Two fragment ions at *m/z* 120 and *m/z* 138 are recognized as diagnostic for retronecine-type PAs in positive ionization mode (Lu et al., 2021; Klein et al., 2022; Wang et al., 2022; Mosmann, 1983). On the GNPS2 platform, the MS^2^ *m/z* highlight tool was applied to visualize nodes exhibiting these diagnostic ions. This filtering highlighted 17 nodes, of which 7 (designated **MS-1** to **MS-7**) were putatively annotated as PAs based on their MS/MS fragmentation patterns and comparison with previously reported compounds in the literature (Fig. 3; Table 2).

**Table 2:** Data of the annotated compounds in the fractions of *Senecio oleosus* by UHPLC-HRMS/MS and molecular networking

Compounds	Molecular formula	Theoretical mass m/z	Precursor ion m/z	Mass error/ppm	t_*R*_/min	Main fragment ions	Putative identification
MS-1	C_20_H_27_NO_8_ [M + H]^+^	410.1809	410.1801	1.9	3.94	368 (b); 340; 138; 136; 120; 118; 94	Retrorsine *N*-oxide Acetate^a^
MS-2	C_20_H_27_NO_7_ [M + H]^+^	394.1860	394.1855	1.2	3.93	352; 324; 138; 120 (b); 94	Senecionine-*N*-oxide acetate^b^
MS-3	C_18_H_23_NO_8_ [M + H]^+^	382.1496	382.1490	1.6	3.89	338; 138 (b); 136; 120; 118; 94	Adonifoline *N*-oxide^a^
MS-4	C_20_H_29_NO_7_ [M + H]^+^	396.2017	396.2004	3.0	3.87	394; 138; 120 (b); 94	Isoline^a^
MS-5	C_18_H_25_NO_7_ [M + H]^+^	368.1704	368.1707	−0.8	3.83	340; 138; 136 (b); 120; 118; 94	Retrorsine *N*-oxide^a^ (**4**)
MS-6	C_18_H_25_NO_6_ [M + H]^+^	352.1755	352.1740	4.2	3.79	324; 138; 120 (b); 94	Senecionine-*N*-oxide^b^
MS-7	C_18_H_26_ClNO_7_ [M + H]^+^	404.1471	404.1442	7.2	3.74	376; 368 (b); 156; 138; 120; 94	Jaconine *N*-oxide^a^

^a^ Lu et al. (2021); ^b^ Klein et al. (2022)

Additional diagnostic fragment ions commonly associated with PAs from the *Senecio* genus were also investigated. These included *m/z* 122 and *m/z* 140, characteristic of saturated retronecine-type PAs, as well as *m/z* 122, *m/z* 150, and *m/z* 168, which are indicative of unsaturated otonecine-type PAs (Lu et al., 2021). However, none of the nodes within the constructed molecular network exhibited these characteristic fragmentation patterns, suggesting the absence of these specific PA subtypes in the analyzed fractions.

### Dereplication analysis of shikimic acid derivatives

A molecular network was generated from 268 precursor ions acquired in negative ionization mode (Fig. 6). As a starting point for the dereplication process, the MS/MS spectra of the esterified shikimic acid derivatives previously isolated from *S. oleosus* (compounds **1**–**3**) were employed as in-house reference standards. Likely due to the limited number of structurally characterized esterified shikimic acid derivatives reported in the literature, only shikimic acid itself (**MS-8**) was annotated in the molecular network through automated library matching on the GNPS2 platform. This sparse representation of such derivatives in conventional spectral databases highlights the importance of integrated molecular networking workflows for enhancing natural product dereplication and metabolite annotation, as demonstrated by Sheng et al. (2024).

**Fig. 4:**
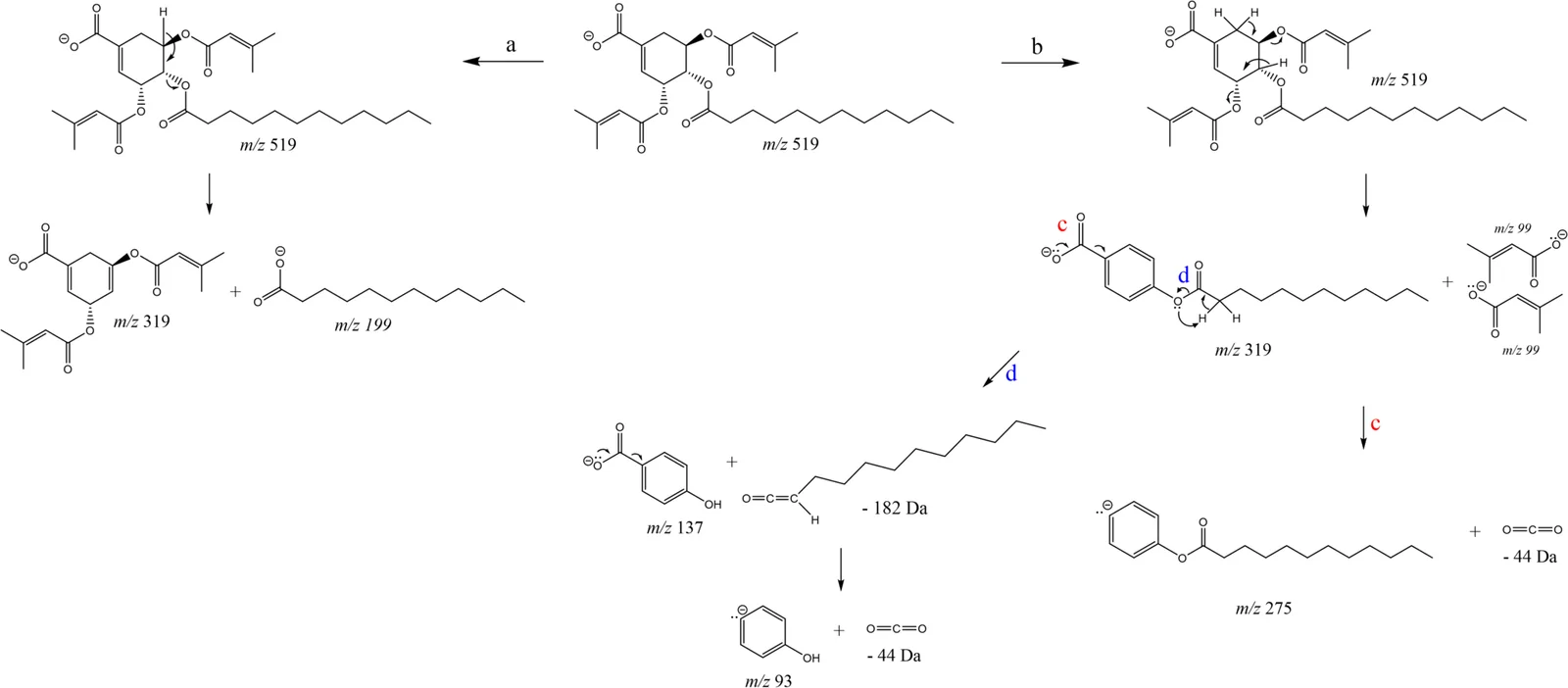
Fragmentation pathway proposed for the shikimic acid derivative **1**

The diagnostic fragmentation pattern for triesterified shikimic acid derivatives was established based on the MS/MS profile of compound **1**, which exhibited consistent and informative fragment ions. Specifically, cleavage of the ester linkages at positions C-3 and C-5 produced a fragment ion at *m/z* 319, corresponding to a shikimate core retaining the ester moiety at C-4. Subsequent loss of CO_2_ yielded a fragment at *m/z* 275. The base peak at *m/z* 199 resulted from cleavage of the fatty acid linked to C-4, while the peak at *m/z* 99 was attributed to the senecioyloxy group. The proposed fragmentation pathway is depicted in Fig. 4.

**Fig. 5:**
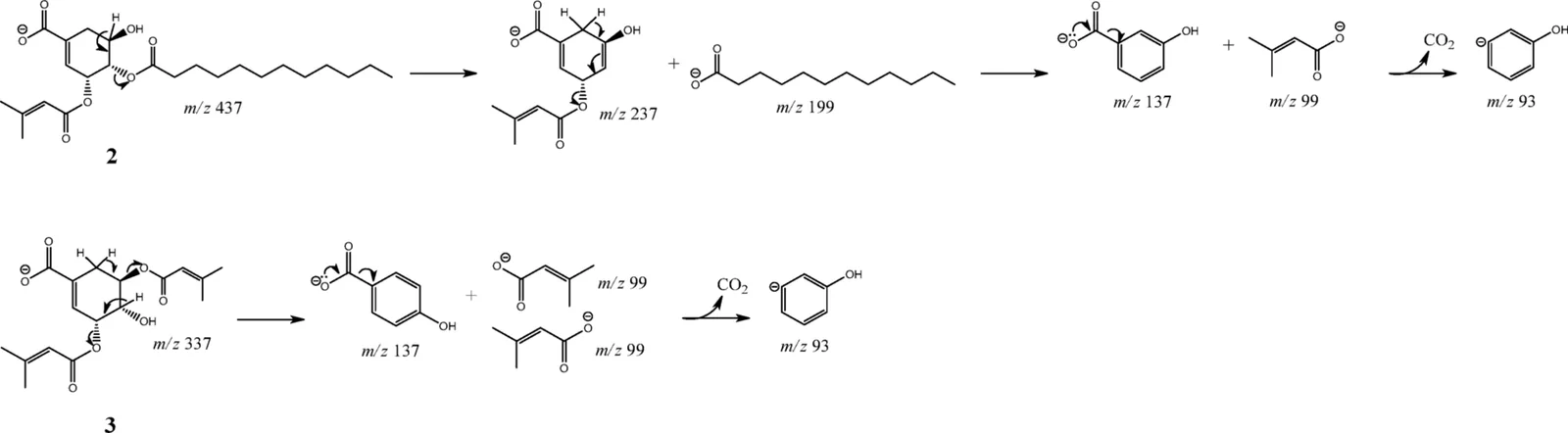
Fragmentation pathway proposed for the shikimic acid derivative **2** and **3**

For compounds **2** and **3**, which are disubstituted shikimic acid derivatives, the base peak was observed at *m/z* 93. In compound **2**, a diagnostic ion at *m/z* 199 was also detected, whereas compound **3** produced characteristic fragments at *m/z* 99 and 137 (Fig. 5).

**Fig. 6:**
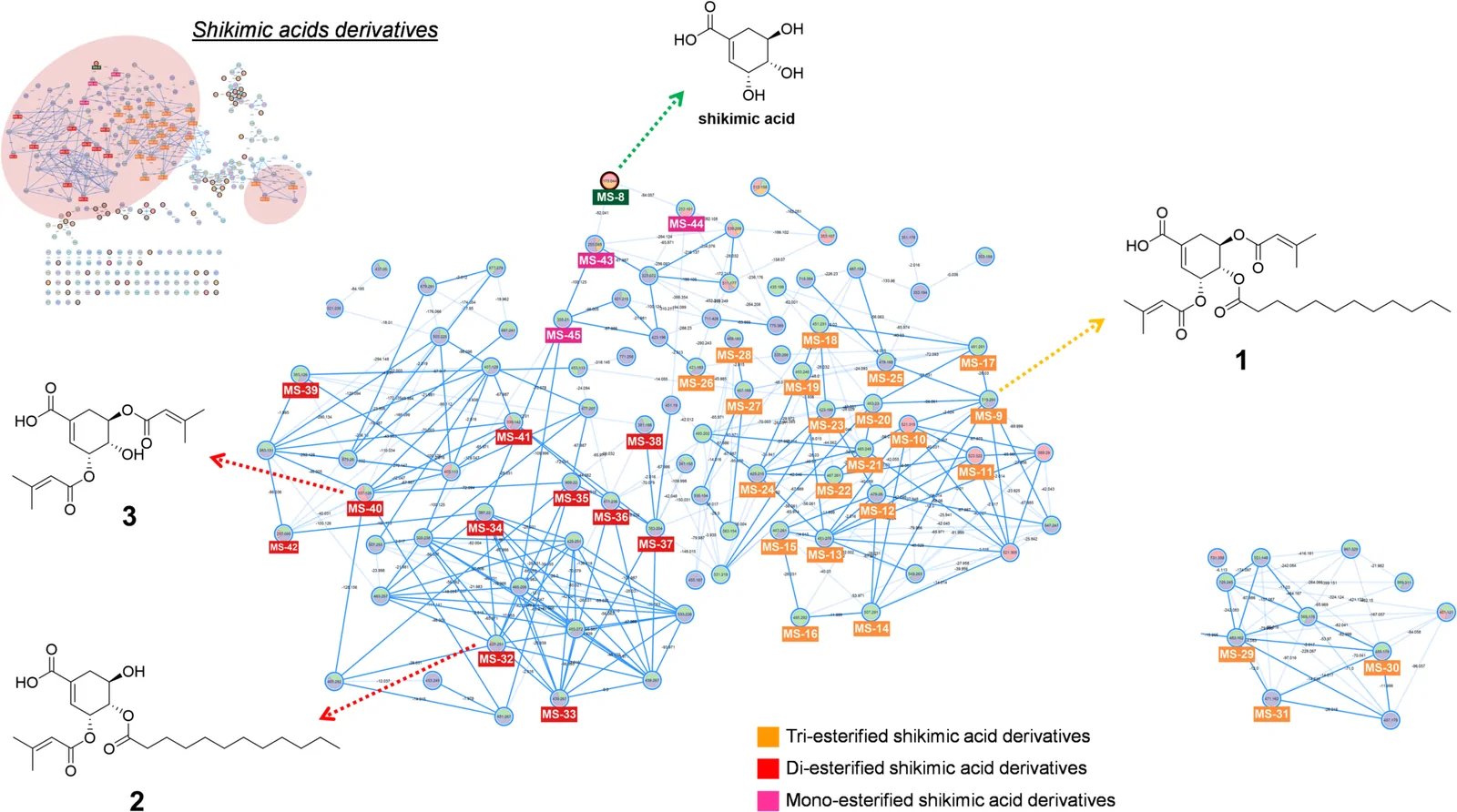
Molecular network of the *Senecio oleosus* extract generated using the GNPS2 platform in negative ionization mode. Nodes are color-coded according to the sample origin: crude extract (green), dichloromethane fraction (purple), ethyl acetate fraction (pink), and hydromethanolic fraction (orange). The highlighted cluster corresponds to putative shikimic acid derivatives. Annotated compounds are indicated within the network, and their proposed chemical structures are provided in the Supplementary Material

Using these fragmentation patterns as a guide, a detailed examination of the molecular network was carried out. A highly connected cluster comprising more than 80 nodes was identified, with several nodes displaying diagnostic fragment ions consistent with those of esterified shikimic acid derivatives (Fig. 6). When the GNPS2 MS^2^ *m/z* highlight tool was applied to emphasize fragment ions at *m/z* 319.18, 275.19, and 199.16, a total of 14 nodes were highlighted, of which 7 were putatively annotated as related compounds (**MS-9** to **MS-16**) (Table 3).

**Table 3:** Putatively annotated compounds (MSI Level 2 and 3) in the dichloromethane fraction of *Senecio oleosus* characterized by UHPLC-HRMS/MS and molecular networking”

Compounds	Molecular formula	Theoretical mass m/z	Precursor ion m/z	Mass error/ppm	t_*R*_/min	Main fragment ions
MS-8 Shikimic acid	C_7_H_10_O_5_ [M - H]^−^	173.0455	173.0449	3.5	0.94	173; 137; 111; 99; 93; 83
*Triesterified shikimic acid derivatives*						
MS-9 (1)	C_29_H_44_O_8_ [M - H]^−^	519.2963	519.2928	6.7	13.87	319; 275; 199 (b); 137; 99; 93
MS-10	C_29_H_46_O_8_ [M - H]^−^	521.3120	521.3083	7.1	8.02	319; 275; 199 (b); 137; 101; 93
MS-11	C_29_H_48_O_8_ [M - H]^−^	523.3276	523.3243	6.3	10.25	319; 275; 199 (b); 112; 101; 93
MS-12	C_26_H_40_O_8_ [M - H]^−^	479.2650	479.2615	7.3	11.78	319; 275; 199 (b); 99; 93
MS-13	C_26_H_42_O_8_ [M - H]^−^	481.2807	481.2782	5.2	12.44	319; 275; 199 (b); 101; 93
MS-14	C_28_H_44_O_8_ [M - H]^−^	507.2963	507.2927	7.1	13.59	319; 275; 199 (b); 99; 93; 87
MS-15	C_25_H_40_O_8_ [M - H]^−^	467.2650	467.2621	6.2	11.64	319; 275; 199 (b); 135; 93; 87
MS-16	C_27_H_44_O_8_ [M - H]^−^	495.2963	495.2930	6.6	13.50	319; 275; 199 (b); 135; 115; 93; 87
MS-17	C_27_H_40_O_8_ [M - H]^−^	491.2650	491.2622	5.7	11.58	291; 247; 171 (b); 137; 99; 93
MS-18	C_24_H_36_O_8_ [M - H]^−^	451.2337	451.2311	5.7	9.71	291; 247; 171 (b); 137; 99; 93
MS-19	C_24_H_38_O_8_ [M - H]^−^	453.2494	453.2469	5.5	10.29	339; 291; 247; 171 (b); 137; 101; 93
MS-20	C_25_H_36_O_8_ [M - H]^−^	463.2337	463.2312	5.4	9.65	263; 255; 219; 205; 143 (b); 99; 93
MS-21	C_25_H_38_O_8_ [M - H]^−^	465.2494	465.2462	6.9	10.21	263; 219; 143 (b); 101; 99; 93
MS-22	C_25_H_40_O_8_ [M - H]^−^	467.2650	467.2616	7.3	10.67	263; 219; 143 (b); 101; 93
MS-23	C_22_H_32_O_8_ [M - H]^−^	423.2024	423.2003	4.9	8.17	263; 219; 143 (b); 99; 93
MS-24	C_22_H_34_O_8_ [M - H]^−^	425.2181	425.2159	5.2	8.59	263; 219; 143 (b); 101; 93
MS-25	C_22_H_28_O_8_ [M - H]^−^	419.1711	419.1688	5.5	7.35	219; 175; 137; 99 (b); 93
MS-26	C_20_H_30_O_8_ [M - H]^−^	421.1868	421.1842	6.2	7.65	199; 177; 155; 137; 111; 101; 93 (b)
MS-27	C_26_H_28_O_8_ [M - H]^−^	467.1711	467.1688	4.9	7.67	267; 223; 147; 137; 99 (b); 93
MS-28	C_26_H_30_O_8_ [M - H]^−^	469.1868	469.1841	5.7	8.00	267; 223; 147; 137; 101 (b); 99
MS-29	C_26_H_28_O_9_ [M - H]^−^	483.1661	483.1631	6.2	6.58	195; 163; 145 (b); 99; 93
MS-30	C_26_H_30_O_9_ [M - H]^−^	485.1817	485.1791	5.3	6.83	195; 163; 145 (b); 119; 101; 99; 93
MS-31	C_25_H_28_O_9_ [M - H]^−^	471.1661	471.1631	6.4	6.48	195; 163; 145 (b); 119; 93; 87
*Diesterified shikimic acid derivatives*						
MS-32 (2)	C_24_H_38_O_7_ [M - H]^−^ C_24_H_38_O_7_ [M – H + HCOOH]^−^	437.2545 483.2600	437.2524 483.2573	4.8 5.6	9.89 9.86	199; 137; 109; 99; 93 (b) 199; 137 (b); 109; 99; 93
MS-33	C_24_H_40_O_7_ [M - H]^−^ C_24_H_40_O_7_ [M – H + HCOOH]^−^	439.2701 487.2756	439.2679 485.2729	5.0 5.6	10.56 10.54	199; 137; 109; 101; 93 (b) 199; 137 (b); 109; 101; 93
MS-34	C_21_H_34_O_7_ [M - H]^−^	397.2232	397.2211	5.3	8.50	199; 137; 109; 93 (b)
MS-35	C_22_H_34_O_7_ [M - H]^−^	409.2232	409.2210	5.4	8.27	171; 137; 109; 99; 93 (b)
MS-36	C_22_H_36_O_7_ [M - H]^−^	411.2388	411.2366	5.3	8.72	247; 171; 152; 137; 109; 101; 93 (b)
MS-37	C_20_H_32_O_7_ [M - H]^−^	383.2075	383.2053	5.7	7.46	143; 137; 109; 101; 93 (b)
MS-38	C_20_H_30_O_7_ [M - H]^−^	381.1919	383.1899	5.2	7,17	143; 137; 109; 99; 93 (b)
MS-39	C_21_H_22_O_7_ [M - H]^−^	385.1293	385.1265	7.3	6.26	147; 137; 99; 93 (b)
MS-40 (3)	C_17_H_22_O_7_ [M - H]^−^ C_17_H_22_O_7_ [2 M - H]^−^	337.1293 675.2658	337.1272 675.2609	6.2 7.2	5.99 6.03	137; 109; 99; 93 (b) 237; 155; 137 (b); 109; 99; 93
MS-41	C_17_H_24_O_7_ [M - H]^−^	339.1449	339.1424	7.4	6.14	137; 99; 101; 93 (b)
MS-42	C_14_H_18_O_7_ [M - H]^−^	297.0980	297.0980	6.4	5.35	252; 197;183;137; 99; 93 (b)
*Monoesterified shikimic acid derivatives*						
MS-43	C_12_H_16_O_6_ [M - H]^−^ C_12_H_16_O_6_ [2 M - H]^−^	255.0874 511.1821	255.0856 511.1780	7.0 8.0	4.98 4.91	179; 155; 137; 111; 99; 93 (b); 83
MS-44	C_12_H_18_O_6_ [M - H]^−^	257.1031	257.1010	8.1	4.91	155; 137; 111; 101; 99; 93 (b); 83
MS-45	C_19_H_32_O_6_ [M - H]^−^	355.2126	355.2109	4.8	7.30	199; 171; 155; 137; 111; 93 (b)

Several nodes closely associated with compound **1** exhibited MS/MS fragmentation patterns featuring diagnostic ions at *m/z* 291, 247, and 171, consistent with structural similarity. These nodes exhibited a systematic mass difference of 28 Da relative to the diagnostic fragments of compounds **MS-9** to **MS-16**, suggesting substitution of the dodecanoyl group at C-4 by a shorter fatty acid chain, most plausibly capric acid (C10).

To support the dereplication of trisubstituted derivatives, three diagnostic ions were defined as follows: M_*di1*_ = 120.02 + M_ac_ – 1, M_*di2*_ = M_*di1*_ – 44 and M_*di3*_ = M_ac_ – 1, where M_ac_ refers to the molecular weight of the acid linked to C-4 and *di* denotes a diagnostic ion. Among these, M_*di3*_ was consistently observed as the base peak. Despite evaluating more than 20 naturally occurring carboxylic acids, only 6 were confidently identified. This strategy facilitated the putative identification of 15 trisubstituted shikimic acid derivatives (**MS-17** to **MS-31**) (Table 3), whose proposed structures are illustrated in Figs. S29–S31, with the corresponding fingerprint ions summarized in Table S1.

In addition to analogs of compound **1**, diagnostic ions such as *m/z* 199, 137, 99, and 93 were recurrent across the MS/MS spectra, enabling the detection and partial structural elucidation of shikimic acid derivatives analogous to compounds **2** and **3**. Furthermore, the defined diagnostic ion logic (M_di1_, M_di2_, and M_di3_) proved instrumental in the dereplication of diesterified derivatives bearing an acid group at C-4. This approach allowed the putative identification of nine disubstituted derivatives (**MS-32** to **MS-42**), as well as three monoesterified derivatives (**MS-43** to **MS-45**) (Table 3). The proposed structures of these compounds are presented in Figs. S32 and S33.

### Dereplication analysis of other specialized metabolites

In addition to PAs and esterified shikimic acid derivatives, the molecular networking approach enabled the annotation of a diverse array of metabolites belonging to distinct chemical classes, including flavonoids, quinic acid derivatives, fatty acids, and saccharides. In total, 37 compounds were tentatively identified through spectral library matching and manual inspection of MS/MS data, comprising 18 flavonoids (**MS-46** to **MS-63**), 6 quinic acid derivatives (**MS-64** to **MS-69**), 4 fatty acids (**MS-70** to **MS-73**), 6 saccharides (**MS-74** to **MS-79**), and 3 compounds belonging to other classes of specialized metabolites (**MS-80** to **MS-82**) (Table 4).

**Table 4:** Data of the annotated compounds in the fractions of *Senecio oleosus* by UHPLC-HRMS/MS and molecular networking

Compounds	Molecular formula	Theoretical mass m/z	Precursor ion m/z	Mass error/ ppm	t_*R*_/ min	Main fragment ions	Putative identification
*Flavonoids*							
MS-46	C_15_H_10_O_6_ [M - H]^−^	285.0405	285.0378	9.5	5.70	285 (b); 229; 187	Kaempferol^b^
MS-47	C_15_H_10_O_5_ [M - H]^−^	269.0455	269.0433	8.3	5.66	269 (b); 181; 151; 117	Apigenin^b^
MS-48	C_15_H_10_O_7_ [M - H]^−^ C_15_H_10_O_7_ [2 M - H]^−^	301.0354 603.0780	301.0350 603.0730	1.2 8.3	5.42 5.42	301; 179; 151 (b); 121 301 (b); 271; 179; 151	Quercetin^b^
MS-49	C_15_H_12_O_6_ [M - H]^−^	287.0561	287.0540	7.3	5.23	285; 177; 125 (b); 93	Dihydrokaempferol^a^
MS-50	C_24_H_22_O_15_ [M - H]^−^	549.0886	549.0853	6.0	4.90	300 (b); 271; 255; 191; 151	Quercetin 3-*O*-malonylglucoside^a^
MS-51	C_20_H_18_O_11_ [M - H]^−^	433.0776	433.0746	7.0	4.87	300 (b); 271; 255; 151	Guajavarin^a^
MS-52	C_21_H_20_O_12_ [M - H]^−^ C_21_H_20_O_12_ [2 M - H]^−^	463.0882 927.1837	463.0850 927.1776	6.9 6.6	4.77 4.77	300 (b); 271; 255 463 (b); 301	Hyperoside^a^
MS-53	C_21_H_20_O_12_ [M - H]^−^	463.0882	463.0865	3.6	4.75	301; 300 (b); 271; 255	Isoquercitin^a^
MS-54	C_26_H_28_O_15_ [M - H]^−^	579.1355	579.1313	7.3	4.70	300 (b)	Quercetin-3-*O*-deoxyhexosyl (1–2) pentoside^a^
MS-55	C_27_H_30_O_16_ [M - H]^−^	609.1461	609.1409	8.5	4.66	301; 300 (b); 271	Rutin^a^
MS-56	C_21_H_20_O_13_ [M - H]^−^	479.0831	479.0799	6.7	4.63	317; 316 (b); 271	Myricetin 3-*O*-galactoside^a^
MS-57	C_17_H_14_O_7_ [M - H]^−^	329.0667	329.0644	6.9	6.18	329; 314; 299 (b); 271	(2*Z*)−4,6-dihydroxy-2-[(4-hydroxy-3,5-dimethoxyphenyl) methylidene]−1-benzofuran-3-one^a^
*Methoxylated flavonoids*							
MS-58	C_18_H_16_O_7_ [M - H]^−^	343.0823	343.0796	7.9	6.28	313 (b); 285	Robinetin trimethyl ether^b^
MS-59	C_18_H_16_O_8_ [M - H]^−^	359.0772	359.0743	8.1	5.82	344; 329 (b); 301; 273; 258	Jaceidin^a^
MS-60	C_16_H_12_O_7_ [M - H]^−^	315.0510	315.0489	6.7	5.52	300 (b); 271; 255; 243	3-*O*-methyl quercetin^a^
MS-61	C_16_H_12_O_7_ [M - H]^−^ C_16_H_12_O_7_ [2 M - H]^−^	315.0510 631.1093	315.0488 631.1049	6.9 7.0	5.50 5.50	300 (b); 271; 255 315; 300 (b)	Tamarixetin^a^
MS-62	C_22_H_22_O_12_ [M - H]^−^	477.1038	477.1000	7.9	4.85	462; 315; 299 (b); 271	Rhamnetin 3-*O*-galactoside
MS-63	C_28_H_32_O_16_ [M - H]^−^	623.1618	623.1591	4.4	4.77	315; 299 (b); 271	Isorhamnetin 3-*O*-rutinoside^a^
*Quinic acid derivatives*							
MS-64	C_34_H_30_O_15_ [M - H]^−^	677.1512	677.148	4.7	5.24	515; 353; 191; 179 (b); 173	3,4,5-tricaffeoyl quinic acid^a^
MS-65	C_25_H_24_O_12_ [M - H]^−^	515.1195	515.1172	4.4	4.93	353; 191 (b); 179; 135	3,5-di-*O*-caffeoyl quinic acid^a, d^
MS-66	C_17_H_20_O_9_ [M - H]^−^	367.1035	367.1013	6.1	4.73	193; 191 (b); 134; 93	3-*O*-feruloylquinic acid^a, d^
MS-67	C_16_H_16_O_9_ [M - H]^−^	351.0722	351.0688	9.8	4.51	191 (b); 173; 133; 93	Chlorogenoquinone^b^
MS-68	C_16_H_18_O_9_ [M - H]^−^	353.0874	353.0854	5.7	4.47	191 (b)	5-*O-*Caffeoylquinic acid^d^
MS-69	C_7_H_12_O_6_ [M - H]^−^	191.0561	191.0550	5.8	0.89	191 (b); 173; 109; 173	Quinic acid^a^
*Fatty acids*							
MS-70	C_18_H_28_O_4_ [M - H]^−^	307.1915	307.1886	9.4	6.18	291; 253; 185; 137; 121 (b); 97	FA 18:4 + 2^b^
MS-71	C_12_H_20_O_4_ [M - H]^−^	227.1289	227.1270	8.2	5.79	209; 183 (b); 157; 68; 56	Traumatic Acid^a^
MS-72	C_18_H_34_O_5_ [M - H]^−^	329.2333	329.2305	8.5	5.74	329; 229; 211 (b); 171; 139	FA 18:1 + 3^b^
MS-73	C_18_H_32_O_5_ [M - H]^−^ C_18_H_32_O_5_ [2 M - H]^−^	327.2177 655.4427	327.2148 655.4392	8.8 5.3	5.63 5.61	229; 211; 183; 171 327; 211 (b); 171	9,12,13-trihydroxy-octadeca-10,15-dienoic acid
*Saccharides*							
MS-74	C_12_H_23_NO_7_ [M - H]^−^	292.1402	292.1382	6.7	1.93	130 (b)	*N*-Fructosyl isoleucine^b^
MS-75	C_12_H_22_O_11_ [M - H]^−^	341.1089	341.1071	5.3	0.91	191 (b); 179; 165; 113; 89	Gentiobiose^b^
MS-76	C_12_H_22_O_11_ [M - H + HCOOH]^−^	387.1144	387.1126	4.6	0.91	341; 119; 113; 89 (b); 71	Turanose^b^
MS-77	C_12_H_22_O_11_ [M - H]^−^	341.1089	341.1071	5.3	0.87	297; 191 (b); 163; 101; 89	Palatinose^b^
MS-78	C_6_H_22_O_7_ [M - H]^−^	195.0510	195.0501	4.6	0.84	195; 151; 129; 105; 99; 87 (b); 75	Gluconic acid^b^
MS-79	C_6_H_12_O_6_ [M - H + HCOOH]^−^	225.0616	225.0608	3.6	0.79	179; 161; 119; 101; 89 (b); 71; 59	α-D-glucose^b^
*Others*							
MS-80	C_15_H_20_O_3_ [M - H]^−^	247.1340	247.1325	6.3	5.84	203 (b); 163; 109	Pechueloic Acid
MS-81	C_46_H_50_N_4_O_8_ [M - H]^−^	785.3556	785.3510	5.8	5.46	665; 545 (b); 145; 119	N1, N5, N10, N14-tetra-*p*-coumaroyl-spermine^c^
MS-82	C_19_H_28_O_10_ [M - H]^−^	461.1664	461.1634	6.5	4.67	300; 231; 191; 179; 149; 101; 89 (b)	Phenyl-ethyl primeveroside

^a^MoNA; ^b^MassBank Europe; ^c^HMDB; ^d^Dai and Sun (2022)

Methoxylated flavonoids have been frequently identified in previous phytochemical studies of Asteraceae species from the Campos Gerais region of southern Brazil (de Oliveira et al., 2017; de Oliveira et al., 2021; Ramos et al., 2019) and have also been reported in *Senecio* species (Ruiz-Vásquez et al., 2015). In the present study, 6 of the 18 tentatively identified flavonoids (**MS-58** to **MS-63**) were methoxylated, corroborating these earlier findings.

One aurone (**MS-57**) was also putatively identified. Aurones are a class of flavonoid-related compounds responsible for the yellow pigmentation of many flowering plants. Considering that *S. oleosus* produces yellow flowers and that the analyzed extract was obtained from the aerial parts of the plant, including flowers, the detection of this compound is consistent with the species’ morphological characteristics.

The remaining metabolites are commonly reported in plants, with the exception of the sesquiterpene pechueloic acid (**MS-80**), also known as rupestonic acid. This compound has previously been reported only in *Artemisia rupestris*, a species belonging to the Asteraceae family, subfamily Asteroideae, which also includes the genus *Senecio*.

### Antiproliferative assays

Cytotoxic properties have been reported for extracts from several *Senecio* species native to Mediterranean regions, including Italy (Conforti et al., 2006; Loizzo et al., 2006) and Algeria (Tidjani et al., 2013), as well as South America, particularly Argentina (Lizarraga et al., 2012). However, this is the first study to investigate the antiproliferative potential of *S. oleosus* collected from the open-field phytogeographic region of southern Brazil.

The antiproliferative assay was conducted using the MTT method to evaluate the cytotoxicity of the extract, fractions, and isolated compounds against HCT-116 (colorectal adenocarcinoma), 501mel (metastatic melanoma), and MCF-7 (breast adenocarcinoma) cells (Table S2). In the initial screening, fractions SCE, SHE, and SDC demonstrated high cytotoxic potential, with the SDC fraction exhibiting inhibition rates of 98.6% ± 1.8% and 61.3% ± 18.4% at concentrations of 50 µg mL^− 1^ and 5 µg mL^− 1^, respectively, against HCT-116 cells. Compound **1** exhibited inhibition rates of 99.6% ± 0.7% and 80.8% ± 12.7% at the same concentrations in the same cell line. Although the mixture of compounds **2** and **3** showed high inhibition at 50 µg mL^− 1^ (96.7% ± 2.1%), its activity was substantially reduced at the lower concentration, with an inhibition rate of 18.8% ± 8.2% at 5 µg mL^− 1^. A similar trend was observed for the SHE fraction, which exhibited significant inhibition at the highest concentration but a reduced effect at the lowest concentration. Very similar activity profiles were observed in the 501mel and MCF-7 cell lines. It is important to note that the new esterified shikimic acid derivatives (**1**–**3**) were isolated from the SHE fraction, whereas 38 esterified shikimic acid derivatives were annotated in the SDC fraction, although none were isolated and tested individually.

The integration of high-resolution mass spectrometry with bioactivity assessment has become an important strategy for exploring the pharmacological potential of plant-derived specialized metabolites (Shi et al., 2022; Su et al., 2022). Beyond dereplication and metabolite annotation, molecular networking approaches can also contribute to integrative pharmacological investigations. Recent studies have combined network pharmacology and computational prediction tools to investigate herb–disease associations and therapeutic mechanisms in medicinal plants (Hu et al., 2023). Therefore, the metabolomic profiling strategy employed herein may provide a promising foundation for future bioactivity-guided studies and systems pharmacology approaches.

Compound **1** showed positive results in the screening experiments and, for that reason, was further evaluated for its cytotoxic potential through serial dilution assays against the three cell lines to determine its IC_50_ value (Table S3, Fig. S34). This triesterified shikimic acid derivative exhibited promising activity, with IC_50_ values of 10.5 µg mL^− 1^ (20.2 µM) in HCT-116, 13.3 µg mL^− 1^ (25.6 µM) in 501mel, and 11.5 µg mL^− 1^ (22.1 µM) in MCF-7 cells. The IC_50_ values obtained for doxorubicin, used as the positive control, were 0.14 µM, 0.11 µM, and 0.34 µM for HCT-116, 501mel, and MCF-7 cells, respectively. According to the United States National Cancer Institute, compounds with IC_50_ values of < 20 µg mL^− 1^ are considered to possess high antiproliferative activity (Damasuri et al., 2020; Sajjadi et al., 2015). Although shikimic acid has previously been associated with antioxidant, anti-inflammatory, and anticancer effects (Rabelo et al., 2016; Sun et al., 2016), this is the first report of the antiproliferative activity of esterified shikimic acid derivatives. The presence of these active compounds in the SHE fraction could explain the observed biological activity against cancer cells, although their exact concentrations in the fraction remain unknown.

The MTT assay is widely employed as an initial approach for evaluating the antiproliferative activity of extracts and isolated natural compounds from diverse sources (Stockert et al., 2018). Nevertheless, despite its broad applicability, this phenotypic assay is limited to detecting changes in cellular physiological behavior and does not provide insight into the molecular mechanisms underlying the observed effects (van Tonder et al., 2015). Consequently, complementary experimental approaches are required to elucidate the intracellular pathways modulated by the bioactive compound and to identify its potential pharmacological targets.

Herein, theoretical physicochemical analyses using SwissADME (Daina et al., 2017) revealed that compound **1** exhibited the highest lipophilicity (LogP = 5.78), the lowest predicted aqueous solubility (LogS = − 6.89), and fewer hydrogen bond donor groups than compounds **2** (LogP = 4.80; LogS = − 5.30) and **3** (LogP = 2.47; LogS = − 2.94). These findings suggest that the greater hydrophobicity associated with the triesterified structure of compound **1** may enhance passive membrane diffusion and intracellular accumulation, which could contribute to its superior antiproliferative activity. Although the topological polar surface area values were similar among the compounds, the higher lipophilicity of compound **1** likely favored membrane permeation and may have played an important role in its antiproliferative effect.

Similar structure–activity relationships have been reported for natural product-derived esters, including long-chain alkyl esters of hydroxycinnamic and ferulic acids (Bakholdina et al., 2019; Menezes et al., 2017), as well as acylated quercetin derivatives (Sudan & Rupasinghe, 2015), in which esterification was associated with enhanced antitumor activity. Furthermore, a recent study on shikimic acid nanoformulations demonstrated anticancer potential through apoptotic induction and oxidative stress modulation in A2058 melanoma cells (Meghdadi et al., 2024). Therefore, the enhanced activity of compound **1** may result from the combined effects of triesterification, long-chain alkyl substitution, and increased lipophilicity, which together may facilitate cellular uptake and improve the inhibition of tumor cell proliferation.

## Conclusions

This work presents the first comprehensive characterization of the specialized metabolome of *S. oleosus*. The isolation of three previously undescribed esterified shikimic acid derivatives, combined with MS/MS-based fragmentation studies, enabled the elucidation of consistent fragmentation pathways and the proposal of diagnostic ions. These molecular markers proved instrumental for the targeted dereplication and annotation of structurally related compounds within this metabolite class.

Furthermore, the present findings highlight the potential of triesterified shikimic acid derivatives as promising scaffolds for the development of new anticancer agents and provide a basis for the rational design and synthesis of novel analogs with improved biological activity.

## Supplementary Information

Below is the link to the electronic supplementary material.

Supplementary Material 1

## Data Availability

The data that support the findings of this study are available in the supplementary material of this article.

## References

[CR1] Arias Cassará, M. L., Borkosky, S., Bardón, A., & Ybarra, M. I. (2010). Two new furanoeremophilanes from *Senecio santelisis*. *Chemistry & Biodiversity,**7*, 1745–1753. 10.1002/cbdv.200900080 20658662

[CR2] Bakholdina, L. A., Markova, A. A., Khlebnikov, A. I., et al. (2019). Cytotoxicity of new ferulic-acid derivatives on human colon carcinoma (HCT116) cells. *Pharmaceutical Chemistry Journal,**53*, 516–520. 10.1007/s11094-019-02030-y

[CR3] Bohlmann, F., Jakupovic, J., & Mohammadi, D. (1984). Shikimic acid derivative from *Senecio hieracioides*. *Journal of Natural Products,**47*(4), 718–720. 10.1021/np50034a028

[CR4] Bohlmann, F., Zdero, C., Jakupovic, J., Grenz, M., Castro, V., Kino, R. M., Robinson, H., & Vincent, L. P. D. (1986). Further pyrrolizidine alkaloids and furoeremophilanes from *Senecio* species. *Phytochemistry,**25*(5), 1151–1159. 10.1016/S0031-9422(00)81571-X

[CR5] Bohlmann, F., Zdero, C., Jakupovic, J., Misra, L. N., Banerjee, S., Singh, P., Baruah, R. N., Metwally, M. A., Schmeda-Hirschmann, G., Vincent, L. P. D., King, R. M., & Robinson, H. (1985). Eremophilane derivatives and other constituents from *Senecio* species. *Phytochemistry,**24*(6), 1249–1261. 10.1016/S0031-9422(00)81111-5

[CR6] Burgueño-Tapia, E., Hernández, L. R., Reséndiz-Villalobos, A. Y., & Joseph-Nathan, P. (2004). Conformational evaluation and detailed 1H and 13C NMR assignments of eremophilanolides. *Magnetic Resonance in Chemistry,**42*, 887–892. 10.1002/mrc.1463 15366063

[CR7] Cardoso, J. M., Jakupovic, J., & Bohlmann, F. (1987). Eremophilane-, bisabolane- and shikimic acid derivatives from Portuguese *Senecio* species. *Phytochemistry,**26*(8), 2321–2324. 10.1016/S0031-9422(00)81709-8

[CR8] Conforti, F., Loizzo, M. R., Statti, G. A., Houghton, P. J., & Menichini, F. (2006). Biological properties of different extracts of two *Senecio* species. *International Journal of Food Sciences and Nutrition,**57*(1–2), 1–8. 10.1080/09637480500131236 16849109

[CR46] Dai, T., & Sun, G. (2021). The analysis of active compounds in Flos Chrysanthemi Indici by UHPLC Q Exactive HF Hybrid Quadrupole-Orbitrap MS and comprehensive quality assessment of its preparati on. Food & Function, 12(4), 1769–1782 10.1039/D0FO03053H 33507197

[CR9] Daina, A., Michielin, O., & Zoete, V. (2017). SwissADME: A free web tool to evaluate pharmacokinetics, drug-likeness and medicinal chemistry friendliness of small molecules. *Scientific Reports,**7*, Article 42717. 10.1038/srep42717 28256516 PMC5335600

[CR10] Damasuri, A. R., Sholikah, E. N., & Mustofa (2020). Cytotoxicity of ((E)-1-(4-aminophenyl)-3-phenylprop-2-en-1-one) on HeLa cell line. *Indonesian Journal of Pharmacy and Therapy*, *1*, 54–59. 10.22146/ijpther.606

[CR11] Dupré, S., Grenz, M., Jakupovic, J., Bohlmann, F., & Niemeyer, H. M. (1991). Eremophilane, germacrane and shikimic acid derivatives from Chilean *Senecio* species. *Phytochemistry,**30*(4), 1211–1220. 10.1016/S0031-9422(00)95204-X

[CR12] Gottschalk, C., Kaltner, F., Zimmermann, M., Korten, R., Morris, O., Schwaiger, K., & Gareis, M. (2020). Spread of *Jacobaea vulgaris* and occurrence of pyrrolizidine alkaloids in regionally produced honeys from Northern Germany: Inter- and intra-site variations and risk assessment for special consumer groups. *Toxins,**12*(9), Article 441. 10.3390/toxins12070441 32645818 PMC7405020

[CR13] Hu, X., Lu, Y., Tian, G., Bing, P., Wang, B., & He, B. (2023). Predicting herb-disease associations through graph convolutional network. *Current Bioinformatics,**18*, 610–619. 10.2174/1574893618666230504143647

[CR14] Kandziora, M., Kadereit, J. W., & Gehrke, B. (2017). Dual colonization of the Palaearctic from different regions in the Afrotropics by *Senecio*. *Journal of Biogeography,**44*, 147–157. 10.1111/jbi.12837

[CR15] Klein, L. M., Gabler, A. M., Rychlik, M., Gottschalk, C., & Kaltner, F. (2022). A sensitive LC–MS/MS method for isomer separation and quantitative determination of 51 pyrrolizidine alkaloids and two tropane alkaloids in cow’s milk. *Analytical and Bioanalytical Chemistry,**414*(28), 8107–8124. 10.1007/s00216-022-04344-5 36183043 PMC9613554

[CR16] Lizarraga, E., Castro, F., Fernández, F., de Lampasona, M. P., & Catalán, C. A. N. (2012). Antioxidant, hemolytic and cytotoxic activities of *Senecio* species used in traditional medicine of Northwestern Argentina. *Natural Product Communications,**7*(5), 1–4. 10.1177/1934578X1200700515 22799087

[CR17] Loizzo, M. R., Tundis, R., Statti, G. A., Miljkovic-Brake, A., Menichini, F., & Houghton, P. J. (2006). Bioactive extracts from *Senecio samnitum*. *Natural Product Research*, *20*(3), 265–269. 10.1080/14786410500077823 16401558

[CR18] Lu, A., Lu, Y., Tan, D., Qin, L., Ling, H., Wang, C., & He, Y. (2021). Identification of pyrrolizidine alkaloids in Senecio plants by liquid chromatography–mass spectrometry. *Journal of Analytical Methods in Chemistry*, *2021*(1957863), 1–13. 10.1155/2021/1957863 PMC8610691 34824876

[CR19] Meghdadi, P., Bamoharram, F. F., Karimi, E., et al. (2024). Shikimic acid nanoformulations: A comprehensive inquiry into anticancer potential and apoptotic induction on A2058 skin cancer cells. *BioNanoScience*, *14*, 2722–2729. 10.1007/s12668-024-01490-1

[CR20] Menezes, J. C. J. M. D. S., Edraki, N., Kamat, S. P., Khoshneviszadeh, M., Kayani, Z., Mirzaei, H. H., Miri, R., Erfani, N., Nejati, M., Cavaleiro, J. A. S., Silva, T., Saso, L., Borges, F., & Firuzi, O. (2017). Long chain alkyl esters of hydroxycinnamic acids as promising anticancer agents: Selective induction of apoptosis in cancer cells. *Journal of Agricultural and Food Chemistry*, *65*(33), 7228–7239. 10.1021/acs.jafc.7b01388 28718636

[CR21] Mosmann, T. (1983). Rapid colorimetric assay for cellular growth. *Journal of Immunological Methods*, *65*, 55–63. 10.1016/0022-1759(83)90303-4 6606682

[CR22] Mulder, P. P. J., de Witte, S., Stoopen, G. M., Van der Meulen, J., Van Wikselaar, P. G., Gruys, E., Groot, M. J., & Hoogenboom, R. L. A. P. (2016). Transfer of pyrrolizidine alkaloids from various herbs to eggs and meat of laying hens. *Food Addit Contam Part A*, *33*, 1826–1839. 10.1080/19440049.2016.1241430 27762672

[CR23] Mulder, P. P. J., López, P., Castelari, M., Bodi, D., Ronczka, S., Preiss-Weigert, A., & These, A. (2018). Occurrence of Pyrrolizidine alkaloids in animal- and plant-derived food: Results of a survey across Europe. *Food Additives & Contaminants: Part A*, *35*, 118–133. 10.1080/19440049.2017.1382726 28942718

[CR24] Ndom, J. C., Mbafor, J. T., Azebaze, A. G. B., Vardamides, J. C., Kakam, Z., Kamdem, A. F. W., Deville, A., Ngando, T. M., & Fomum, Z. T. (2006). Secondary metabolites from *Senecio burtonii* (Compositae). *Phytochemistry*, *67*(8), 838–842. 10.1016/j.phytochem.2006.02.010 16580035

[CR25] Nurazah, Z., Idris, A. S., Kushairi, A., Din, A. M., Abrizah, O., & Ramli, S. (2017). Metabolomics unravel differences between Cameroon Dura and Deli Dura oil palm (Elaeis guineensis Jacq.) genetic backgrounds against basal stem rot. *Journal of Oil Palm Research,**29*, 227–241. 10.21894/jopr.2017.2902.07

[CR26] Oliveira, J. A. M., Bernardi, D. I., Rodolfo, B. B., Avíncola, A. S., Pilau, E., do Carmo, M. R. B., Sarragiotto, M. H., & Baldoqui, D. C. (2017). Chemotaxonomic value of flavonoids in Chromolaena congesta (Asteraceae). *Biochemical Systematics and Ecology,**70*, 7–13. 10.1016/j.bse.2016.10.013

[CR27] Oliveira, J. A. M., Bernardi, D. I., Rodolfo, B. B., Cabral, M. R. P., Zanqueta, E. B., Endo, E. H., Dias Filho, B. P., Nakamura, T. U., Figueiredo, M. C., Ruiz, A. L. T. G., Foglio, M. A., do Carmo, M. R. B., Sarragiotto, M. H., & Baldoqui, D. C. (2021). New cadinene-sesquiterpene from Chromolaena laevigata (Lam.) RM King & H. Rob (Asteraceae) aerial parts and biological activities. *Natural Product Research,**35*(21), 3880–3887. 10.1080/14786419.2020.1747456 32323569

[CR28] Peng, J., Ge, C., Shang, K., Liu, S., & Jiang, Y. (2024). Comprehensive profiling of the chemical constituents in Dayuanyin decoction using UPLC-QTOF-MS combined with molecular networking. *Pharmaceutical Biology,**62*(1), 480–498. 10.1080/13880209.2024.2354341 38808627 PMC11138221

[CR29] Portero, A. G., González-Coloma, A., Reina, M., & Díaz, C. E. (2012). Plant-defensive sesquiterpenoids from Senecio species with biopesticide potential. *Phytochemistry Reviews,**11*, 391–403. 10.1007/s11101-013-9279-3

[CR30] Rabelo, T. K., Guimarães, A. G., Oliveira, M. A., Gasparotto, J., Serafini, M. R., Araújo, A. A. S., Quintans-Júnior, L. J., Moreira, J. C. F., & Gelain, D. P. (2016). Shikimic acid inhibits LPS-induced cellular pro-inflammatory cytokines and attenuates mechanical hyperalgesia in mice. *International Immunopharmacology,**39*, 97–105. 10.1016/j.intimp.2016.07.016 27454847

[CR31] Ramos, A. V. G., Peixoto, J. L. B., Cabral, M. R. P., Amrein, A. M., Tiuman, T. S., Cottica, S. M., Souza, I. M. O., Ruiz, A. L. T. G., Foglio, M. A., do Carmo, M. R. B., Sarragiotto, M. H., & Baldoqui, D. C. (2019). Chemical constituents, antiproliferative and antioxidant activities of Vernonanthura nudiflora (Less.) H. Rob. aerial parts. *Journal of the Brazilian Chemical Society,**30*(8), 1728–1740. 10.21577/0103-5053.20190076

[CR32] Ruiz-Vásquez, L., Reina, M., López-Rodrigues, M., Palomares, J. G. A., Martínez-Díaz, R., & Escobar, R. (2015). Sesquiterpenes, flavonoids, shikimic acid derivatives and pyrrolizidine alkaloids from Senecio kingii Hook. *Phytochemistry,**117*, 245–253. 10.1016/j.phytochem.2015.06.019 26101146

[CR33] Sajjadi, S. E., Ghanadian, M., Haghighi, M., & Mouhebat, L. (2015). Cytotoxic effect of *Cousinia verbascifolia* Bunge against OVCAR-3 and HT-29 cancer cells. *Journal of Herbmed Pharmacology*, *4*, 9–15.

[CR34] Shannon, P., Markiel, A., Ozier, O., Baliga, N. S., Wang, J. T., Ramage, D., Amin, N., Schwikowski, B., & Ideker, T. (2003). Cytoscape software. *Genome Research*, *13*, 2498–2504. 10.1101/gr.1239303 14597658 PMC403769

[CR35] Sheng, Y., Wang, J., Liu, S., & Jiang, Y. (2024). IMN4NPD: An Integrated Molecular Networking Workflow for Natural Product Dereplication. *Analytical Chemistry*, *96*(7), 2990–2997. 10.1021/acs.analchem.3c04746 38324659

[CR36] Shi, S., Li, K., Peng, J., Li, J., Luo, L., Liu, M., Chen, Y., Xiang, Z., Xiong, P., Liu, L., & Cai, W. (2022). Chemical characterization of extracts of leaves of *Kadsura coccinea* (Lem.) A.C. Sm. by UHPLC-Q-Exactive Orbitrap mass spectrometry and assessment of their antioxidant and anti-inflammatory activities. *Biomedicine & Pharmacotherapy,**149*, Article 112828. 10.1016/j.biopha.2022.112828 35339830

[CR47] Situmorang, P. C., Ilyas, S., Nugraha, S. E., Syahputra, R. A., & Nik Abd Rahman, N. M. A. (2024). Prospects of compounds of herbal plants as anticancer agents: A comprehensive review from molecular pathways. *Frontiers in Pharmacology*, *15*, 1387866 10.3389/fphar.2024.1387866 PMC11298448 39104398

[CR37] Stockert, J. C., Horobin, R. W., Colombo, L. L., & Blázquez-Castro, A. (2018). Tetrazolium salts and formazan products in cell biology: Viability assessment, fluorescence imagins, and labeling perspectives. *Acta Histochemistry*, *120*, 159–167. 10.1016/j.acthis.2018.02.005 29496266

[CR38] Su, C. H., Cheng, Y. C., Chang, Y. C., Kung, T. H., Chen, Y. L., Lai, K. H., Hsieh, H. L., Chen, C. Y., Hwang, T. L., & Yang, Y. L. (2022). Untargeted LC-MS/MS-Based Multi-Informative Molecular Networking for Targeting the Antiproliferative Ingredients in *Tetradium ruticarpum* Fruit. *Molecules*, *27*(14), 4462. 10.3390/molecules27144462 35889335 PMC9316527

[CR39] Sudan, S., & Rupasinghe, H. V. (2015). Antiproliferative activity of long chain acylated esters of quercetin-3-*O*-glucoside in hepatocellular carcinoma HepG2 cells. *Experimental Biology and Medicine*, *240*(11), 1452–1464. 10.1177/1535370215570828 25681471 PMC4935297

[CR40] Sun, J., You, C., Dong, K., You, A., & Xing, J. (2016). Anti-inflammatory activity of shikimic acid derivative. *Pharmaceutical Biology*, *54*(10), 2282–2287. 10.3109/13880209.2016.1153663 27609150

[CR41] Tidjani, S., Okusa, P. N., Zellagui, A., Banuls, L. M. Y., Stévigny, C., Duez, P., & Rhouati, S. (2013). Analysis of pyrrolizidine alkaloids and evaluation of some biological activities of algerian *Senecio delphinifolius* (Asteraceae). *Natural Product Communications*. 10.1177/1934578X1300800406 23738446

[CR42] van Tonder, A., Joubert, A. M., & Cromarty, A. D. (2015). Limitations of the 3-(4,5-dimethylthiazol-2-yl)-2,5-diphenyl-2H-tetrazolium bromide (MTT) assay when compared to three commonly used cell enumeration assays. *BMC Research Notes*. 10.1186/s13104-015-1000-8 25884200 PMC4349615

[CR48] Xiao, L., Gong, H., Tan, X., Chen, P., Yang, Y., Zhu, H., & Zhong, S. (2025). Physicochemical characterization and antitumor activity in vitro of a polysaccharide from Christia vespertilionis. International Journal of Biological Macromolecules, 290, 139095 10.1016/j.ijbiomac.2024.139095 39722381

[CR43] Wang, H., Xu, X., Wang, X., Guo, W., Jia, W., & Zhang, F. (2022). An analytical strategy for discovering structural analogues of alkaloids in plant food using characteristic structural fragments extraction by high resolution orbitrap mass spectrometry. *LWT,**154*, Article 112329. 10.1016/j.lwt.2021.112329

[CR44] Yang, X., Yang, L., Xiong, A., Li, D., & Wang, Z. (2011). Authentication of Senecio scandens and S. vulgaris based on secondary metabolic patterns using UPLC-DAD/ESI-MS. *Journal of Pharmaceutical and Biomedical Analysis*, *56*, 165–172. 10.1016/j.jpba.2011.05.004 21664784

[CR45] Zhang, P., Huang, J., & Chen, F. (2007). NMR studies of a series of shikimic acid derivatives. *Journal of the Chinese Chemical Society*, *54*, 1313–1320. 10.1002/jccs.200700185

