# Supplementary material for: Characterization of three new antiproliferative shikimic acid derivatives and known pyrrolizidine alkaloids from Senecio oleosus guided by diagnostic ions and molecular networking
Source: Metabolomics. 2026 Jul 1;22(4):109. doi: 10.1007/s11306-026-02492-8 (PMC13323267; doi:10.1007/s11306-026-02492-8)
Supplement: Supplementary file 1 — Supplementary Material 1 [file 11306_2026_2492_MOESM1_ESM.docx]

**Supporting Information**

**Characterization of three new antiproliferative shikimic acid derivatives and known pyrrolizidine alkaloids from *Senecio oleosus* guided by diagnostic ions and molecular networking**

Nathália da Silva Malaco^1^, Anderson Valdiney Gomes Ramos^1^, Bruno Toschi Valeze^1^, Bianca Del Bianco Sahm^2^, Marta Regina Barrotto do Carmo^3^, Letícia Veras Costa-Lotufo^2^, Maria Helena Sarragiotto^1^, Debora Cristina Baldoqui^1,*^

^1^Departamento de Química, Universidade Estadual de Maringá, Av. Colombo 5790, Maringá, PR, Brazil

^2^ Departamento de Farmacologia, Instituto de Ciências Biomédicas, Universidade de São Paulo, Av. Prof. Lineu Prestes, 1524, São Paulo, SP, Brazil

^3^Departamento de Biologia Geral, Universidade Estadual de Ponta Grossa, Av. Carlos Cavalcanti, 4748, Ponta Grossa, PR, Brazil

Correspondence

Debora C. Baldoqui, Departamento de Química, Universidade Estadual de Maringá, Av. Colombo 5790, 87020-900, Maringá, PR, Brazil. E-mail: dcbaldoqui@uem.br


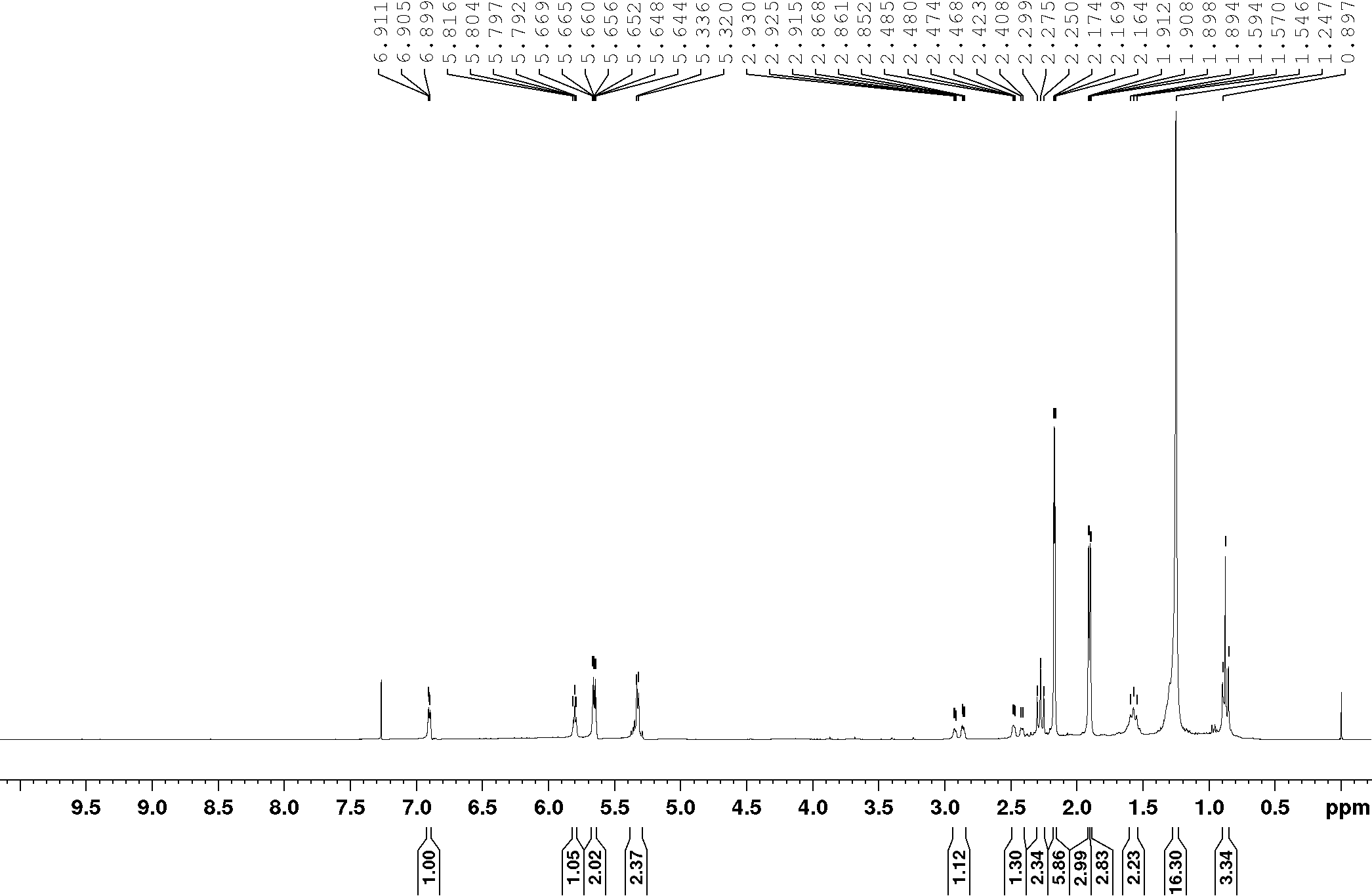


**Figure S1.** ^1^H-NMR spectrum (CDCl_3_, 300 MHz) of compound **1.**


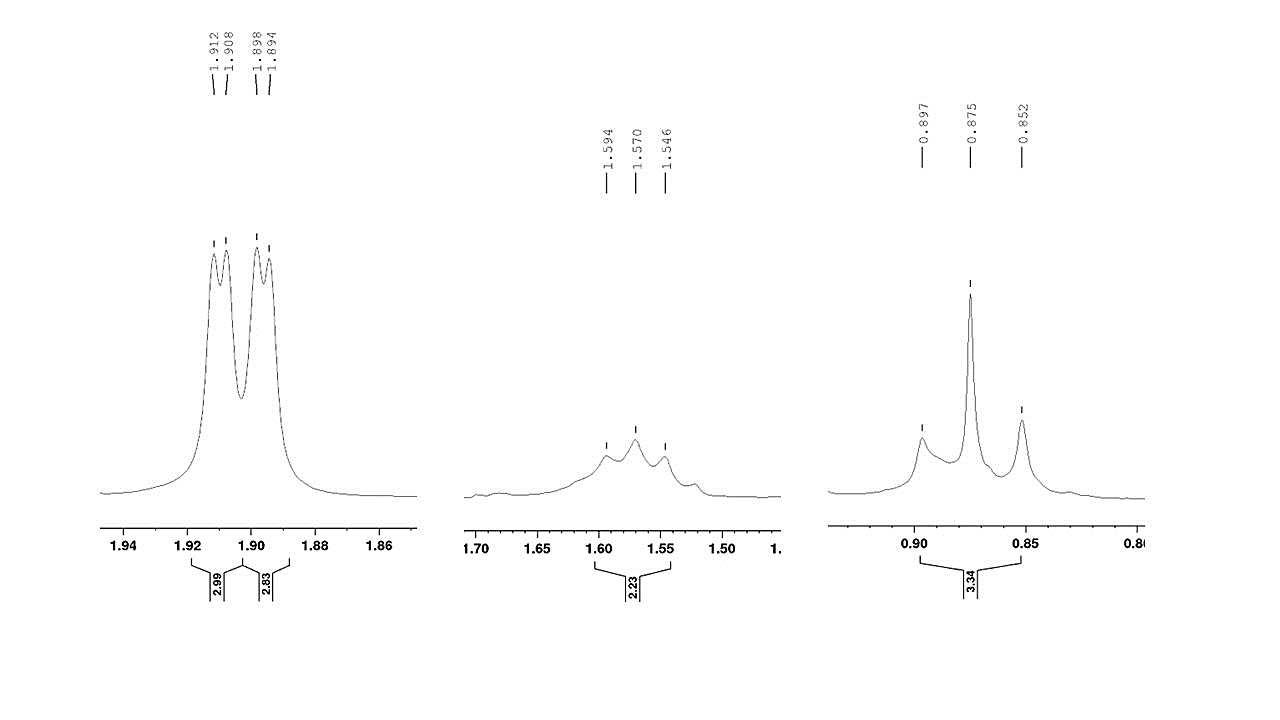


**Figure S2**. Expansions ^1^H-NMR spectrum (CDCl_3_, 300 MHz) of compound **1**.


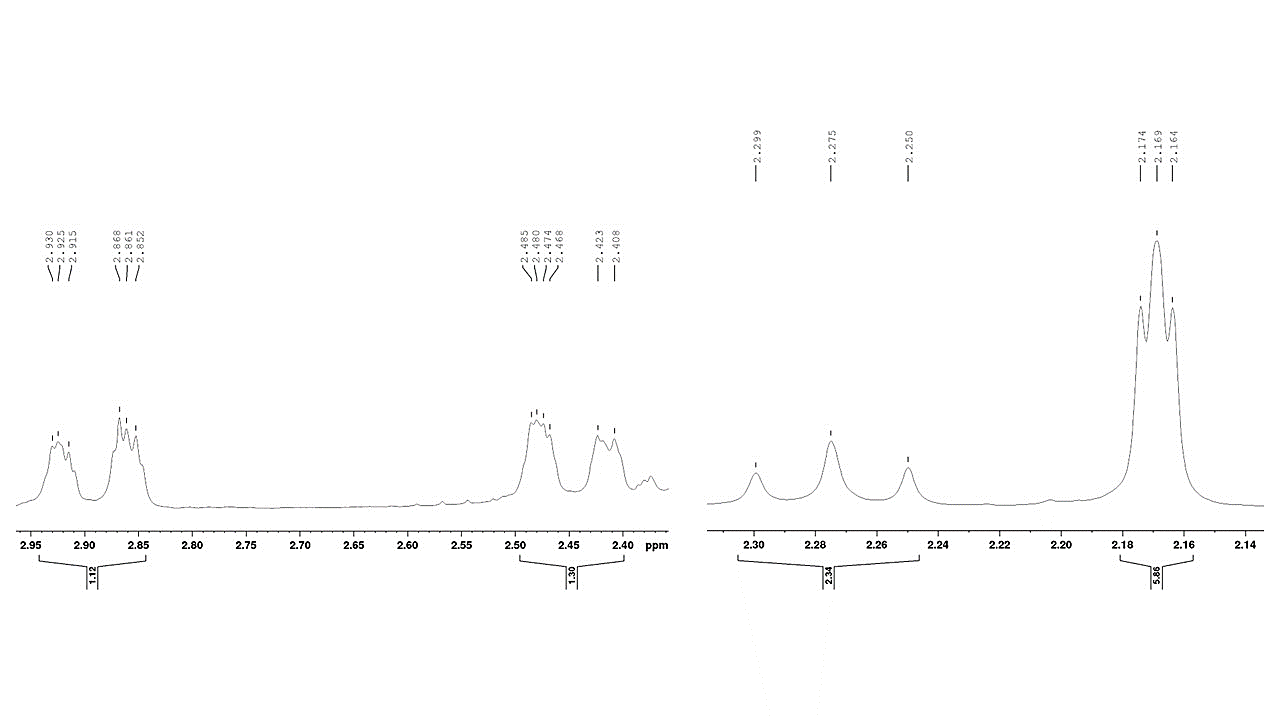
 **Figure S3.** Expansions ^1^H-NMR spectrum (CDCl_3_, 300 MHz) of compound **1**.


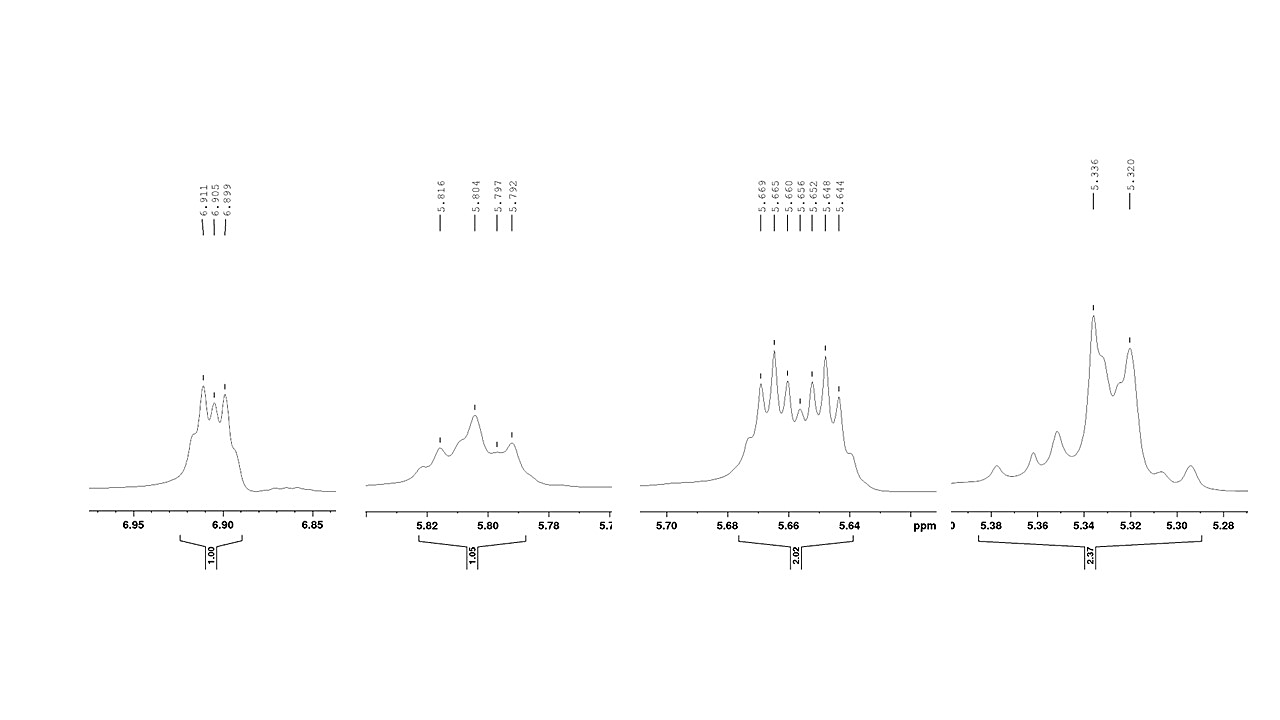
 **Figure S4.** Expansions ^1^H-NMR spectrum (CDCl_3_, 300 MHz) of compound **1**.


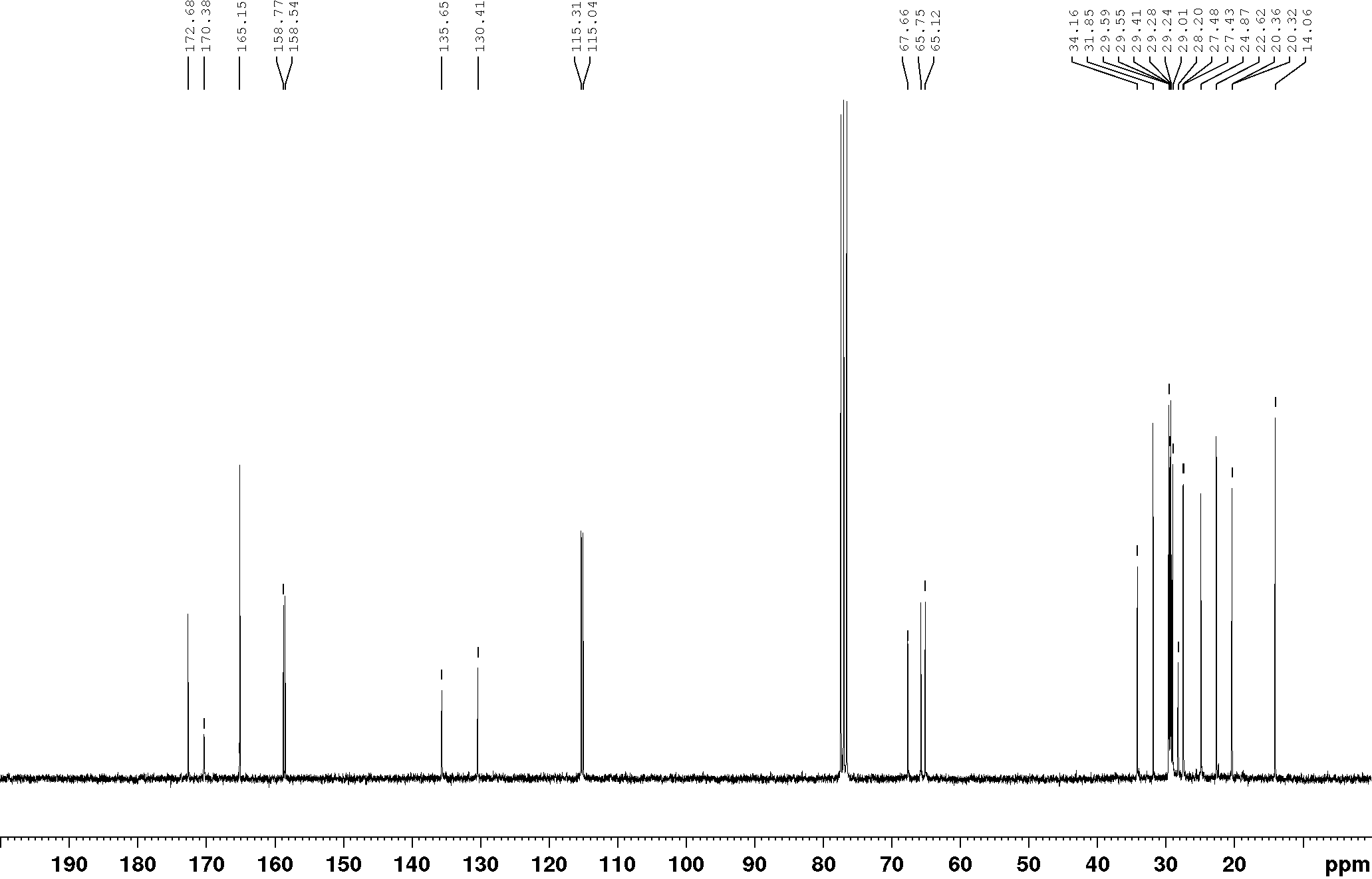
 **Figure S5.** ^13^C-NMR spectrum (CDCl_3_, 75 MHz) of compound **1**.


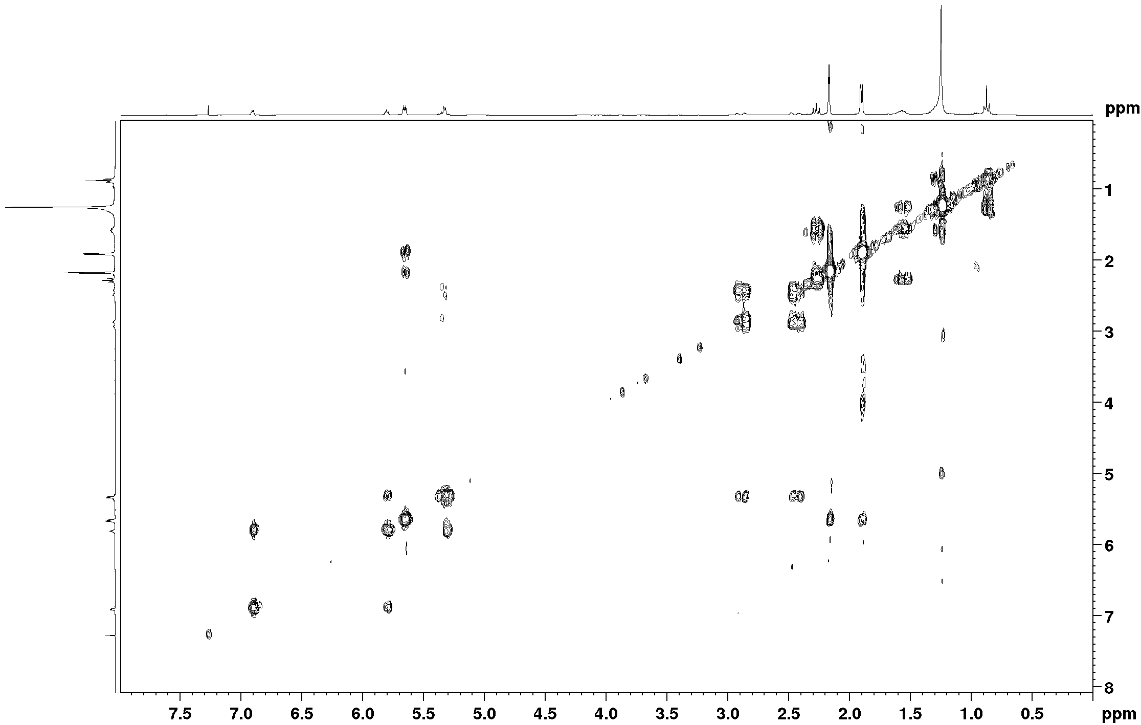
 **Figure S6**. COSY spectrum (CDCl_3_, 300 MHz) of compound **1.**


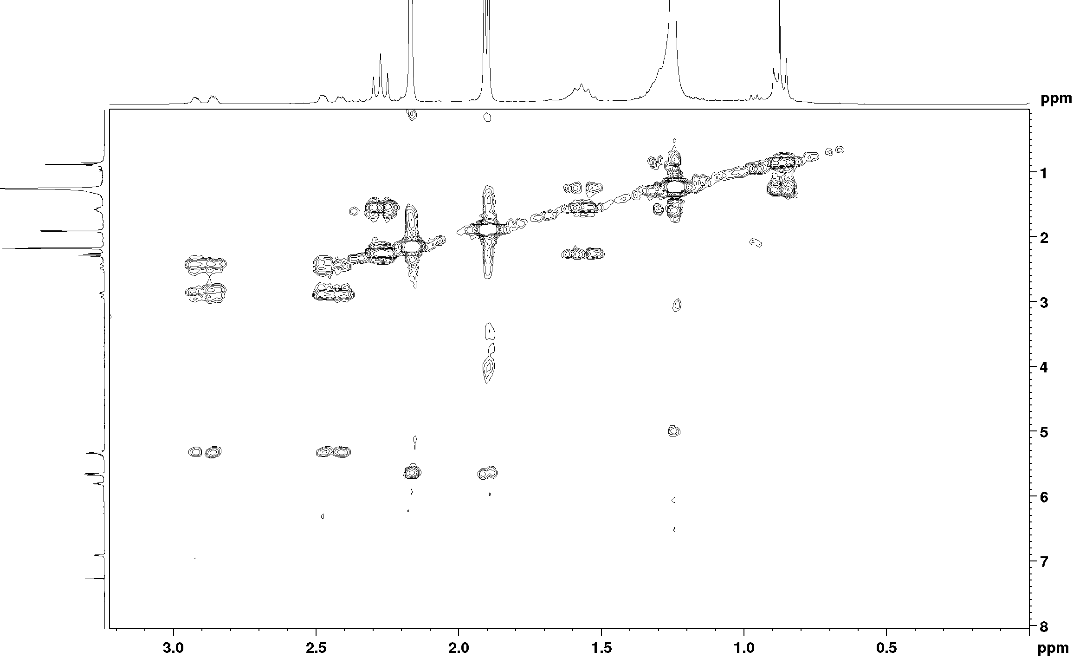
 **Figure S7**. Expansions COSY spectrum (CDCl_3_, 300 MHz) of compound **1.**


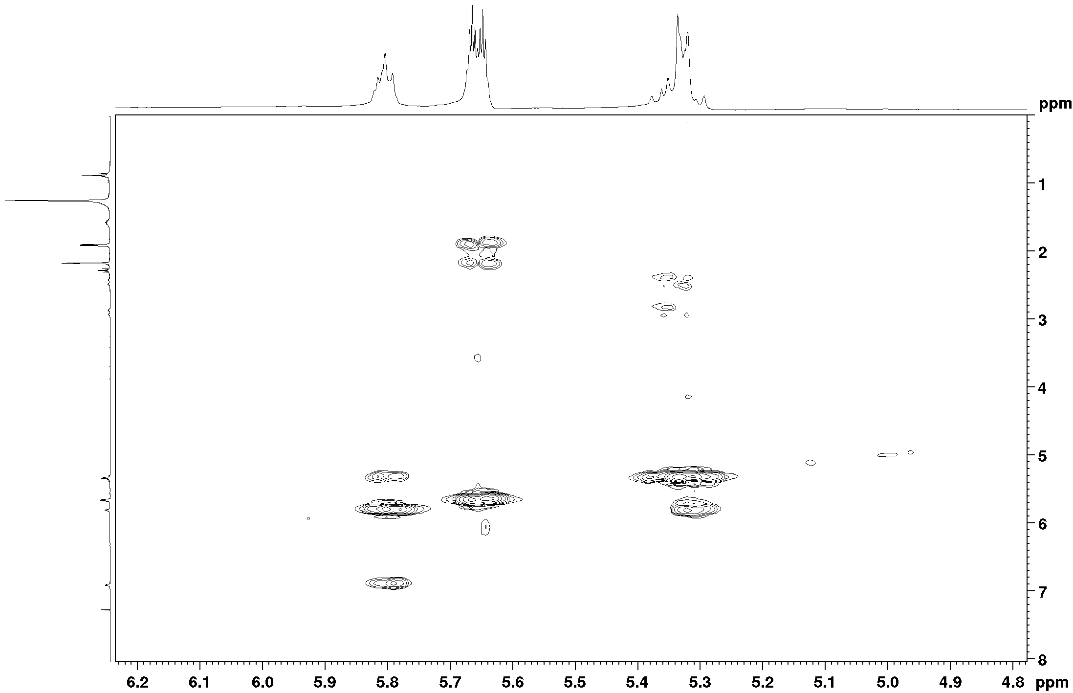
 **Figure S8**. Expansions COSY spectrum (CDCl_3_, 300 MHz) of compound **1.**


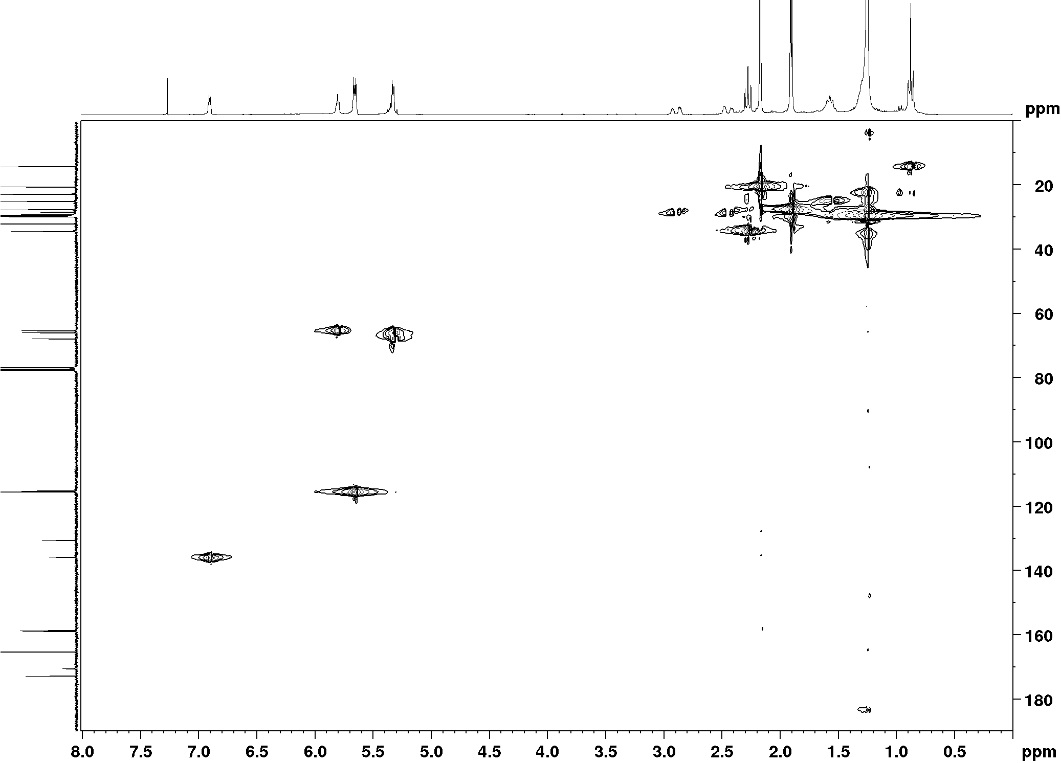
 **Figure S9.** HSQC spectrum (CDCl_3_, 300 and 75 MHz) of compound **1.**


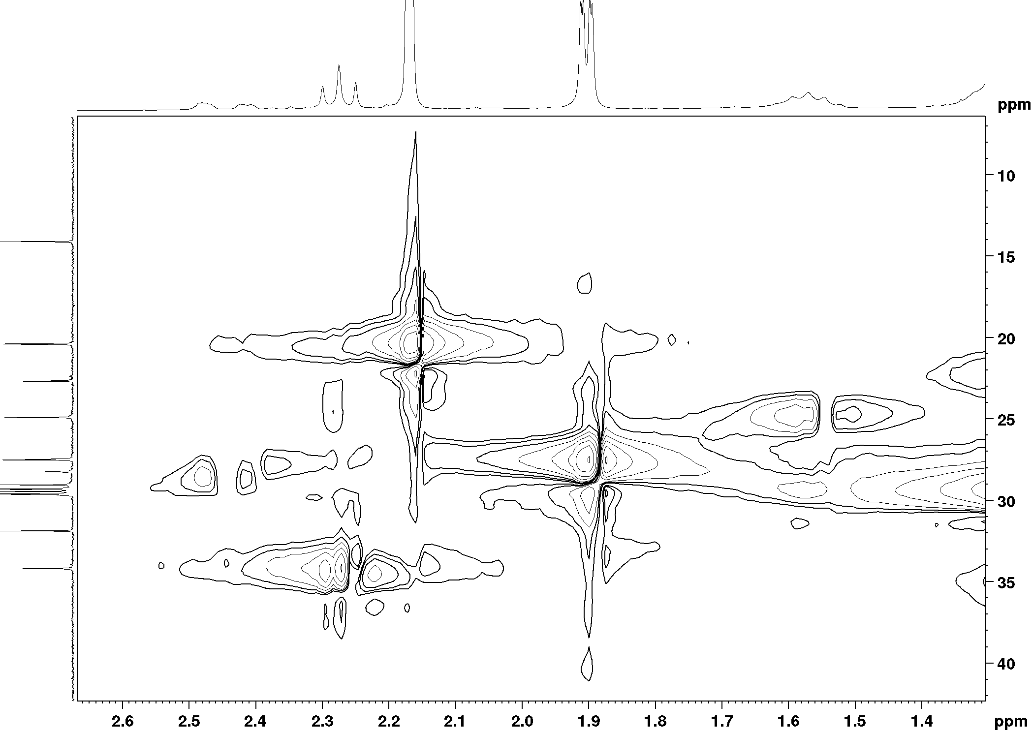

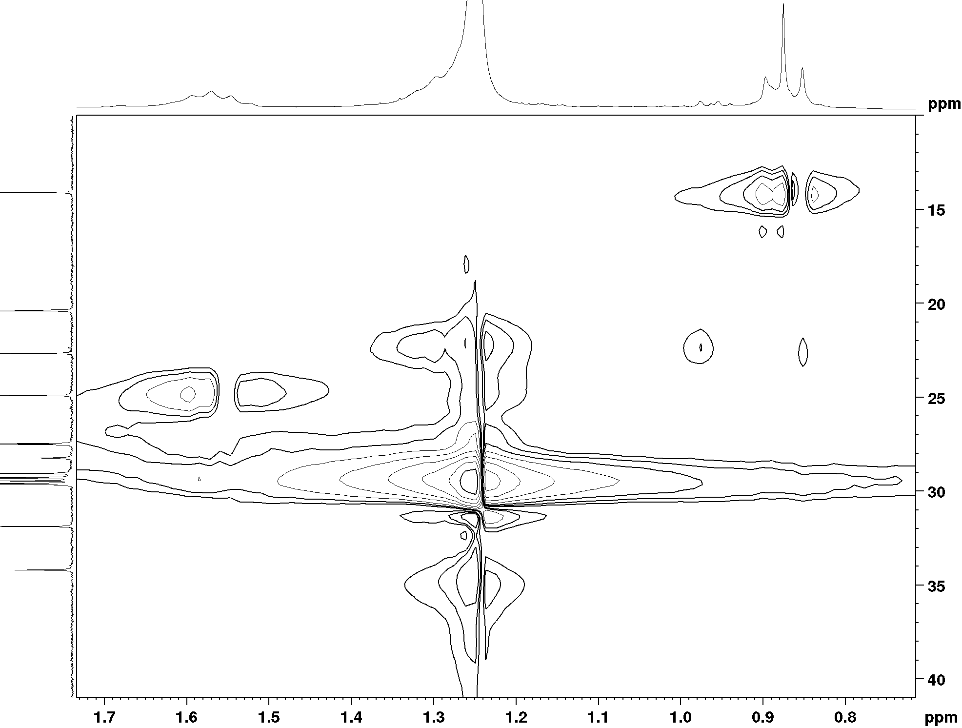


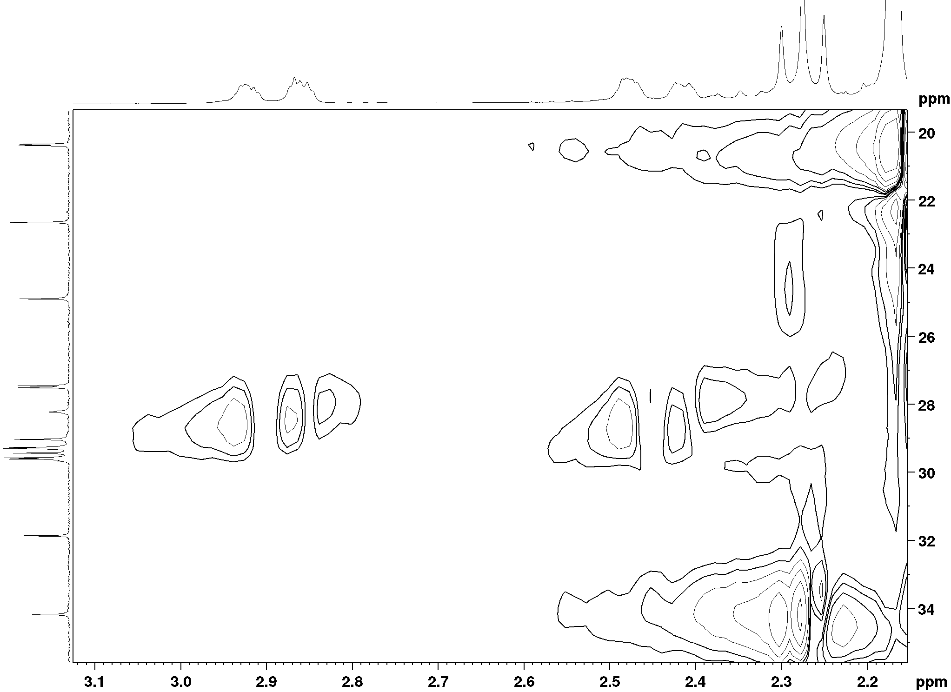

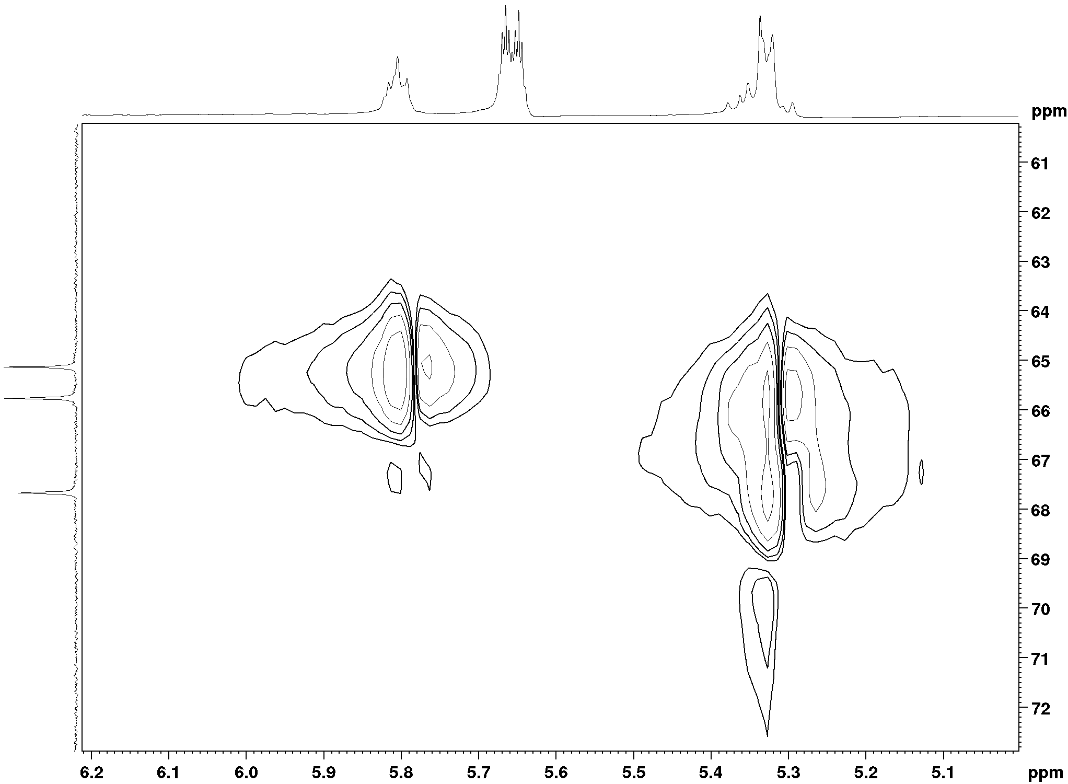

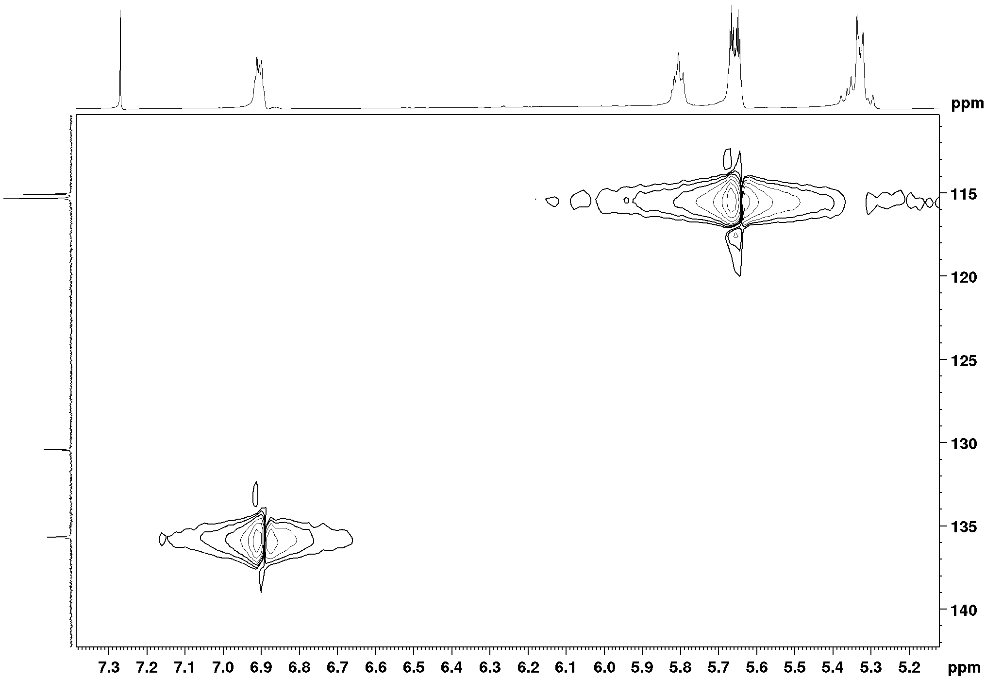


**Figure S10.** Expansions HSQC spectrum (CDCl_3_, 300 and 75 MHz) of compound **1.**

**
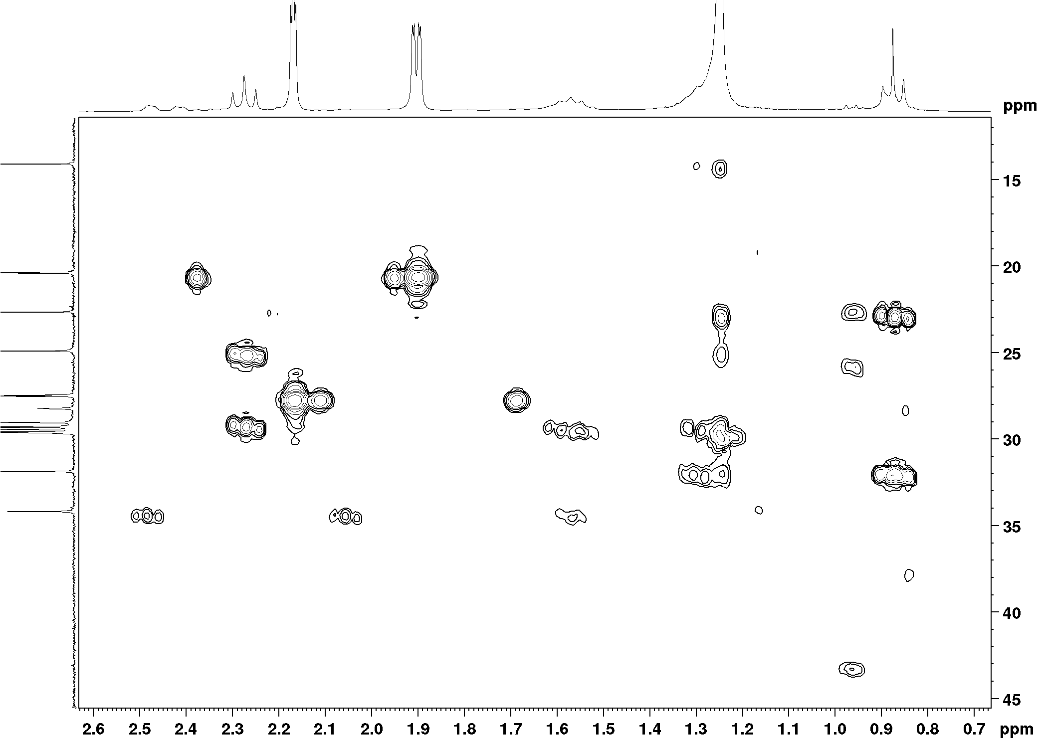

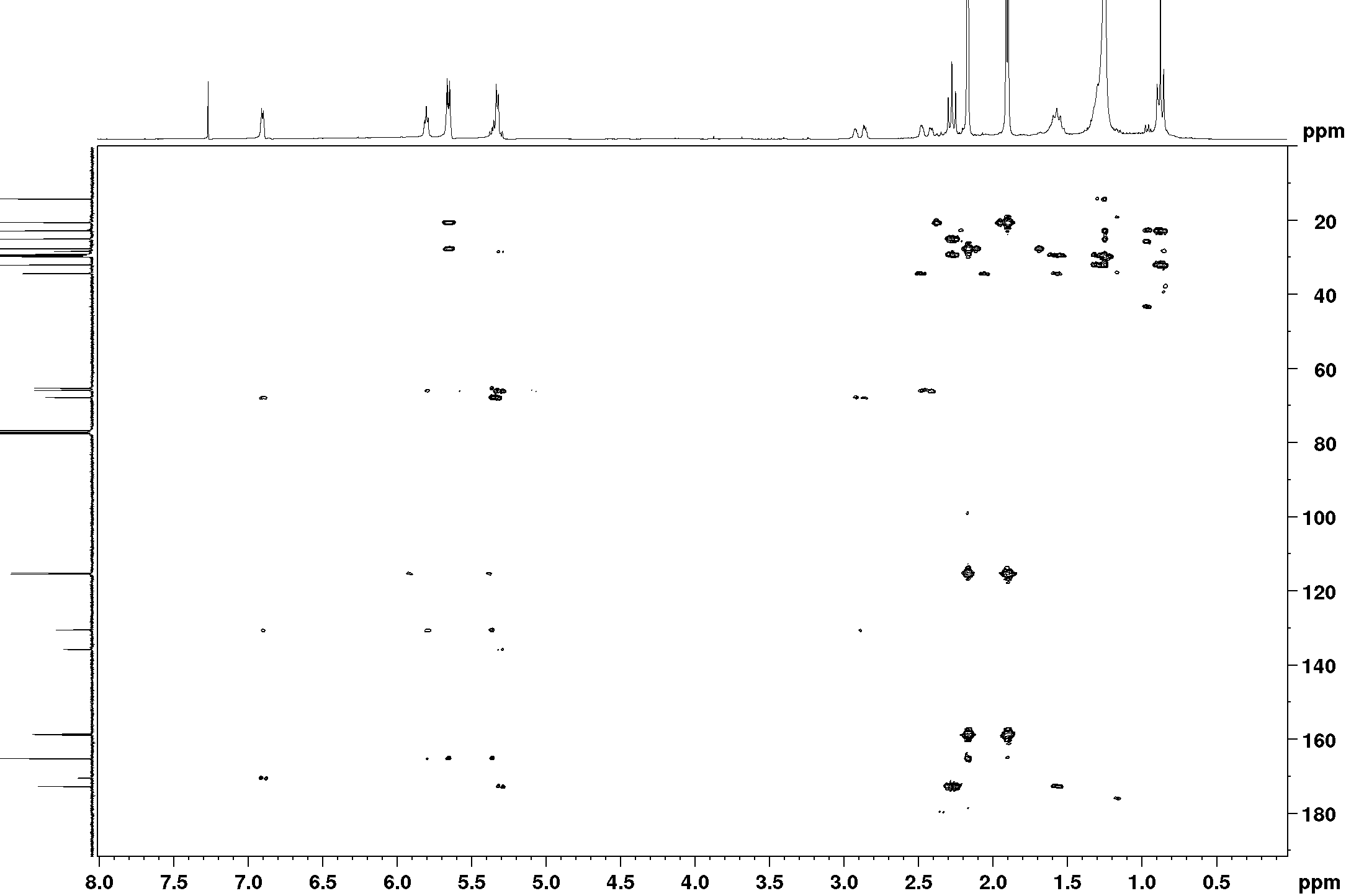
Figure S11**. HMBC spectrum (CDCl_3_, 300 and 75 MHz) of compound **1.**

**
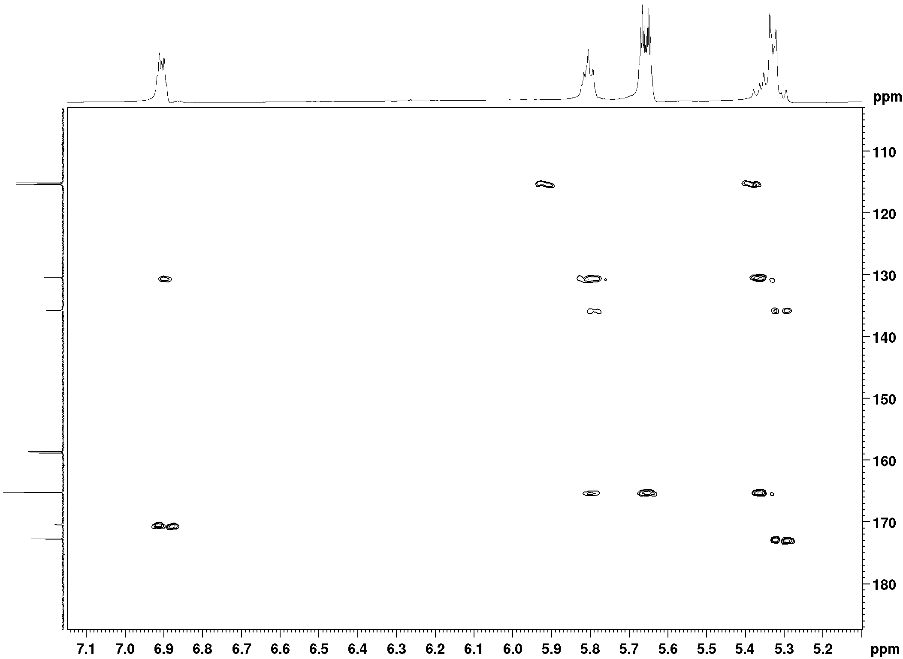

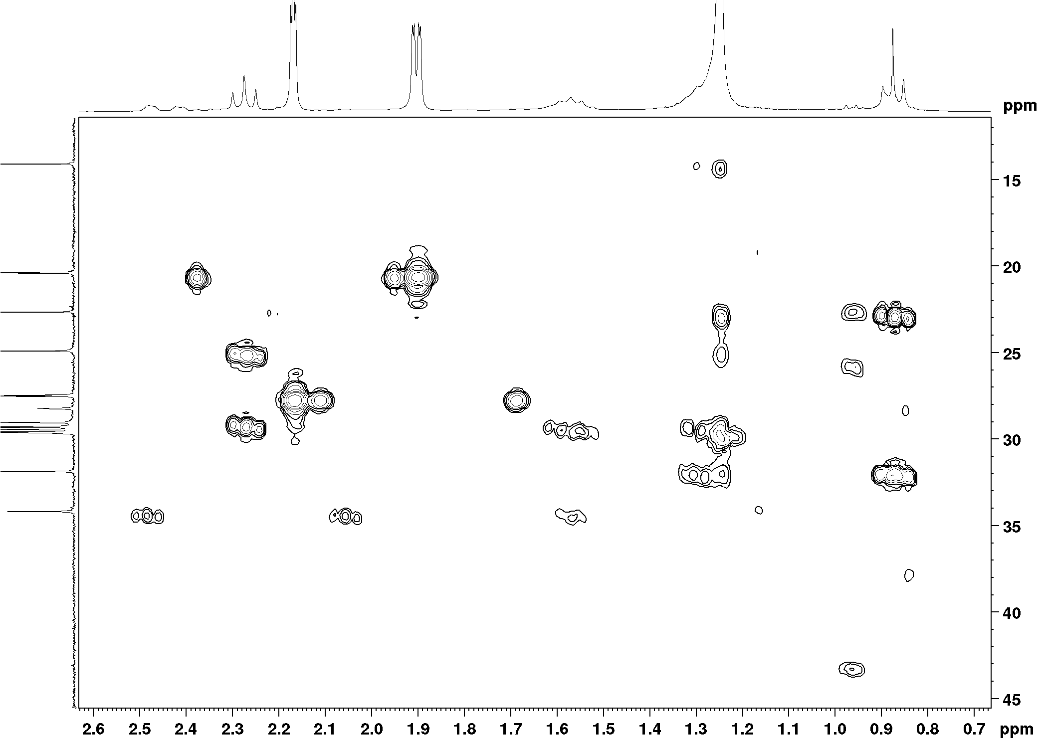

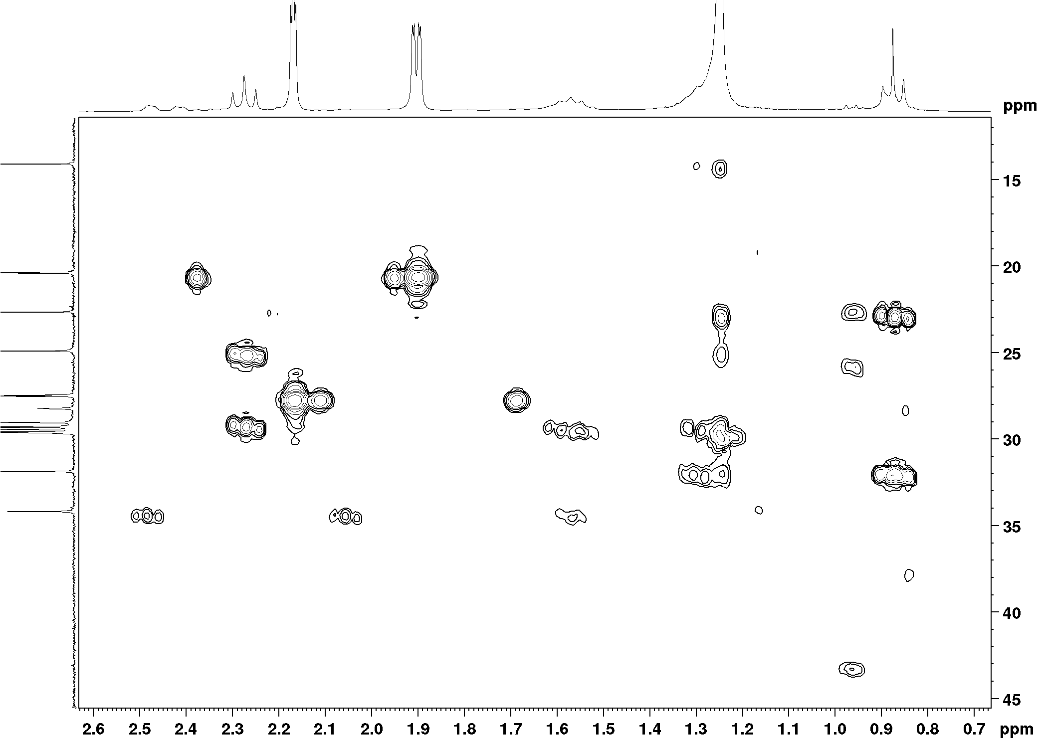
**

**Figure S12.** Expansions HMBC spectrum (CDCl_3_, 300 and 75 MHz) of compound **1.**

[**Figure S13.**](#_Toc58256854) MS/MS fragmentation spectrum for compound **1** (m/z 519.2932 -[M - H]^-^).


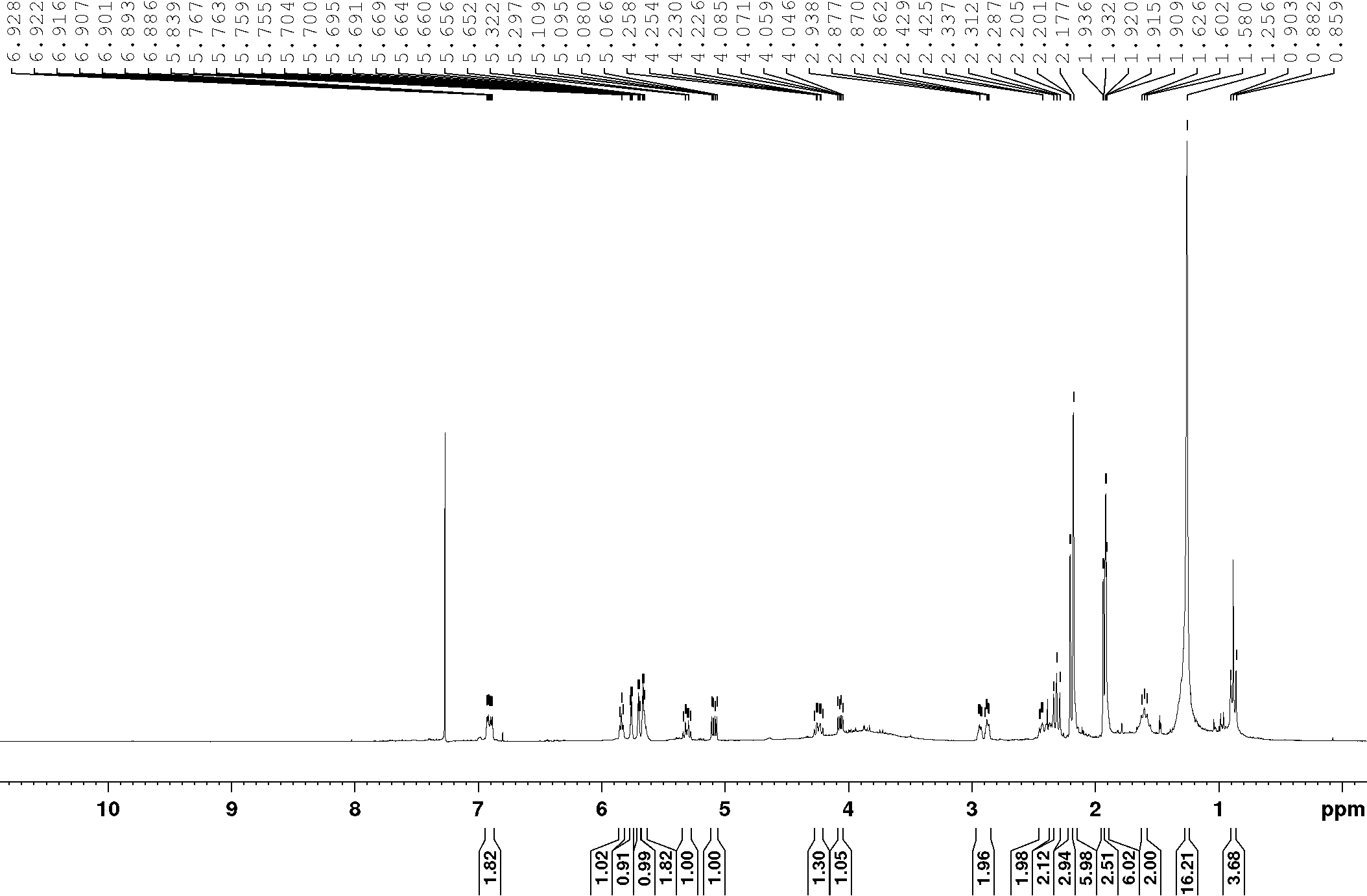
 **Figure S14.** ^1^H-NMR spectrum (CDCl_3_, 300 MHz) of compounds **2** and **3**.

**
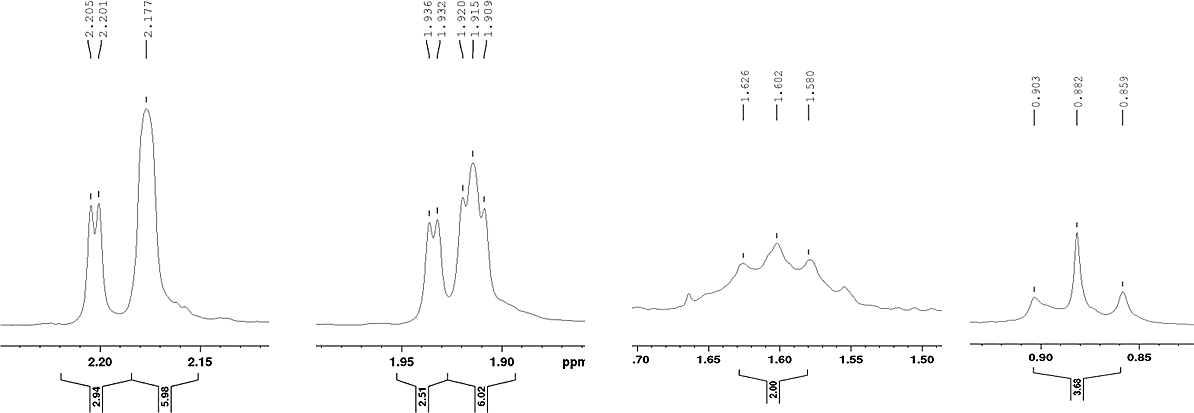
**[**Figure S15**. Expansions ^1^H-NMR spectrum (CDCl_3_, 300 MHz) of compounds **2** and **3**.](#_Toc58256842)

**
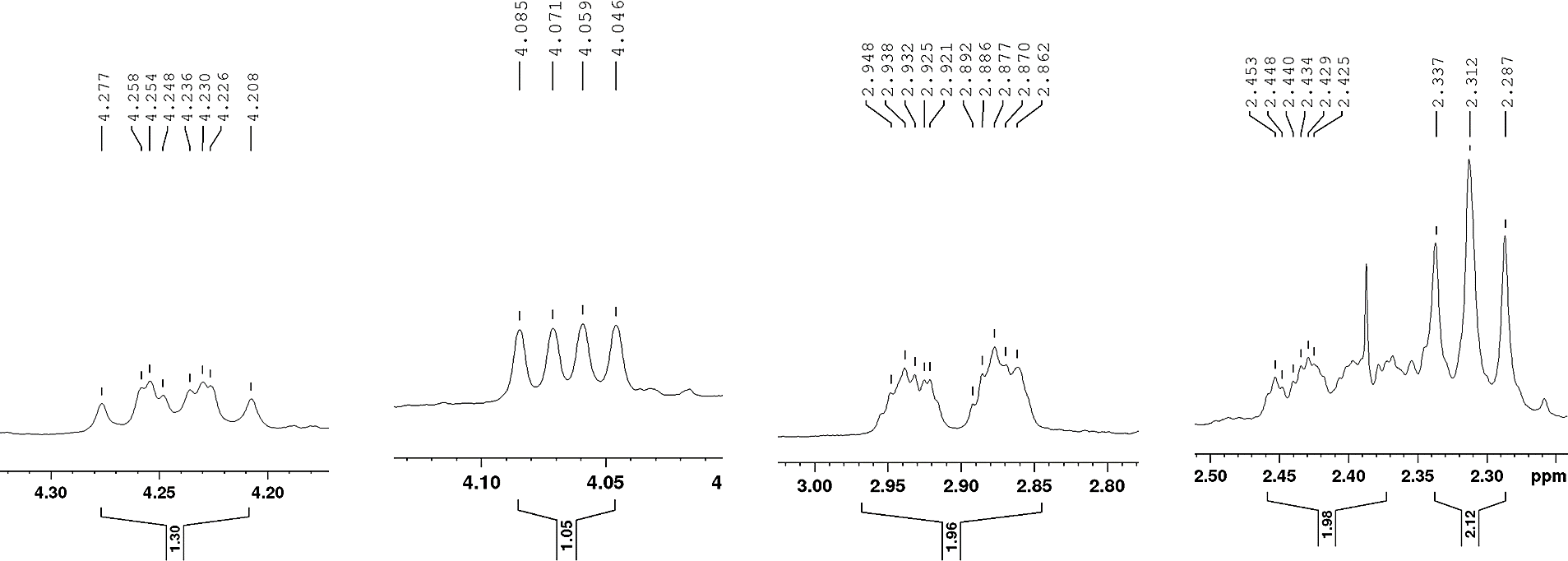
**[**Figure S16.** Expansions ^1^H-NMR spectrum (CDCl_3_, 300 MHz) of compounds](#_Toc58256843) **2** and **3**.

**
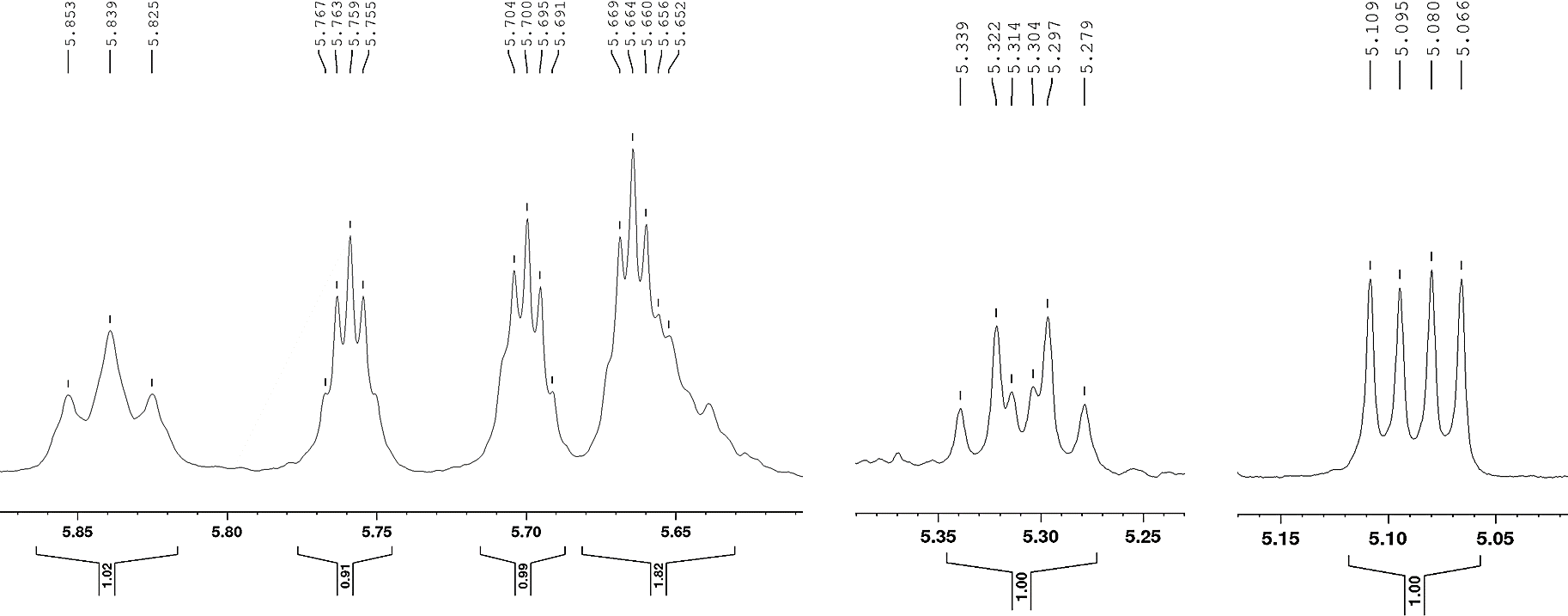
**

**
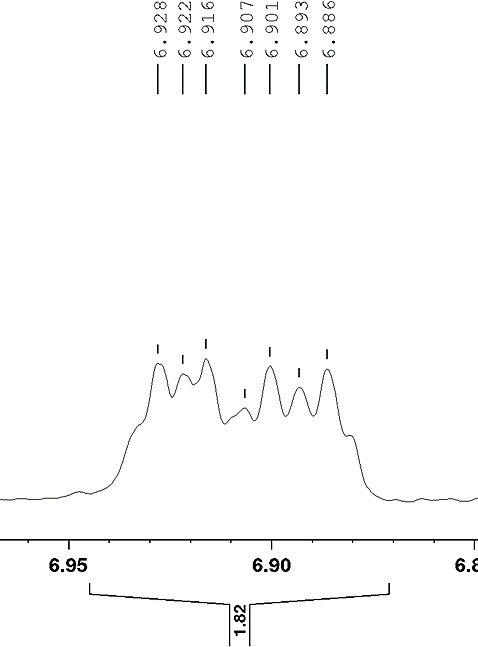
**

**Figure S17.** Expansions ^1^H-NMR spectrum (CDCl_3_, 300 and 75 MHz) of compound **1.**


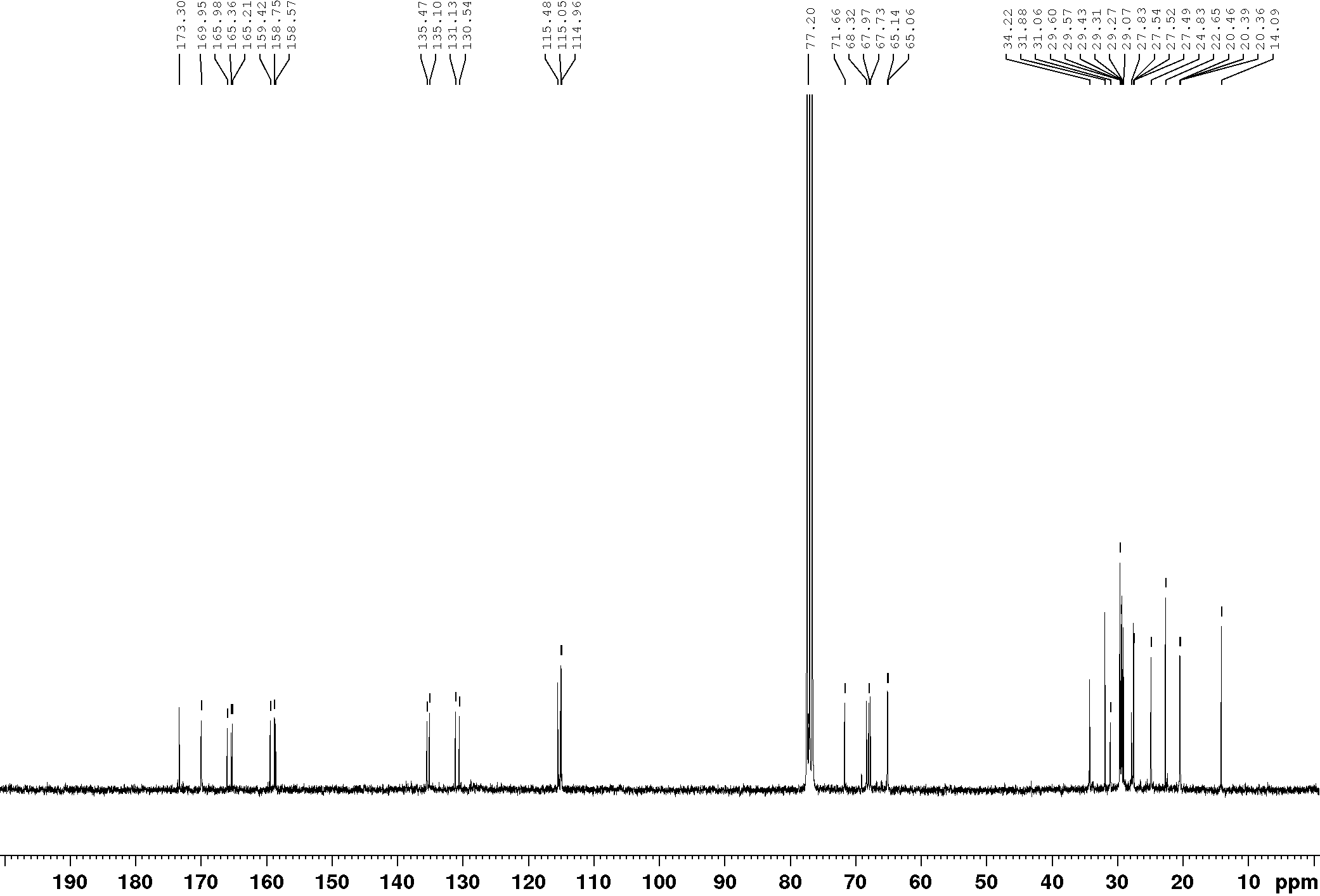
[**Figure S18.** ^13^C-NMR spectrum (CDCl_3_, 75 MHz) of compounds](#_Toc58256845) **2** and **3**.


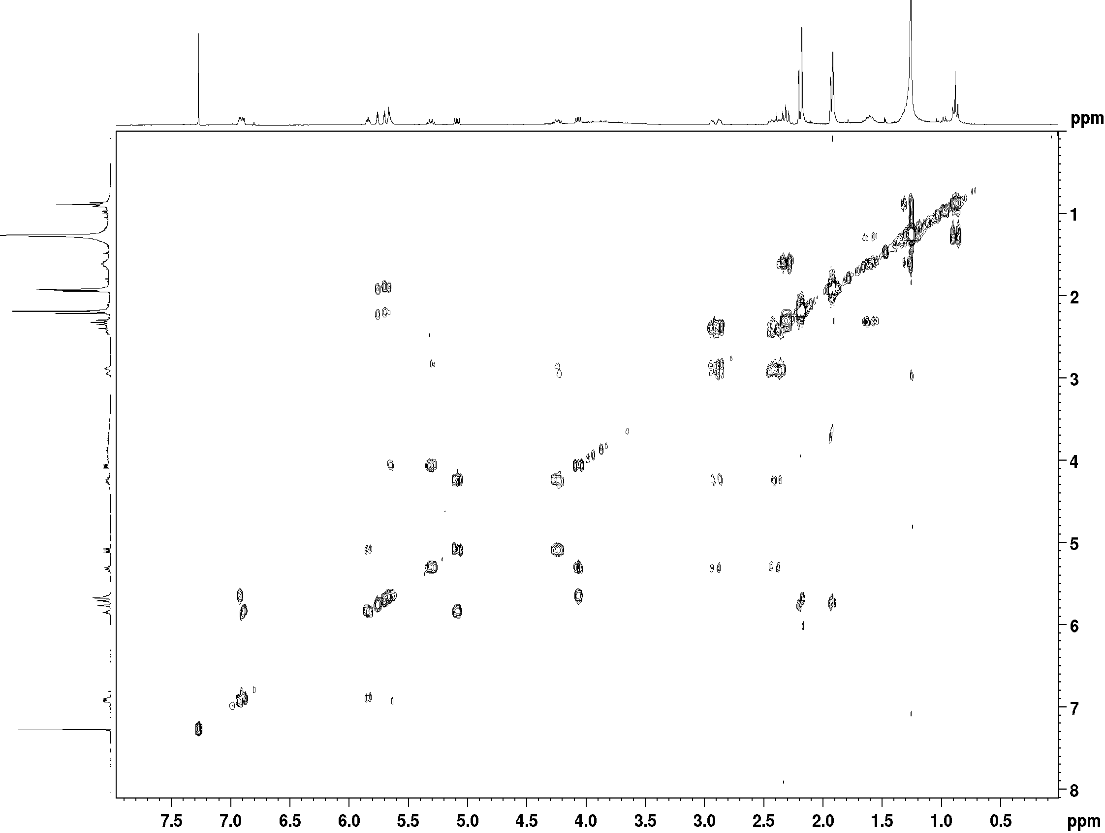
 **Figure S19**. COSY spectrum (CDCl_3_, 300 MHz) of compounds **2** and **3**.


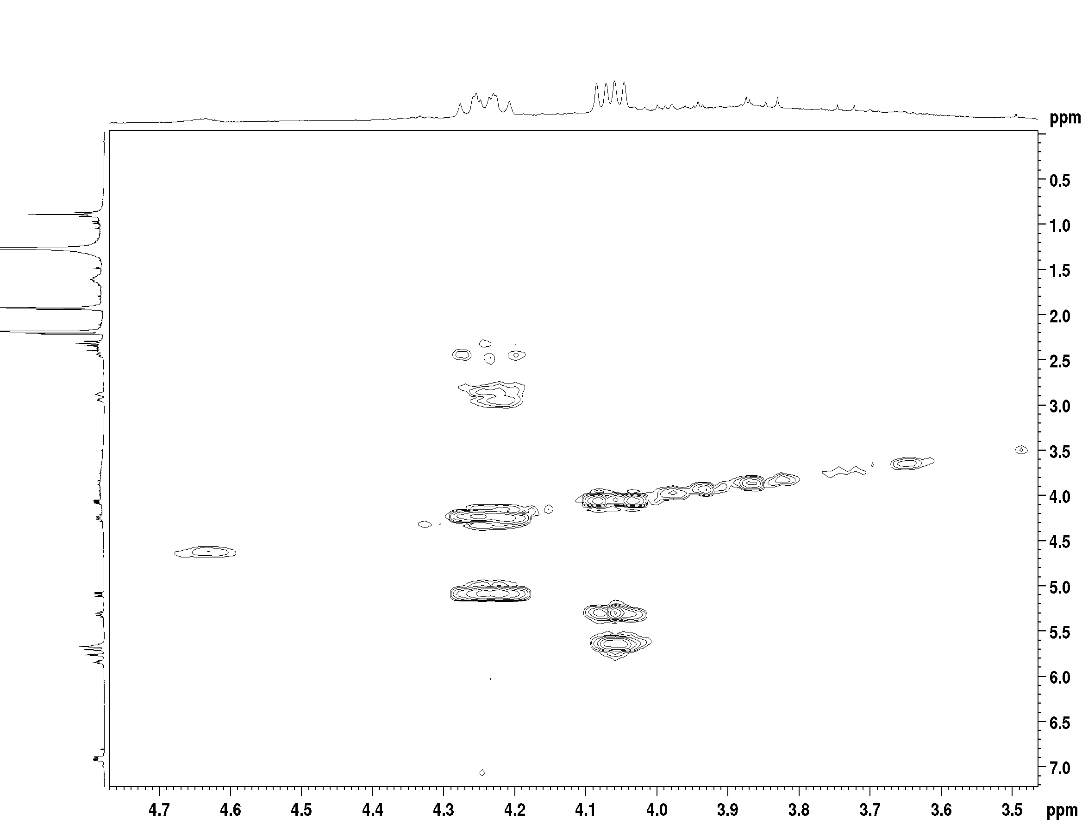

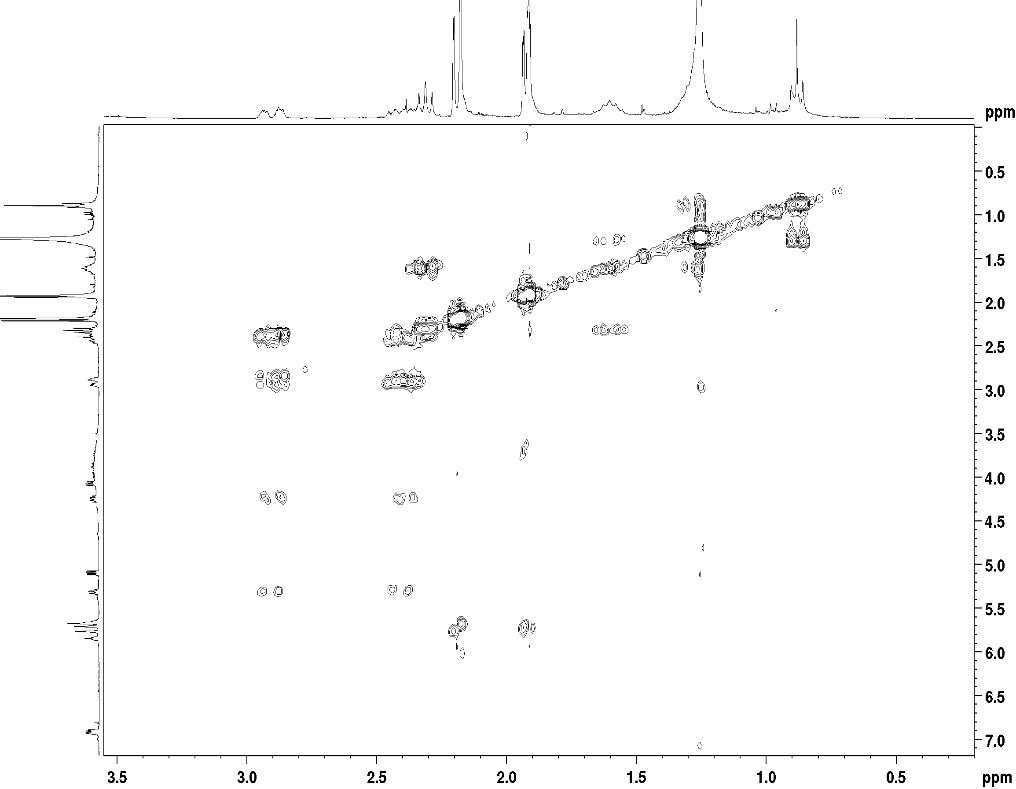


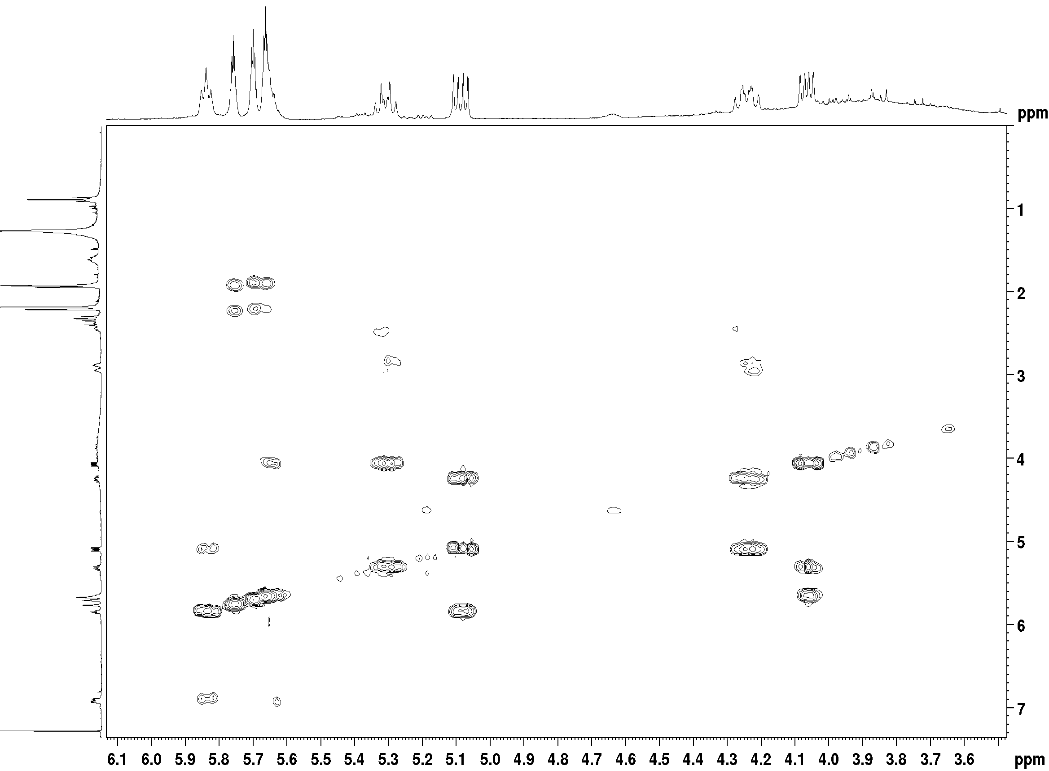


**Figure S20**. Expansions COSY spectrum (CDCl_3_, 300 MHz) of compound **2** and **3**.


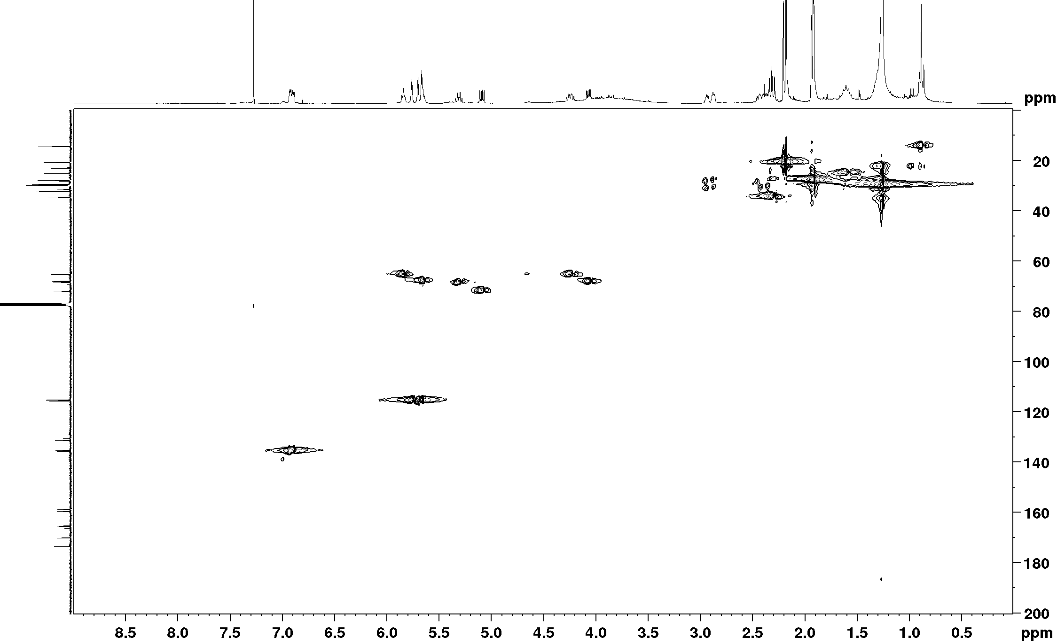
 **Figure S21.** HSQC spectrum (CDCl_3_, 300 and 75 MHz) of compounds **2** and **3**.


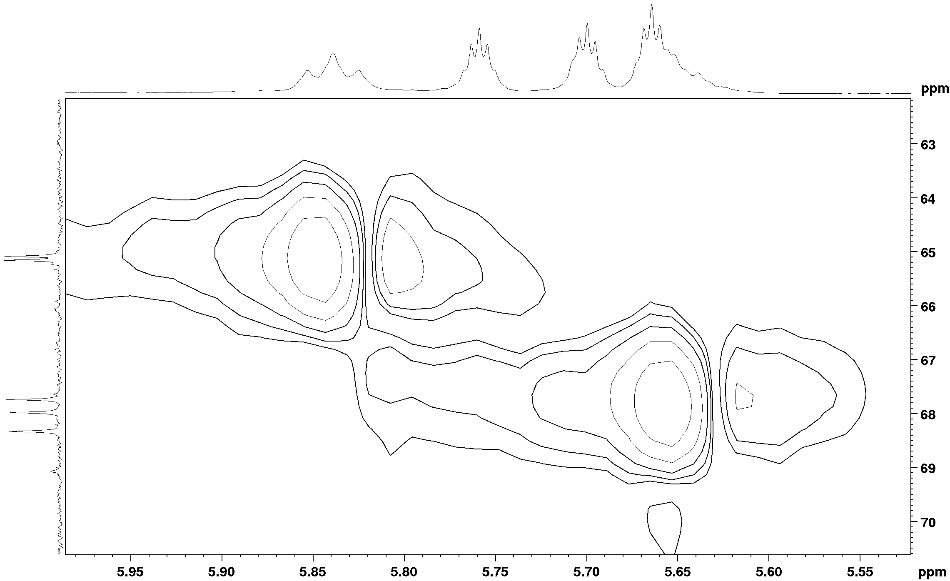

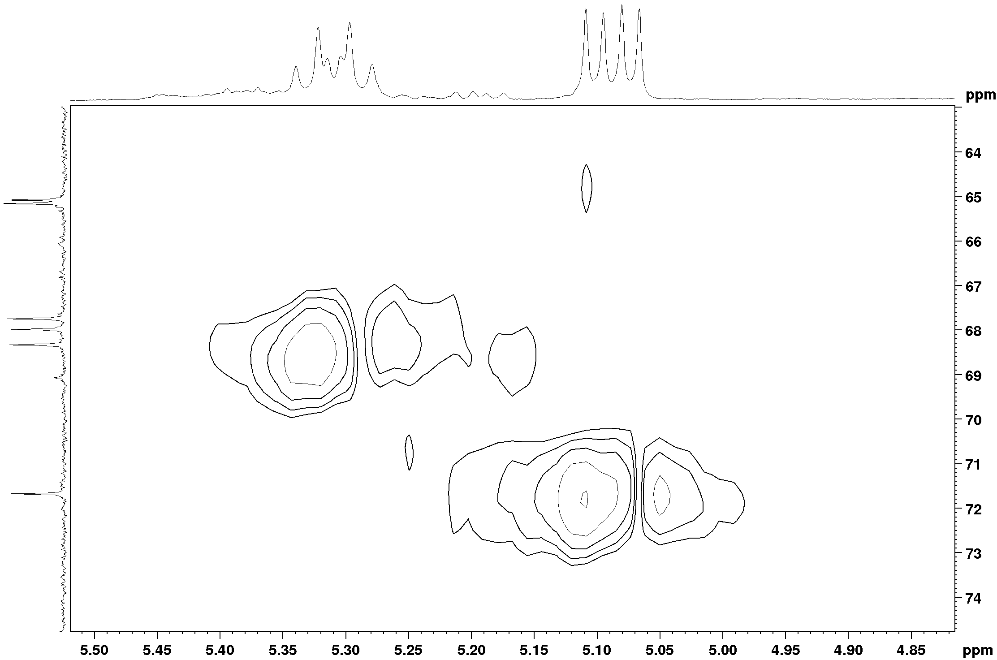

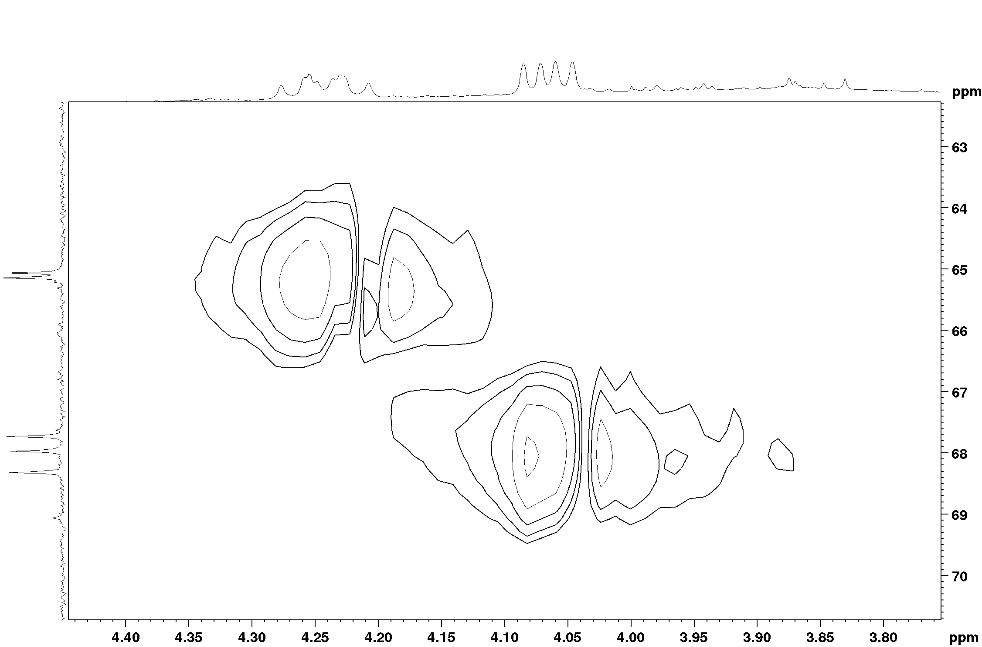

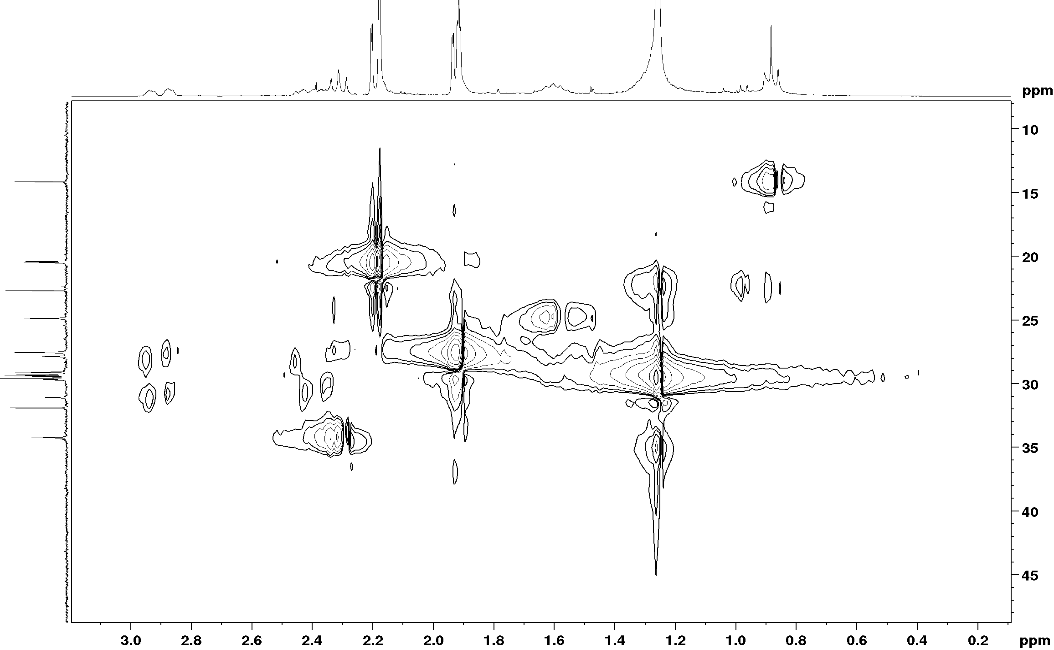

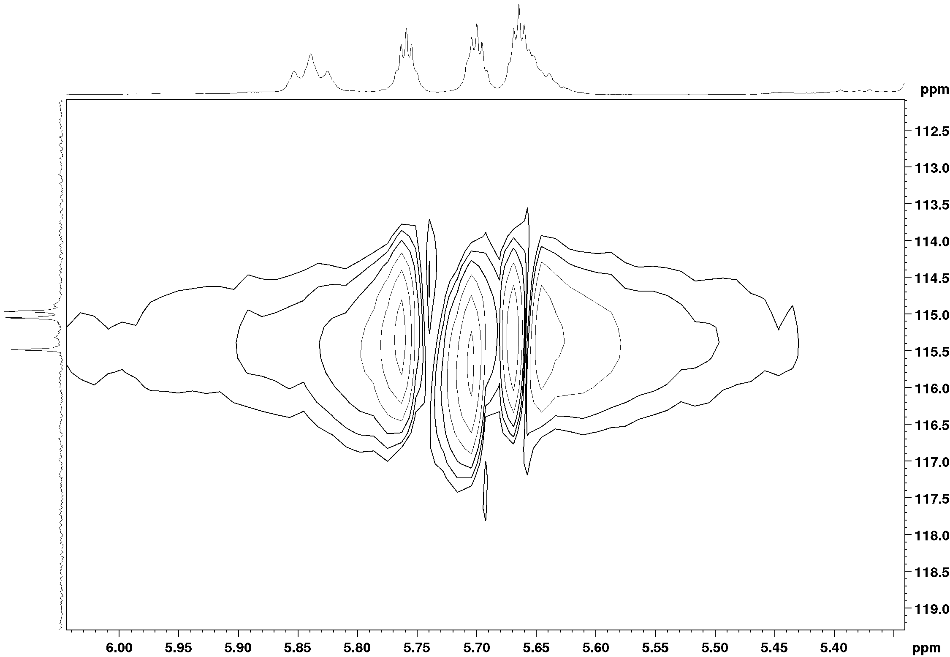

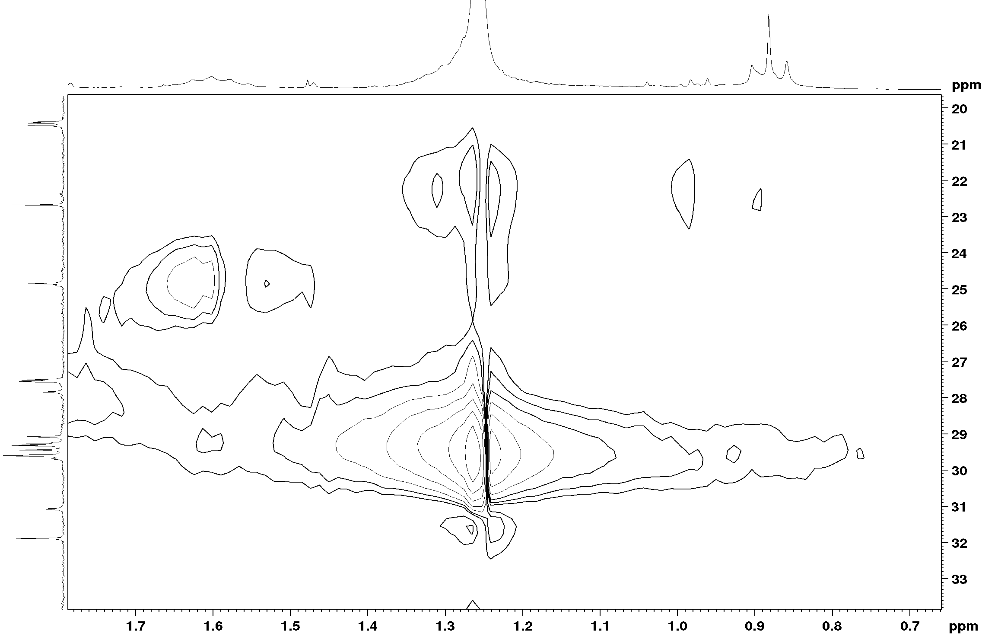


**Figure S22**. Expansions HSQC spectrum (CDCl_3_, 300 and 75 MHz) of compound **2** and **3.**


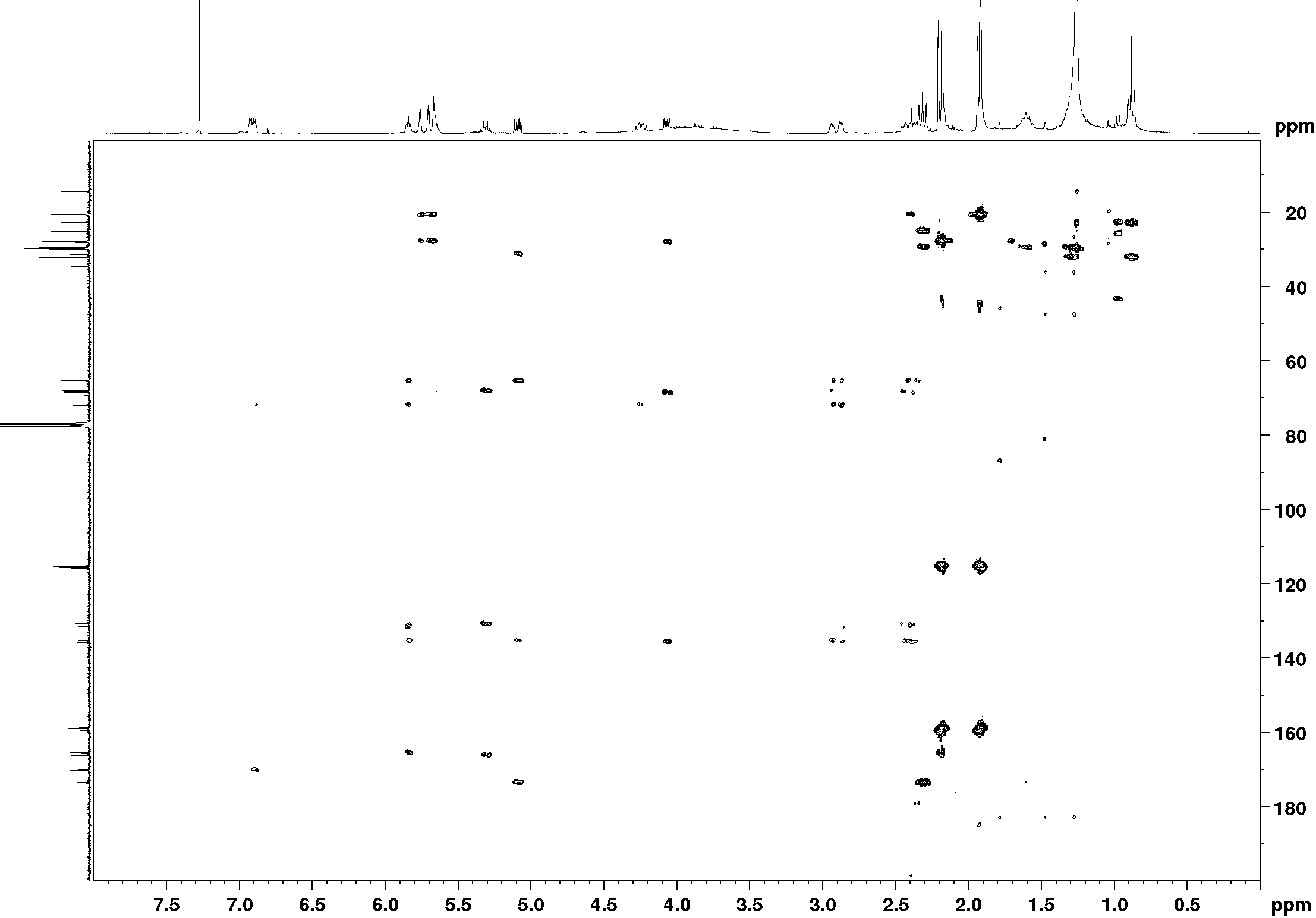
[**Figure S23**. HMBC spectrum (CDCl_3_, 300 and 75 MHz) of compounds **2** and **3**.](#_Toc58256853)


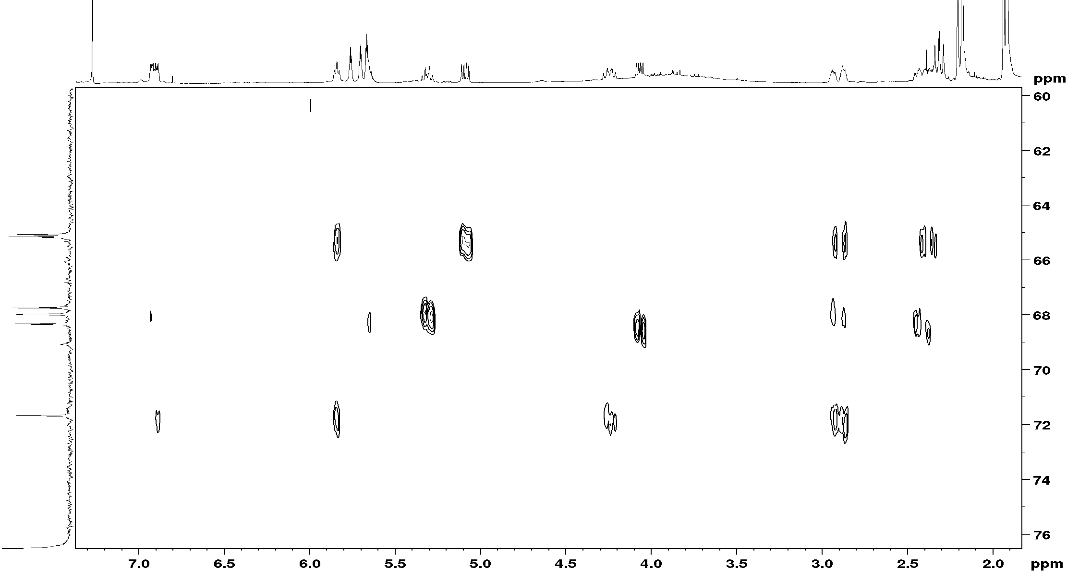


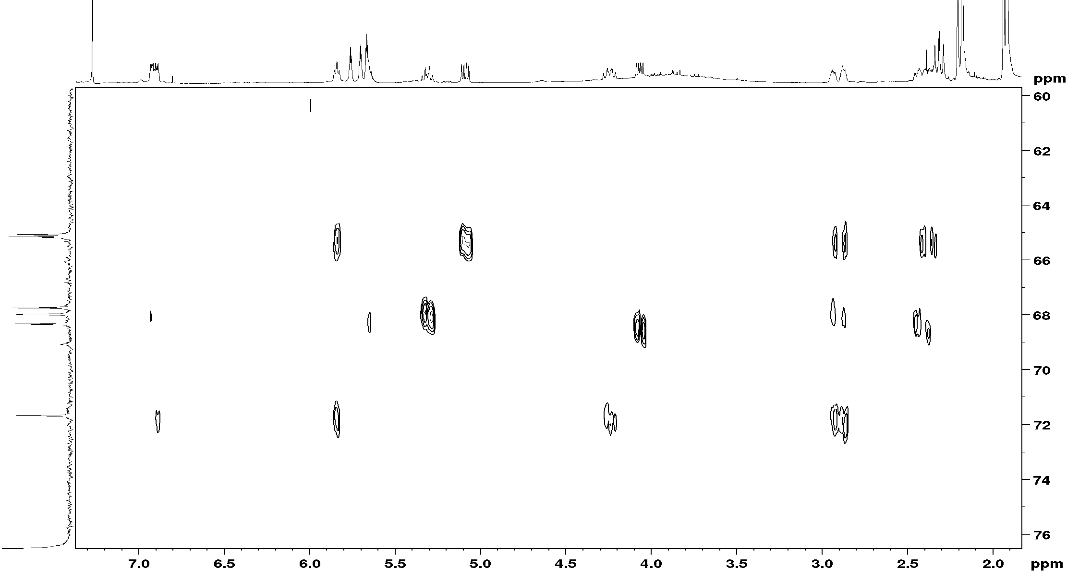


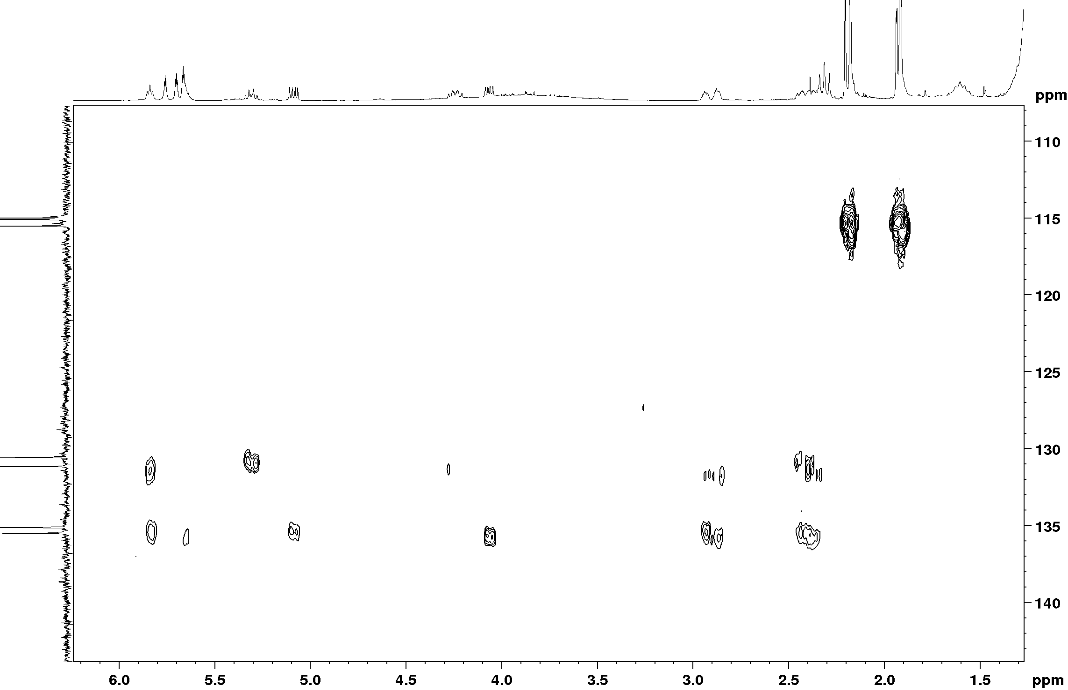


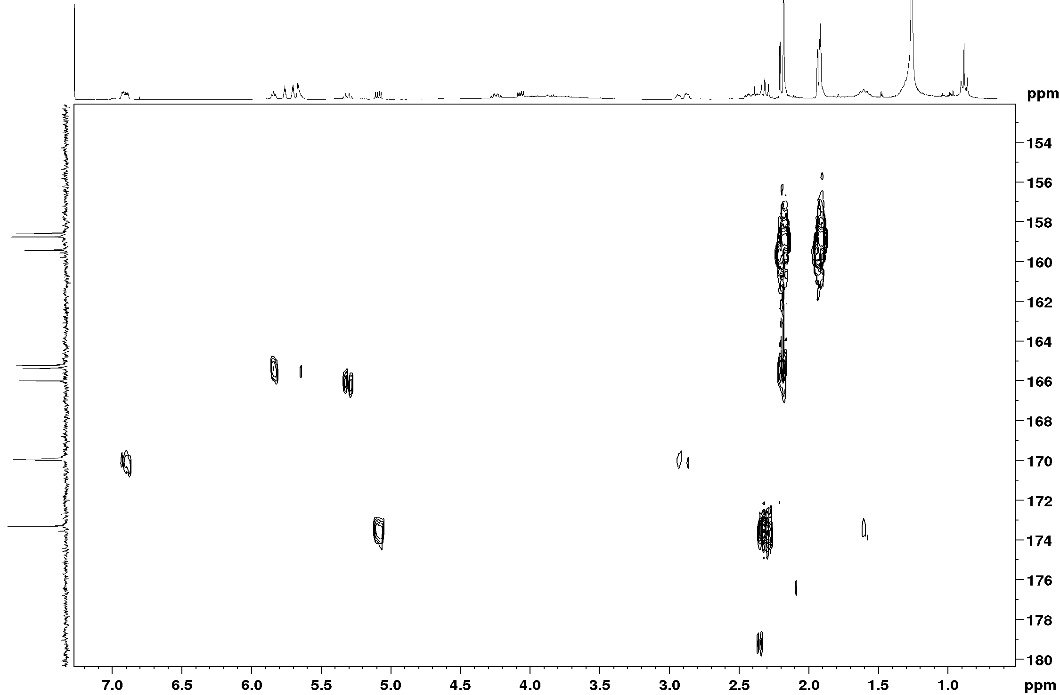


**Figure S24**. Expansions HMBC spectrum (CDCl_3_, 300 and 75 MHz) of compound **2** and **3**.

[**Figure S25.**](#_Toc58256854) MS/MS fragmentation spectrum for compound **2** (m/z 437.2524 -[M - H]^-^).

[**Figure S26.**](#_Toc58256854) MS/MS fragmentation spectrum for compound **3** (m/z 337.1272 -[M - H]^-^).

Structures and spectroscopic data of compounds **4– 6** isolated from aerial parts of *S. oleosus.*

Compound **4**: ^1^H NMR (300 MHz, CD_3_OD): δ 6.27 (1H, d, *J=* 1.2 Hz, H-2), 5.88 (1H, qd, *J=* 7.2 and 1.2 Hz, H-20), 5.49-5.53 (1H, m, H-7), 5.50 (1H, d, *J=* 12.0 Hz, H-9a), 4.79 (1H, d, *J=* 5.6 Hz, H-8), 4.67 (1H, d, *J=* 17.1 Hz, H-3a), 4.34 (1H, d, *J=* 16.1 Hz, H-3b), 4.29 (1H, d, *J=* 12.0 Hz, H-9b), 3.71-3.87 (2H, m, H-5), 3.64 (2H, s, H-18), 2.77-2.88 (1H, m, H-6a), 2.46 (1H, dquint, *J=* 14.3 and 2.8 Hz, H-6b), 2.26 (1H, d, *J=* 12.8 Hz, H-14a), 1.84 (3H, dd, *J=* 7.2 and 1.6 Hz, H-21), 1.79 (1H, d, *J=* 12.7 Hz, H-14a), 1.69-1.75 (1H, m, H-13), 0.83 (3H, d, *J=* 6.4 Hz, H-19). ^13^C NMR (75 MHz, CD_3_OD): δ 176.1 (C-11), 168.1 (C-16), 138.0 (C-20), 133.2 (C-15), 132.2 (C-2), 130.9 (C-1), 97.3 (C-8), 82.9 (C-12), 79.2 (C-3), 75.1 (C-7), 69.7 (C-5), 68.2 (C-18), 60.7 (C-9), 39.1 (C-14), 37.3 (C-13), 33.9 (C-6), 15.4 (C-21), 11.7 (C-19).

Compound **5:** ^1^H NMR (300 MHz, CD_3_OD): δ 7.73 (1H, dd, *J=* 8.8; 1.9 Hz, H-2’), 7.63 (1H, dd, *J=* 8.5 and 1.9 Hz, H-6’), 6.88 (1H, d, *J=* 8.5 Hz, H-5’), 6.38 (1H, d, *J=* 2.4 Hz, H-8), 6.18 (1H, d, *J=*2.4 Hz, H-6). ^13^C NMR (75 MHz, CD_3_OD): δ 177.3 (C-4), 165.5 (C-7), 162.4 (C-5), 158.2 (C-9), 148.7 (C-3’), 147.9 (C-2), 146.2 (C-4’), 137.2 (C-3), 124.1 (C-1’), 121.6 (C-6’), 116.2 (C-5’), 115.9 (C-2’), 104.5 (C-10), 99.2 (C-6), 94.3 (C-8).

Compound **6:** ^1^H NMR (300 MHz, CD_3_OD): δ 7,49 (1H, d, *J=* 15,8 Hz, H-7’’), 7,45 (1H, d, *J=* 15,8 Hz, H-7’), 7,06 (1H, d, *J=* 2,0 Hz, H-2’’), 7,03 (1H, d, *J=* 2,0 Hz, H-2’), 6,95-6,99 (1H, m, H-6’ and H-6’’), 6,77 (1H, d, *J=* 8,1 Hz, H-5’’), 6,75 (1H, d, *J=* 8,1 Hz, H-5’), 6,24 (1H, d, *J=* 15,8 Hz, 8’), 6,21 (1H, d, *J=* 15,8 Hz, H-8’’), 5,32 (1H, td, *J=* 10,4 and 4,5 Hz, H-5), 5,15 (1H, d, *J=* 3,1 Hz, H-3), 3,71 (1H, d, *J=* 9,6 Hz, H-4), 2,11 (1H, d, *J=* 12,8 Hz, H-2a), 1,90 (1H, t, *J=* 12,2 Hz, H-6a), 1,75 (1H, d, *J=* 13,7 Hz, H-2b and H-6b). ^13^C NMR (75 MHz, CD_3_OD): δ 177.4 (C-7), 166.5 (C-9’’), 166.2 (C-9’), 148.4 (C-4’), 148.2 (C-4’’), 145.6 (C-3’), 145.7 (C-3’’), 144.7 (C-7’), 144.4 (C-7’’), 125.7 (1’’), 125.6 (C-1’), 121.3 (C-6’), 121.0 (C-6’’), 115.9 (C-5’’), 115.8 (C-5’’), 115.2 (C-2’’), 114.8 (C-2’), 114.7 (C-8’), 114.6 (C-8’’),79.2 (C-1), 73.6 (C-3), 71.3 (C-5), 70.9 (C-4), 36.3 (C-3).

**SDC**

**Blank positive mode**

**SCE**

**SEA**

**SHM**

**Figure S27.** UHPLC-HR-MS/MS chromatograms of the crude extract and fractions of *Senecio oleosus* acquired in positive ionization mode (ESI+). Crude extract (SCE); dichloromethane fraction (SDC), ethyl acetate fraction (SEA) and hydromethanolic fraction (SHM).

**Blank negative mode**

**SCE**

**SDC**

**SEA**

**SHM**

**Figure S28.** UHPLC-HR-MS/MS chromatograms of the crude extract and fractions of *Senecio oleosus* acquired in negative ionization mode (ESI+). Crude extract (SCE); dichloromethane fraction (SDC), ethyl acetate fraction (SEA) and hydromethanolic fraction (SHM).

**Figure S29.** Possible structures for compounds **MS-10** to **MS-16.**

**Figure S30.** Possible structures for compounds **MS-17** to **MS-24.**

**Figure S31.** Possible structures for compounds **MS-25** to **MS-31**.

**Figure S32.** Possible structures for compounds **MS-32** to **MS-42.**

**Figure S33.** Possible structures for compounds **MS-43** to **MS-45.**

**Figure S34.** Anti-proliferative profile of compound **1** obtained from *Senecios oleosus* aerial parts and doxorubicin (positive control) using MTT assay on HCT-116 (colorectal adenocarcinoma), 501mel (metastatic melanoma) and MCF-7 (breast adenocarcinoma) after 72h-exposure. IC_50_ and CI95 values were determined by non-linear regression using GraphPad Prism software v.9.0. Results reflected the mean of three independent experiments (n=3).

**Table S1**. Diagnostic ions used to identify tri-esterified shikimic acid derivatives in GNPS2 platform.

| **R_1_** | **Diagnostic ion 1 (m/z)**  M*_di1_* = 120.02 + M*_ac_* – 1 | **Diagnostic ion 2 (m/z)**  M*_di2_* = M*_di1_* – 44 | **Diagnostic ion 3 (m/z)**  M*_di3_* = M*_ac_* – 1 |
| --- | --- | --- | --- |
| Lauric acid  (M*_ac_* = 200.31) |  |  |  |
| Capric acid  (M*_ac_* = 172.26) |  |  |  |
| Caprylic acid  (M*_ac_* = 144.21) |  |  |  |
| Senecioic acid  (M*_ac_* = 100.11) |  |  |  |
| Cinnamic acid  (M*_ac_* = 148.15) |  |  |  |
| *p*-coumaric acid  (M*_ac_* = 164.05) |  |  |  |

*di =* diagnostic ion; *ac* = acid

**Table S2**. Inhibition rate (%) of samples submitted for antiproliferative testing against HCT-116 (colon carcinoma), 501mel (metastatic melanoma) and MCF-7 (breast adenocarcinoma) cells at of 5 and 50 µg mL^-1^ concentrations, determined by the MTT assay after 72hs of incubation. Data reflect the mean ± standard deviation (SD) of three independent experiments (n=3) and were analyzed in GraphPad Prism Software v.9.0. Comp. = abbreviation for Compound.

| **Samples** | **HCT-116** | | **501mel** | | **MCF-7** | |
| --- | --- | --- | --- | --- | --- | --- |
|  | **5 µg mL^-1^** | **50 µg mL^-1^** | **5 µg mL^-1^** | **50 µg mL^-1^** | **5 µg mL^-1^** | **50 µg mL^-1^** |
|  | **Inhibition rate ± SD** | | | | | |
| **SCE** | 24.3 ± 7.1 | 97.7 ± 3.3 | 39.1 ± 25.9 | 99.9 ± 0.3 | 36.5 ± 24.5 | 96.8 ± 4.4 |
| **SHE** | 15.9 ± 10.8 | 99.4 ± 0.9 | 5.1 ± 4.4 | 98.7 ± 1.7 | 5.6 ± 5.8 | 98.3 ± 0.8 |
| **SDC** | 61.3 ±18.4 | 98.6 ± 1.8 | 77.4 ± 19.1 | 99.6 ± 0.3 | 61.8 ± 29.3 | 94.9 ± 3.0 |
| **SAE** | 2.1 ± 9.2 | 5.02 ± 5.6 | 0 ± 4.1 | 0 ± 2.4 | 0 ± 4.9 | 0 ± 1.3 |
| **SHM** | 10.7 ± 8.2 | 0.5 ± 8.8 | 0 ± 9.3 | 0 ± 6.8 | 0.4 ± 1.8 | 1.3 ± 3.7 |
| **Comp. 1** | 80.8 ± 12.7 | 99.6 ± 0.7 | 69.6 ± 12.1 | 98.3 ± 1.3 | 53.1 ± 13.2 | 97.9 ± 0.9 |
| **Comp. 2 + 3** | 18.8 ± 8.2 | 96.7 ± 2.1 | 15.5 ± 7.8 | 92.7 ± 7.8 | 13.5 ± 4.6 | 69.2 ± 35.7 |
| **Comp. 4** | 1.1 ± 9.8 | 8.1 ± 2.3 | 8.6 ± 9.4 | 17.0 ± 10.1 | 15.5 ± 6.0 | 16.5 ± 7.3 |
| **Comp. 5** | 20.3 ± 5.6 | 61.1 ± 10.9 | 27.1 ± 4.2 | 69.1 ± 2.3 | 2.0 ± 6.8 | 33.6 ± 3.7 |
| **Comp. 6** | 0 ± 7,4 | 0 ± 7.7 | 0 ± 4.9 | 0 ± 7.0 | 0 ± 4.3 | 0 ± 5.4 |

**Table S3.** Half-maximal inhibitory concentration (IC_50_) and 95% confidence interval (CI_95_) of compound 1 (μg mL^-1^ and µM) and doxorubicin (µM) against tested cell lines (HCT-116, 501mel and MCF-7), determined by the MTT assay after 72h of incubation. Values were calculated by non-linear regression using GraphPad Prism software v.9.0, and reflected the mean of three independent experiments (n=3).

|  | **Doxorubicin (µM)** | | | **Compound 1 (µg mL^-1^)** | | | **Compound 1 (µM)** | | |
| --- | --- | --- | --- | --- | --- | --- | --- | --- | --- |
|  | **HCT-116** | **501mel** | **MCF-7** | **HCT-116** | **501mel** | **MCF-7** | **HCT-116** | **501mel** | **MCF-7** |
| **IC_50_** | 0.14 | 0.20 | 0.34 | 10.5 | 13.35 | 11.5 | 20.2 | 25.6 | 22.08 |
| **CI_95_** | 0.4 - 0.1 | 0.14 – 0.27 | 0.22 – 0.52 | ND | ND – 16.21 | 9.2 – 13.9 | ND | ND – 31.13 | 17.6 – 26.7 |
